# Co-N-Si/AC Catalyst for Aerobic Oxidation of Benzyl Alcohols to Esters under Mild Conditions

**DOI:** 10.3390/molecules26226792

**Published:** 2021-11-10

**Authors:** Changjian Zhou, Rong Sun, Yuting Zhang, Biao Xiong, Hui Dai, Yong Dai

**Affiliations:** 1 School of Chemistry and Chemical Engineering, Yancheng Institute of Technology, Yancheng 224051, China; zcj@ycit.cn (C.Z.); flowersprouts2021@163.com (R.S.); 2 School of Pharmacy, Nantong University, 19 Qixiu Road, Nantong 226001, China; 1923110159@stmail.ntu.edu.cn (Y.Z.); hsiung1987@ntu.edu.cn (B.X.); 3 College of Materials and Chemistry & Chemical Engineering, Chengdu University of Technology, Chengdu 610059, China

**Keywords:** aerobic oxidation, benzyl alcohols, heterogeneous cobalt

## Abstract

A stable, earth-abundant, reusable cobalt-based heterogeneous catalyst is developed for the oxidative esterification of alcohols under ambient conditions, featuring broad substrate scope, providing good to excellent product yields. This protocol enables easy recyclability of the catalyst, measured up to five times without significant loss of efficiency. The active sites of Co-N-Si/AC are proposed to be Co-N species.

## 1. Introduction

**Scheme 1:**
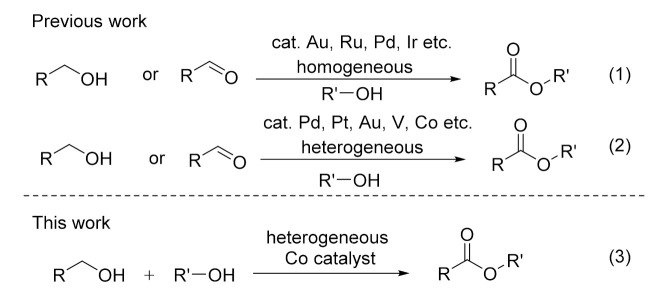
Synthesis of esters from alcohols.

Esters are a significant part of the building blocks in organic synthesis that are widely applied in pharmaceuticals, fine chemicals, natural products, fragrances and commercial products [1]. In recent decades, a great deal of research effort has been devoted to developing environmentally benign and cost-effective methods for the synthesis of esters from alcohols. This is easily accessible and of increasing importance as renewable plant-derived feedstock [2]; as alternatives for traditional protocols such as noble-metal-based homogeneous catalytic system such as gold, ruthenium, palladium and iridium [3,4,5,6,7,8,9,10,11]; and for the exploration of heterogeneous (palladium, platinum, gold, vanadium and cobalt supported) catalysts [12,13,14,15,16,17,18,19,20,21,22] (Scheme 1). Although these utilities are of significant practical importance, they always bear one or more shortcomings such as long reaction times, the use of precious metal catalysts, harsh reaction conditions and poor selectivity. Of all these protocols, direct oxidative esterification of alcohols with molecular oxygen catalyzed by heterogenous catalysts (ideally based on a non-noble metal) may be the most favored and promising for many reasons: (i) the use of oxygen as an oxidant reagent is green, economical and sustainable; (ii) the recyclability and reusability of heterogenous catalysts; (iii) the ease of separation; (iv) the ease of accessibility and renewability of alcohols; (v) the replacement of a noble metal with a base metal; etc. Moreover, an increasing emphasis on the development of cost-effective and environmentally friendly processes for the chemical industry is leading to continuous interest in the search for suitable catalytic systems for such transformations. Therefore, the exploration and development of efficient catalytic systems for oxidative esterification under mild conditions are of increasing interest in both green chemistry and organic synthesis.

Continuous effort has gone into research in the conversion of plentiful and sustainable alcohol resources into functional molecules [23,24,25,26,27,28] and the exploitation of heterogeneous base metal catalysts as well as the catalytical oxidant performance of related heterogeneous catalysts [29,30,31,32,33]. The natural amplitude of cobalt and its excellent performance in the oxidation reaction [34,35,36] as well as the capacity of silicon dioxide in regulating the micropore size of carbon material to facilitate mass transfer have been observed [37,38,39]. Here, we report a Co catalyst that permits the oxidative esterification of aromatic alcohols with alcohols in the presence of halogen, nitrile, nitro, and amino functional groups under mild conditions. Compared with previous work reported by Jagadeesh, the catalytic material prepared by us has better catalytic performance, demonstrated by a shorter reaction time and by a lower reaction temperature. More importantly, we describe the adhibition of a heterogeneous cobalt-based catalytic system for the direct synthesis of esters with aromatic alcohols that is tolerant of functional groups and that provides moderate to good yields of the target compounds with high selectivity. Notably, the use of molecular oxygen as the final oxidant and heterogeneous Co catalyst, which is easily recycled and conveniently reused, constitutes an important part in developing practical and economical catalytic processes.

## 2. Results and Discussion

Generally, the synthesis of the cobalt catalyst was performed as follows: Co(OAc)_2_·4H_2_O and 1,10-phenanthroline were stirred in ethanol at 60 °C for 2 h to generate the cobalt complex in situ. Silica was then introduced into the above solution by in situ hydrolysis of the added Si(OC_2_H_5_)_4_ (TEOS) with aqueous ammonia. After mixing, the activated carbon (AC) carrier was successively added, refluxing at 60 °C for 8 h, and the solvent was removed. After that, the obtained material was pyrolyzed at 800 °C under a steady flow rate of argon. Then, the non-supported cobalt particles generated in the process of pyrolysis were etched by HCl. The prepared sample is named Co-N-Si/AC with a Co content of 1.3 wt% (measured by ICP-OES analysis). In addition, for comparison purposes, the samples obtained without the addition of Si precursor, metal source or 1,10-phenanthroline during the preparing process were donated as Co-N/AC, N-Si/AC and Co-Si/AC, respectively.

The catalyst materials obtained were then used for the oxidative esterification of benzyl alcohol **1a** with CH_3_OH **2a** to give methyl benzoate **3aa**. By performing the reaction at room temperature in the presence of molecular O_2_ as the sole oxidant and catalytic amounts of K_2_CO_3_, we investigated the catalytic activity of Co-N-Si/AC without pyrolysis, Co-N/AC, N-Si/AC, Co-Si/AC, Co(OAc)_2_·4H_2_O and Co-N-Si/AC on the reaction (Table 1, entries 1–6). Only Co-N-Si/AC exhibited excellent catalytic performance for the formation of **3aa** (entry 6, 96%), indicating that the doped N and Si elements, and the Co particle as well as the pyrolysis process are essential to leading to an active catalyst. As expected, no product formation was found without the use of any catalyst or base (entries 7–8). Interestingly, adding N species into the catalyst without Si showed a 69% yield of ester (Table 1, entry 2). Using the Co-N-Si/AC material with or without a base gave 97% yield and 0% yield of ester, respectively (Table 1, entries 6 and 7). The test results showed that either AC, N/AC, Si/AC or Co/AC alone, or a physical mixture of Co, Si, C and N without pyrolyzing, were totally inactive (Table 1, entry 8 and Table S1, entries 1–4). All of these results showed that the existence of Co-N species and the base is critical to obtaining a satisfactory yield and that Co-N species originated from high temperature treatment. Furthermore, the change in metal sources showed no catalytic activity (Table 1, entries 9–12). Therefore, the optimal reaction system is as shown in entry 6 of Table 1.

**Figure 1:**
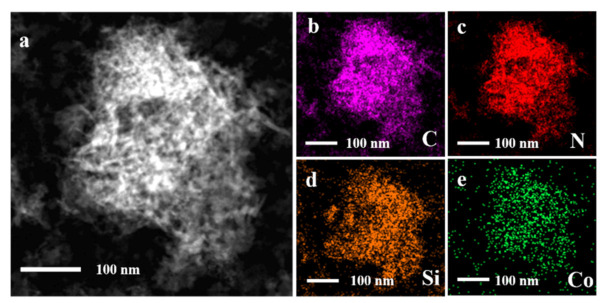
(**a**) TEM image of Co-N-Si/AC and (**b**–**e**) the corresponding elemental mapping of C, N, Si and Co, respectively.

Characterization of the Co-N-Si/AC catalyst by XRD, BET, TEM, XPS, EDX and ICP-OES. The XRD pattern of Co-N-Si/AC (Figure S1) showed no peaks ascribed to the Co metal. Except for two peaks of carbon at 26° and 43°, which are denoted to the (002) and (100) planes, the diffraction pattern had a graphite-like carbon structure [40,41]. While the existence of Co was confirmed by EDX detection (Figure S2), the presence of small distributed Co particles was additionally verified by transmission electron microscopy (TEM) (Figure 1). These results indicated that cobalt particles are highly dispersed with low density and small size [42,43]. TEM images also showed highly regular morphology of Co-N-Si/AC compared with Co-N/AC (Figure S3), although both samples possessed highly dispersed metal particles. The mean Co particle size, as measured by TEM, is 0.6 nm. The Co particle size distribution is shown in Figure S4. In addition, the element mapping revealed the uniform distributions of Co, N, Si and C (Figure 1), which is in accordance with the results of the EDX (Figure S2).

The specific surface area calculated by the Brunauer–Emmett–Teller (BET) (Table S2) method was found to be 485.5 m^2^g^−1^. The calculated pore-size distribution is shown; Co-N-Si/AC mainly contained mesopores with a total pore volume of 0.51 cm^3^g^−1^, and an average pore width of 4.2 nm. Meanwhile, Co-N/AC mainly possessed micropores with a total pore volume of 0.36 cm^3^g^−1^ and a mean pore width of 1.8 nm. The differences of pore volume and width may be the reason for their different catalytic activities. These results indicated that the introduction of Si modified the structure of catalytic material, and the structure of the catalyst played a very important role in this reaction. Inductively coupled plasma optical emission spectrometry (ICP-OES) measurements revealed 1.3 wt% cobalt in the Co-N-Si/AC material.

**Figure 2:**
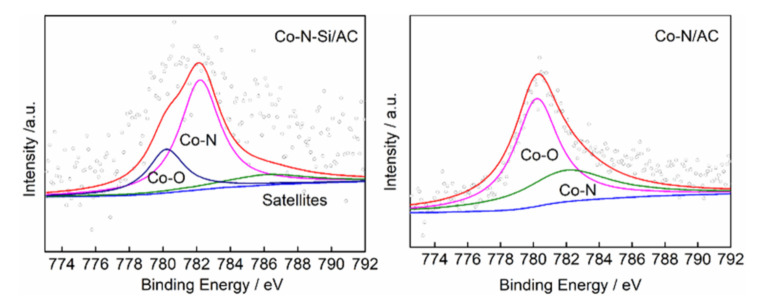
XPS spectra of Co2p in Co-N-Si/AC (**left**) and Co-N/AC (**right**).

The X-ray photoelectron spectroscopy (XPS) analysis showed three distinct peaks in the Co2p spectra of Co-N-Si/AC with electron binding energies of 780.2, 782.0 and 787.0 eV, which were assigned to Co-O, Co-N and satellites, respectively [44,45,46]. Additionally, the content of Co-N was up to 63.2% and that of Co-O was 23.2%, while only 39.1% of Co-N and 60.9% of Co-O were found in the material Co-N/AC (Figure 2 and Table 2). This means that the Co-N serves as an active catalytic site ingredient in our case (Table 1, entries 5 and 6). In the N region, three distinct peaks with electron binding energies of 398.9 (pyridine-type), 400.7 (pyrrole-type) and 402.2 eV (ammonia N) were found (Figure S5 and Table S3) [38]. The pyridine-type nitrogen in this case is bound to cobalt [47], while the pyrrole-type nitrogen originates from the calcination of 1,10-phenanthroline. Deconvolution showed that around 36.2% of the N atoms are bonded to the cobalt metal (see Table S3, Co-N), derived from the graphitization of the Co-Phen complex [48] and serving as the active catalytic site ingredient.

**Scheme 2:**
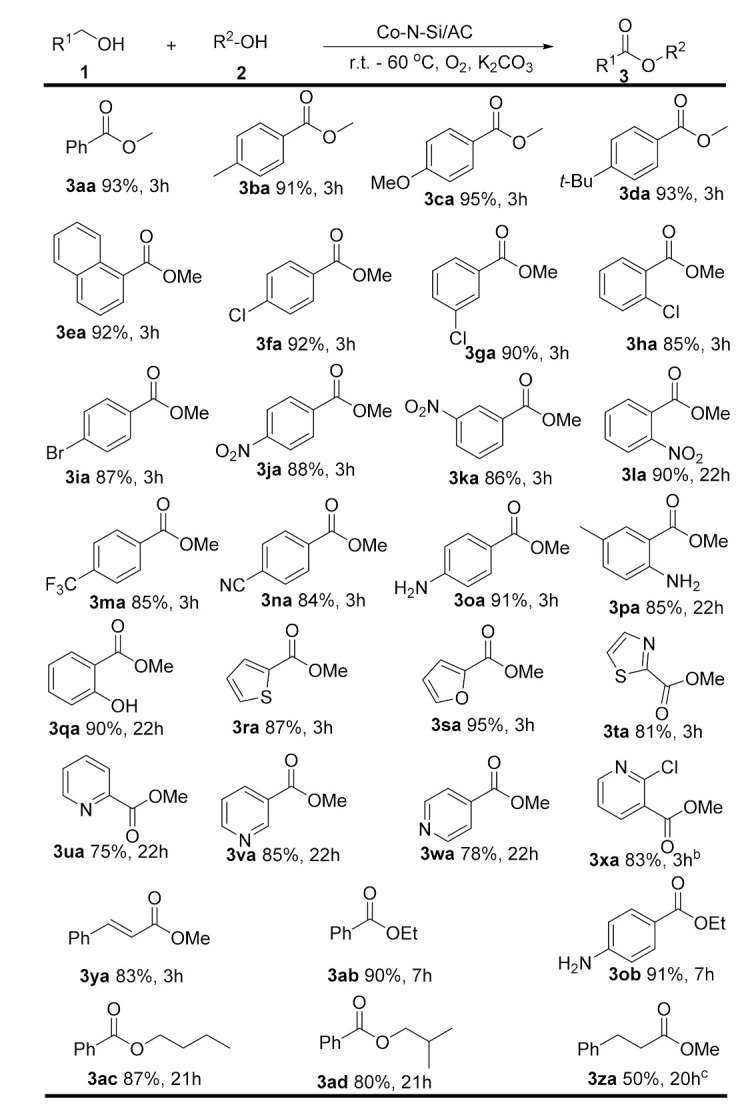
Co-N-Si/AC-catalyzed oxidative esterification of alcohols ^a^. ^a^ Reaction conditions: **1** (0.5 mmol), **2** (1.5 mL), catalyst (40 mg, 1.75 mol%), 0.1 mmol K_2_CO_3,_ at room temperature for 3–22 h under O_2;_ ^b^ 60 °C; ^c^ 80 °C.

After demonstrating the excellent activity of the Co-N-Si/AC catalyst in the model reaction, a series of structurally diverse benzylic and heterocyclic alcohols were chosen to investigate the general applicability of the synthetic protocol under the optimal conditions. The alcohols with both electron-donating groups and electron-withdrawing groups were efficiently converted into corresponding methyl esters. As we can see from Table 1, the desired esters were obtained in 91–95% yields when benzylic alcohols substituted with strongly electron-donating groups such as t-Bu were employed (Scheme 2, **3ba**–**3ea**). The different substituted positions on the phenyl ring had an influence on the catalytic activity of the reaction. As an example, the Cl groups at the meta and ortho positions showed slightly lower activities than that in the para position (**3fa**, **3ga** and **3ha**). When related to the ortho position, prolonging the reaction time was needed to obtain a high yield due to the steric hindrance (**3la**, **3pa** and **3qa**). Apart from the electron-donating groups, benzylic alcohols substituted with electron-withdrawing groups, such as Cl, Br, NO_2_, CF_3_, CN, NH_2_ and OH, all smoothly gave the corresponding esters in >83% yields (**3fa**–**3qa**). Notably, even more sensitive allylic alcohols, such cinnamic alcohol, gave the desired ester in 83% yield (**3ya**). Even heterocyclic alcohols, including 2-thiophenemethanol; 2-furanemethanol; thiazol-2-methanol; and 2-, 3-, 4-pyridine-methanol, could be converted over Co-N-Si/AC to their corresponding esters in up to 95% yield (**3ra**–**3xa**). After successful application of the oxidative esterification of benzyl alcohol with methanol, we tried to extend the catalytic system to other aliphatic alcohols, including ethanol, propanol, butanol and isobutyl alcohol, which also proceeds smoothly, further expanding the scope of the synthesis of esters from methyl to other alkyl esters (**3ab**–**3ad**). Additionally, cross-oxidative esterification of phenylpropanol and methanol could be optimized using a longer reaction time and a slightly higher reaction temperature (**3za**).

All of these experiments indicated that Co-N-Si/AC has good functional group tolerance and high catalytical activity for the oxidative esterification of alcohols using molecular oxygen under mild conditions.

**Scheme 3:**
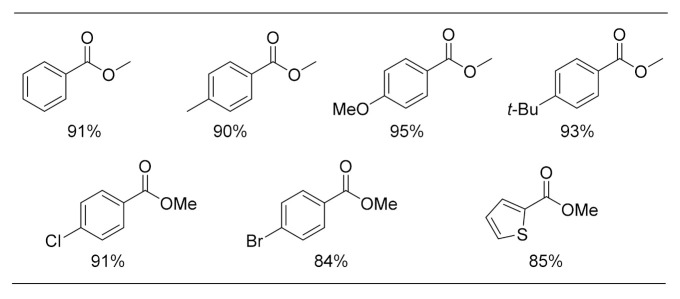
Reaction conditions: **1** (5 g), **2** (100 mL), catalyst (1.75 mol%), 0.2 equiv K_2_CO_3,_ at room temperature for 3 h.

In order to further verify the practicality of the reaction, seven of the substrates were selected, and the amount of the substrates was expanded to 5 g and reacted with methanol. As shown in Scheme 3, the yield of the corresponding products of all these substrates is up to 95%.

**Figure 3:**
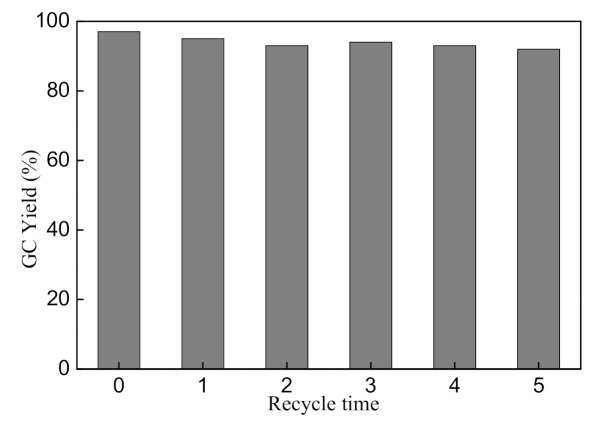
The reusability of the Co-N-Si/AC catalyst.

To demonstrate the stability and reusability of the prepared catalyst material, it was recycled and reused for five consecutive runs with the model reaction. Gratifyingly, the Co-N-Si/AC was successfully recycled without any obvious loss of catalytic activity (Figure 3). Only a slight decrease in Co content from 1.3 wt% to 1.12 wt% can be found (determined by ICP-OES analysis). After recycling, a slight decrease in specific surface area, pore volume and width of the Co-N-Si/AC (used) was found but still belongs to mesopores and causes no significant decrease in catalytic performance (Table S2).

**Scheme 4:**
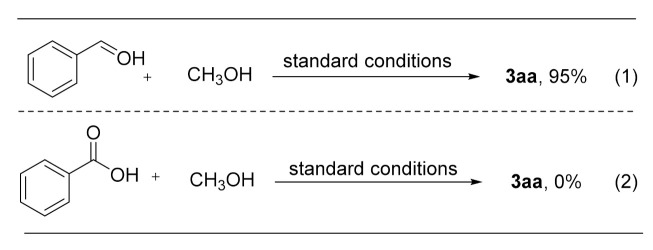
Control experiments.

**Scheme 5:**
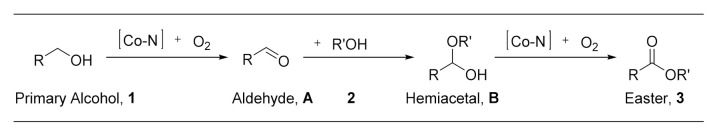
Possible reaction pathway.

To gain more information about the reaction, some control experiments were performed under various conditions. When benzaldehyde was used as a substrate to react with methanol under standard conditions, 95% of the product **3aa** could be obtained. However, when benzaldehyde was replaced with benzoic acid to react with methanol under standard conditions, the product **3aa** was not obtained (Scheme 4). On the basis of the above findings and relative reported mechanism, the plausible reaction pathway for the aerobic oxidation of benzyl alcohols to esters is proposed in Scheme 5 [49]. First, the primary alcohol **1** is oxidized to aldehyde **A** under standard reaction conditions, then reacted with aliphatic alcohol **2** to form intermediate **B** and further oxidized under standard reaction conditions to form target product **3**.

## 3. Conclusions

In conclusion, a stable, earth-abundant, reusable Co catalyst was developed and used to catalyze the oxidative esterification of alcohols under ambient conditions. The advantages offered by this general procedure are operational simplicity, applicability to oxidative esterification of alcohols featuring broad substrate scope and good to high yields for the corresponding products. This protocol enables easy recyclability of the catalyst, measured up to five times without significant loss of efficiency. In general, the process is simple, cost-effective and environmentally benign.

## 4. Experimental

### 4.1. General Information

All of the products obtained were characterized by melting points (m.p), ^1^H-NMR, ^13^C-NMR and infrared spectra (IR). The melting points were measured on an Electrothermal SGW-X4 microscopy digital melting point apparatus and were uncorrected; the IR spectra were recorded on a FTLA2000 spectrometer; and the ^1^H-NMR and ^13^C-NMR spectra were obtained on Bruker-400 and referenced to 7.27 ppm for chloroform solvent with TMS as an internal standard (0 ppm). Chemical shifts were reported in parts per million (ppm, δ) downfield from tetramethylsilane. Proton coupling patterns are described as singlet (s), doublet (d), triplet (t) or multiplet (m); TLC was performed using commercially prepared 100–400 mesh silica gel plates (GF254), and visualization was effected at 254 nm. Unless otherwise stated, all of the reagents were purchased from commercial sources (J&KChemic, TCI, Fluka, Acros, SCRC) and used without further purification.

X-ray diffraction (XRD) was used for crystal structure identification and performed with a Bruker D8 advanced X-ray diffractometer. Micromeritics ASAP 2020 was used to the measure surface area and pore structure (BET) by N_2_ adsorption. Transmission electron microscopy (TEM) and Energy Dispersive X-ray spectroscopy (EDX) were used to observe the morphology of samples, using a Tecnai-G20. The atomic emission spectrometry (ICP) was used to analyze metal content in the samples. The electronic states were measured by X-ray photoelectron spectroscopy (XPS) using a K-Alpha spectrometer with a monochromatized Al-Kα X-ray source (300 W).

### 4.2. Catalyst Preparation

Initially, Co(OAc)_2_·4H_2_O and 1,10-phenanthroline were added into ethanol (50 mL) and stirred for 2 h at 60 °C, aiming to generate a Co complex in situ. Then, Si(OC_2_H_5_)_4_ was introduced into the above solution, followed by hydrolysis of TEOS with aqueous ammonia to generate silicon dioxide in situ, refluxed for another 2 h. After that, an activated carbon carrier was put into the solvent and refluxed for 8 h at 60 °C. Then, the solvent of the suspension was removed under vacuum, and the remaining solid was dried overnight. Then, the powder sample was pyrolyzed at 800 °C under a constant argon atmosphere for 2 h and cooled to room temperature. It was subsequently treated with HCl solution to selectively remove the unsupported cobalt particles generated during the pyrolysis process, named Co-N-Si/AC (the Co content was 1.3 wt%, which was determined by ICP-OES measurements). Samples obtained without the addition of the Si precursor, metal source or 1,10-phenanthroline during the preparation process were denoted as Co-N/AC, N-Si/AC and Co-Si/AC, respectively. In contrast, samples obtained with different metal sources were denoted as Metal-N-Si/AC.

### 4.3. Typical Procedure for the Synthesis of Ester ***3aa***

Benzyl alcohol (0.5 mmol), 1.5 mL methanol, K_2_CO_3_ (0.1 mmol) and forty milligrams of the catalysts (Co-N-Si/AC, 1.75 mol% Co) were added into a 25 mL Schlenk tube and then stirred at room temperature for 3 h under O_2_ atmosphere. The resulting mixture was filtered and washed by ethyl acetate and then concentrated by removing the solvent under vacuum. Finally, the residue was purified by preparative TLC on silica, eluting with petroleum ether (60–90 °C): ethyl acete (25:1) to give the product.

## Figures and Tables

**Table 1:**
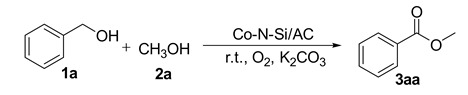
Optimization of reaction conditions ^a^.

Entry	Catalyst	t (h)	Yield (%) ^b^
1	Co-N-Si/AC	3	0 ^c^
2	Co-N/AC	3	69
3	N-Si/AC	3	0
3	Co-Si/AC	3	0
5	Co(OAc)_2_·4H_2_O	3	trace
6	Co-N-Si/AC	3	97
7	-	3	0
8	Co-N-Si/AC	3	0 ^d^
9	Cu-N-Si/AC	3	0
10	Mn-N-Si/AC	3	0
11	Pd-N-Si/AC	3	0
12	Fe-N-Si/AC	3	0

^a^ Conditions: **1a** (0.5 mmol), K_2_CO_3_ (0.1 mmol), catalyst (40 mg, Co: 1.75 mol%), CH_3_OH (1.5 mL) under oxygen, room temperature, 3 h. ^b^ GC yield with hexadecane as an internal standard. ^c^ Catalyst without pyrolysis. ^d^ Without the base.

**Table 2:** The binding energy and content of Co in the catalysts.

Catalyst	Binding Energy/eV (Area/%)		
	Co-O	Co-N	Satellites
Co-N/AC	780.2	782.0	-
	(60.9)	(39.1)	
Co-N-Si/AC	780.0	782.2	787.0
	(23.2)	(63.2)	(13.6)

## Data Availability

Not applicable.

## Supplementary Materials

The following are available online, Figure S1: XRD pattern of Co-N-Si/AC, Figure S2: EDX analysis of the Co-N-Si/AC catalyst, Figure S3: TEM images of Co-N/AC, Figure S4: Particle size distribution of Co-N-Si/AC, Figure S5: XPS spectra of N1s in Co-N-Si/AC, Table S1: Screening of optimal conditions for the synthesis of methyl benzoate, Table S2: Pore structure of the catalysts, Table S3: The binding energy and content of N in the catalyst, Table S4: Substrates employed for oxidative esterification.

## Sample Availability

Samples of the compounds are not available from the authors.
