# Supplementary material for: Co-N-Si/AC Catalyst for Aerobic Oxidation of Benzyl Alcohols to Esters under Mild Conditions
Source: Molecules. 2021 Nov 10;26(22):6792. doi: 10.3390/molecules26226792 (PMC8622685; doi:10.3390/molecules26226792)

# Co-N-Si/AC catalyst for aerobic oxidation of alcohols to esters under mild conditions

Changjian Zhou [a], Rong Sun [a], Yuting Zhang [c], Biao Xiong [c], Hui Dai [b]*, Yong Dai [a]*

[a] School of Chemistry and Chemical Engineering, Yancheng Institute of Technology, Yancheng, Jiangsu Province, 224051, China.

[b] College of Materials and Chemistry & Chemical Engineering, Chengdu University of Technology, Chengdu, 610059, China

[c] school of pharmacy, Nantong University, 19 Qixiu Road, Nantong, Jiangsu Province 226001, China

*Corresponding emails: daihui18@cdut.edu.cn, 56776278@qq.com

**Table of contents**

1. General information S2
2. Procedure for the preparation of catalysts S3
3. Typical procedure for the oxidative esterification of benzyl alcohol S3
4. Recycling of catalyst for the oxidative esterification of benzyl alcohol S3
5. Screening of optimal conditions for the synthesis of methyl benzoate S4
6. BET measurement of the catalysts S4
7. Power x-ray diffraction S4
8. TEM measurement of the catalysts S5
9. EDX analysis of the catalyst S5
10. XPS spectra of N1s in the catalyst S6
11. Substrates employed for synthesizing oxidative esterification S7
12. Analytic data of the obtained compounds S8-15
13. References S15
14. $^{1}$H-NMR and $^{13}$C-NMR spectra of the obtained compounds S16-45

## 1. General information

All the obtained products were characterized by melting points (m.p), $^{1}H$-NMR, $^{13}C$-NMR and infrared spectra (IR). Melting points were measured on an Electrothermal SGW-X4 microscopy digital melting point apparatus and are uncorrected; IR spectra were recorded on a FTLA2000 spectrometer; $^{1}H$-NMR and $^{13}C$-NMR spectra were obtained on Bruker-400 and referenced to 7.27 ppm for chloroform solvent with TMS as internal standard (0 ppm). Chemical shifts were reported in parts per million (ppm, δ) downfield from tetramethylsilane. Proton coupling patterns are described as singlet (s), doublet (d), triplet (t), multiplet (m); TLC was performed using commercially prepared 100-400 mesh silica gel plates (GF254), and visualization was effected at 254 nm; Unless otherwise stated, all the reagents were purchased from commercial sources (*J & K* Chemicals, TCI, Fluka, Acros, SCRC), used without further purification.

X-ray diffraction (XRD) was used for crystal structure identification as used by a Bruker D8 advanced X-ray diffractometer. Micromeritics ASAP 2020 used to measure the specific surface area and pore structure (BET) by $N_2$ adsorption. Transmission electron microscopy (TEM) and Energy Dispersive X-ray spectroscopy (EDX) using a Tecnai-G20 to observed the morphology of samples. The atomic emission spectrometry (ICP) was used to analyze the metal content in the samples. The electronic states were measured by X-ray photoelectron spectroscopy (XPS) using an K-Alpha spectrometer with a monochromatized Al-Kα X-ray source (300W).

**2. Procedure for the preparation of catalysts**

The mixture of $Co(OAc)_2·4H_2O$ (500 mg, 2.0 mmol) and 1,10-phenanthroline (720 mg, 4 mmol) (Co : phenanthroline = 1 : 2 molar ratio) was added to ethanol (100 mL) and stirred at 60 °C for 2 hours. Silica was then introduced into the above solution by *in situ* hydrolysis of the added $Si(OC_2H_5)_4$ (TEOS) with aqueous ammonia. After that, the commercially available powder activated carbon (2500 mg) as the support was added to the solution and refluxed for 8 h at 60 °C, then the solvent of the suspension was removed and the remained solid was dried overnight at 60 °C under vacuum. Then, the sample was grounded to a fine powder and then pyrolyzed at 800 °C under a constant argon flow for 2 hours. After cooling down to room temperature, the catalyst material was finally afforded by treating the sample with HCl solution to remove non-supported cobalt particles, which is named as Co-N-Si/AC (the Co content is 1.3 wt %, which is determined by ICP-OES measurements). Similarly, the materials prepared in absence of TEOS, 1,10-phenanthroline and metal source are donated as Co-N/AC, Co-Si/AC and N-Si/AC, respectively. And the catalysts prapared with different metal sources are denoted as Metal-N-Si/AC, respectively.

**3. Typical procedure for the oxidative esterification of benzyl alcohol**

The mixture of benzyl alcohol (0.5 mmol), 1.5 mL methanol, $K_2CO_3$ (0.1 mmol) and forty milligrams of the catalyst (Co-N-Si/AC, 1.75 mol % Co) was added into a 25 mL schlenk tube, then stirred at room temperature for 3 h under $O_2$ atmosphere. After that, the resulting mixture was filtered and washed with ethyl acetate, and then concentrated by removing the solvent under vacuum. Finally the residue was purified by preparative TLC on silica, eluting with petroleum ether (60 - 90 °C) : ethyl acetate (25 : 1, v/v) to give the methyl benzoate.

**4. Recycling reaction of catalyst for the oxidative esterification of benzyl alcohol**

The used catalyst was collected by filtration and washed with pure methanol, then dried in the oven under vacuum. The catalyst was then used for the next catalytic reaction.

## 5. Screening of optimal conditions for the synthesis of methyl benzoate

**Table S1** Screening of optimal conditions for the synthesis of methyl benzoate [a]

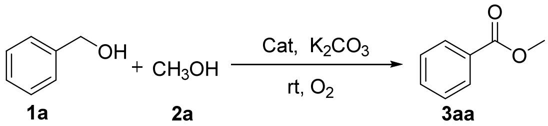


| Entry | Catalyst | Additive | Yield (%)[b] |
|---|---|---|---|
| 1 | AC | $K_2CO_3$ | 0 |
| 2 | N/AC | $K_2CO_3$ | 0 |
| 3 | Si/AC | $K_2CO_3$ | 0 |
| 4 | Co/AC | $K_2CO_3$ | 0 |

[a]Reaction conditions: **1a** (0.5 mmol), **2a** (1.5 mL), catalyst (1.75 mol %, 40 mg), $K_2CO_3$ (0.1 mmol) were stirred at room temperature for 3 h under $O_2$. [b]GC yield by using hexadecane as an internal standard.

## 6. BET measurement of the catalysts

**Table S2** Pore structure of the catalysts.

| Samples | D (nm) | $S_{BET}$ ($m^2g^{-1}$) | V ($cm^3g^{-1}$) |
|---|---|---|---|
| Co-N-Si/AC | 4.2 | 485.5 | 0.51 |
| Co-N/AC | 1.8 | 594.3 | 0.36 |
| Co-N-Si/AC (Used) | 3.7 | 415.6 | 0.42 |

## 7. Power x-ray diffraction

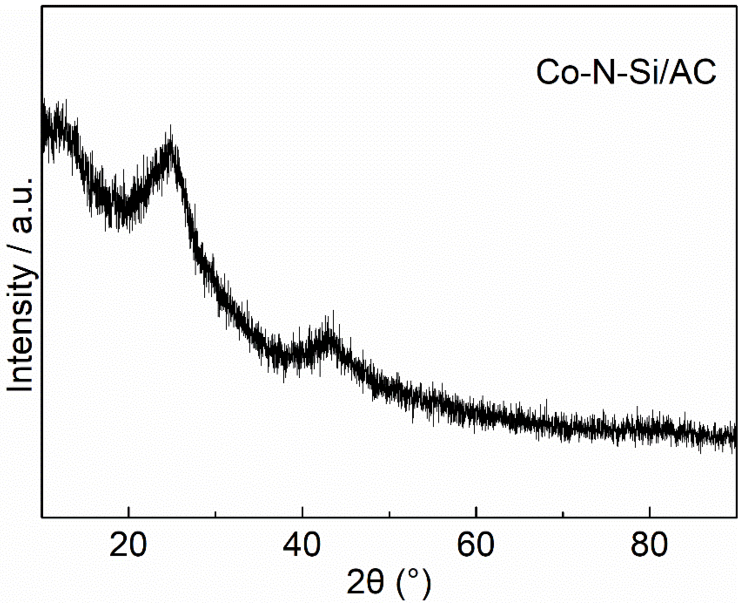


**Figure S1** XRD pattern of Co-N-Si/AC.

## 8. EDX analysis of the catalyst

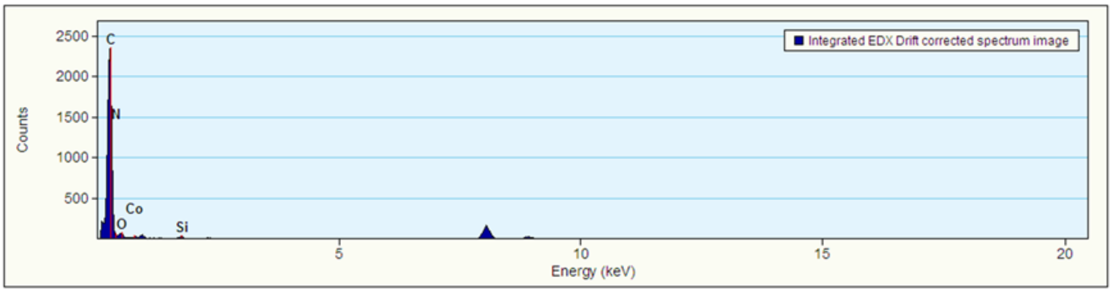


**Figure S2** EDX analysis of the Co-N-Si/AC catalyst.

## 9. TEM measurement of the catalysts

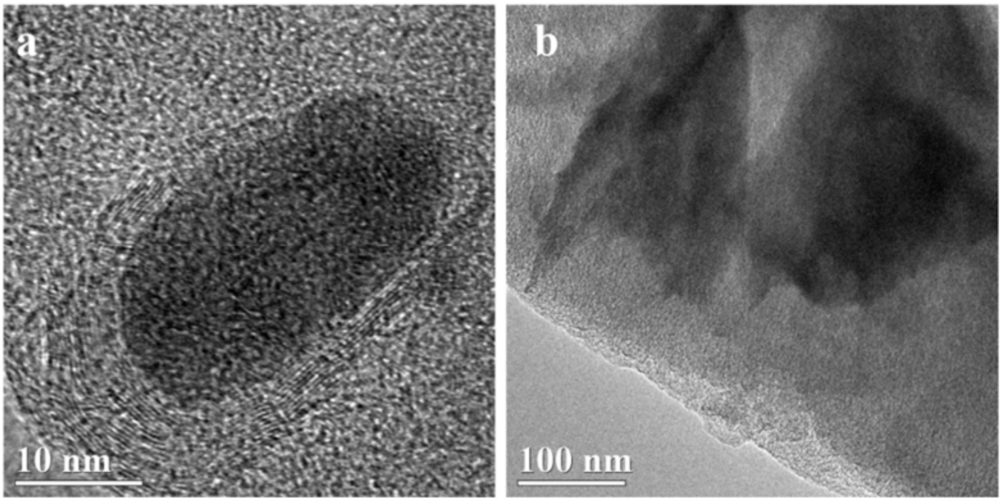


**Figure S3** TEM images of Co-N/AC.

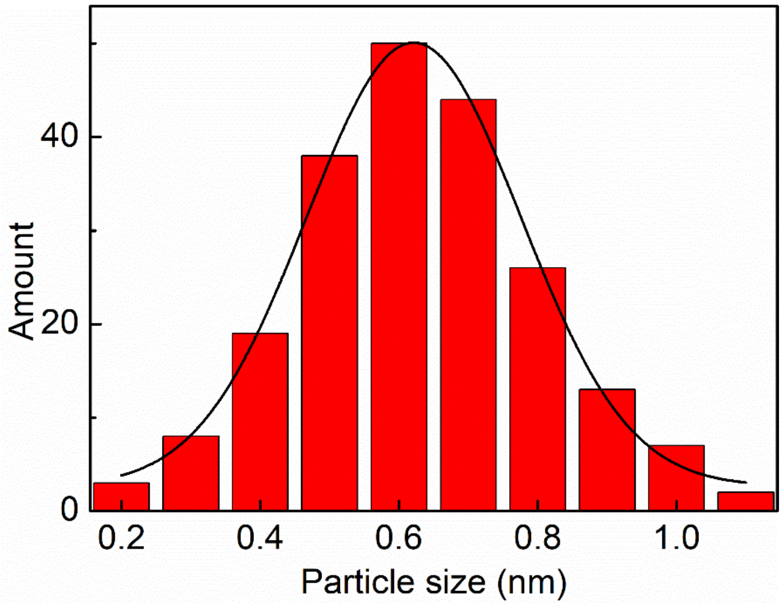


**Figure S4** Particle size distribution of Co-N-Si/AC.

## 10. XPS spectra of N1s in the catalyst

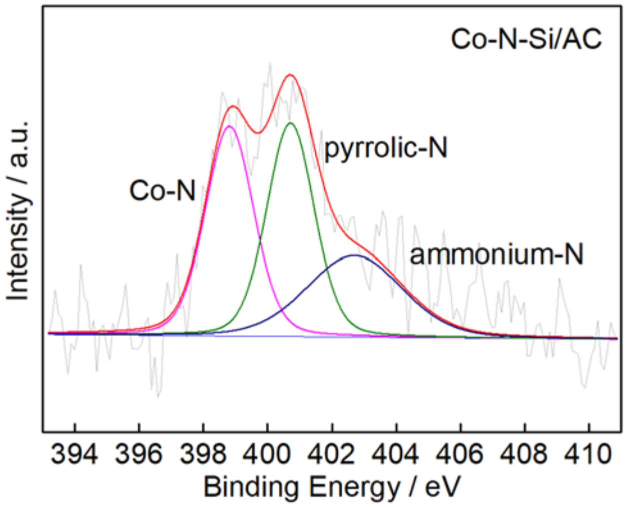


**Figure S5** XPS spectra of N1s in Co-N-Si/AC.

**Table S3.** The binding energy and content of N in the catalyst

| Sample | Binding Energy / eV (Area/%) | | |
|---|---|---|---|
| | Co-N | Pyrrolic-N | Ammonia-N |
| Co-N-Si/AC | 398.8<br>(36.2) | 400.7<br>(36.0) | 402.6<br>(27.6) |

## 11. Substrates employed for synthesizing oxidative esterification

**Table S4.** Substrates employed for oxidative esterification

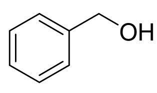

**1a**

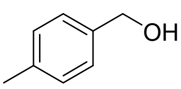

**1b**

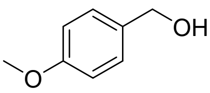

**1c**

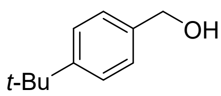

**1d**

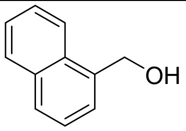

**1e**

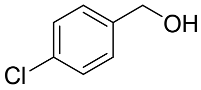

**1f**

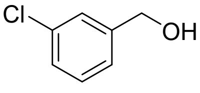

**1g**

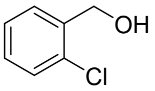

**1h**

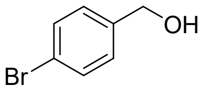

**1i**

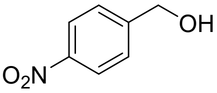

**1j**

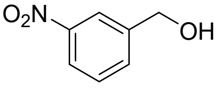

**1k**

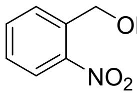

**1l**

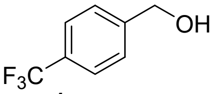

**1m**

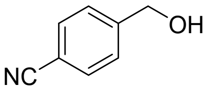

**1n**

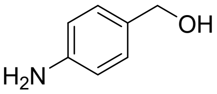

**1o**

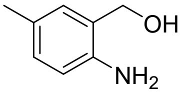

**1p**

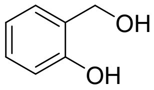

**1q**

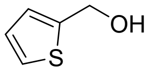

**1r**

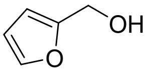

**1s**

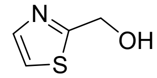

**1t**

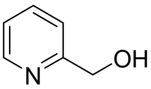

**1u**

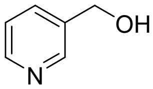

**1v**

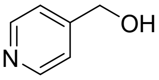

**1w**

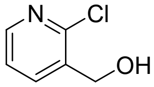

**1x**

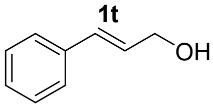

**1y**

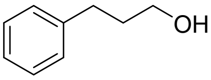

**1z**

## 12. Analytic data of the obtained compounds

**methyl benzoate (3aa)[1]**

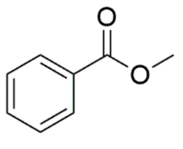

Colorless liquid (63 mg, 93% yield); $^{1}$H NMR (400 MHz, $CDCl_3$): δ 8.04 (dd, *J* = 8.4, 1.2 Hz, 2H), 7.55 (d, *J* = 7.6 Hz, 1H), 7.43 (d, *J* = 7.6 Hz, 2H), 3.92 (s, 3H). $^{13}$C NMR (101 MHz, $CDCl_3$) δ 167.24, 133.02, 130.31, 129.70, 128.47, 52.20. IR (KBr): 2922, 2362, 2335, 1836, 1740, 1694, 1539, 1516, 1463, 1265, 1018, 751 cm$^{-1}$. MS (EI, m/z): 136 $[M]^+$.

**methyl 4-methylbenzoate (3ba)[1]**

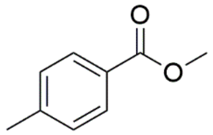

Colorless oil (68 mg, 91% yield); $^{1}$H NMR (400 MHz, $CDCl_3$): δ 7.84 (d, *J* = 8.0 Hz, 2H), 7.14 (d, *J* = 8.4 Hz, 2H), 3.80 (s, 3H), 2.31 (s, 3H). $^{13}$C NMR (101 MHz, $CDCl_3$): δ 167.24, 143.61, 129.68, 129.14, 127.54, 51.98, 21.69. IR (KBr): 3067, 2361, 2334, 1723, 1515, 1279, 1181, 1107, 1021, 753, 674 cm$^{-1}$. MS (EI, m/z): 150 $[M]^+$.

**methyl 4-methoxybenzoate (3ca)[1]**

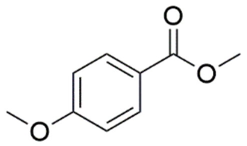

Colorless oil (79 mg, 95% yield); $^{1}$H NMR (400 MHz, $CDCl_3$): δ 7.89 (d, *J* = 8.8 Hz, 2H), 7.80 (d, *J* = 8.8 Hz, 2H), 3.78 (s, 3H), 3.74 (s, 3H). $^{13}$C NMR (101 MHz, $CDCl_3$): δ 166.85, 163.38, 131.60, 122.64, 113.63, 55.39, 51.82. IR (KBr): 3001, 2958, 2842, 2361, 1922, 1607, 1512, 1436, 1320, 1285, 1259, 1171, 1024, 965, 847, 769, 696 cm$^{-1}$. MS (EI, m/z): 166 $[M]^+$.

**methyl 4-(tert-butyl)benzoate（3da）**

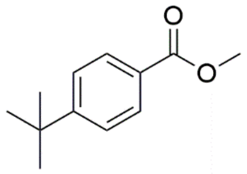

Colorless oil (90 mg, 93% yield); $^{1}$H NMR (400 MHz, $CDCl_3$): δ 7.97 (d, *J* = 8.4 Hz, 2H), 7.44 (t, *J* = 8.4 Hz, 2H), 3.89 (s, 3H), 1.33 (s, 9H). $^{13}$C NMR (101 MHz, $CDCl_3$): δ 167.18, 156.58, 129.53, 127.50, 125.39, 51.97, 35.13, 31.19. IR (KBr): 2962, 1725, 1609, 1435, 1409, 1365, 1280, 1189, 1117, 1018, 854, 775, 707 cm$^{-1}$. MS (EI, m/z): 192 $[M]^+$.

**methyl 1-naphthoate (3ea)[2]**

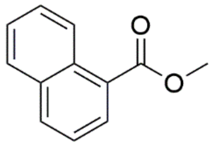

White solid (78 mg, 92% yield), m.p: 61-62 °C; $^1$H NMR (400 MHz, $CDCl_3$): δ 8.15 (dd, *J* = 7.6, 1.2 Hz, 1H), 7.97 (d, *J* = 8.0 Hz, 1H), 7.84 (d, *J* = 7.6 Hz, 1H), 7.57-7.61 (m, 1H), 7.43-7.51 (m, 2H), 3.97 (s, 3H). $^{13}$C NMR (101 MHz, $CDCl_3$): δ 168.07, 133.92, 133.42, 131.42, 130.29, 128.60, 127.82, 127.15, 126.26, 125.90, 124.54, 52.17. IR (KBr): 3053, 2950, 2843, 2362, 1959, 1740, 1584, 1510, 1437, 1395, 1280, 1246, 1200, 1136, 1017, 950, 849, 812, 781 $cm^{-1}$. MS (EI, m/z): 186 $[M]^+$.

**methyl 4-chlorobenzoate (3fa)[1]**

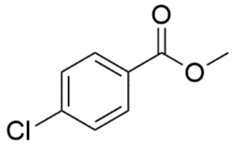

Colorless solid (78 mg, 92% yield), m.p: 42-43 °C; $^1$H NMR (400 MHz, $CDCl_3$): δ 7.89 (d, *J* = 8.8 Hz, 2H), 7.32 (d, *J* = 8.8 Hz, 2H), 3.83 (s, 3H). $^{13}$C NMR (101 MHz, $CDCl_3$): δ 166.32, 139.48, 131.08, 128.82, 128.73, 52.36. IR (KBr): 2956, 2361, 2335, 1918, 1726, 1645, 1597, 1280, 1095, 1015, 759, 679 $cm^{-1}$. MS (EI, m/z): 170 $[M]^+$.

**methyl 3-chlorobenzoate (3ga)[1]**

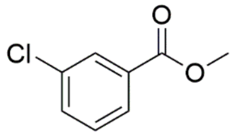

Colorless oil (77 mg, 90% yield); $^1$H NMR (400 MHz, CDCl3): δ 8.01 (s, 1H), 7.91 (d, *J* = 8.0 Hz, 1H), 7.52 (d, *J* = 8.8 Hz, 1H), 7.37 (t, *J* = 8.0 Hz, 1H), 3.92 (s, 3H). $^{13}$C NMR (101 MHz, $CDCl_3$): δ 165.93, 134.61, 133.02, 131.98, 129.76, 127.78, 52.46. IR (KBr): 3072, 3000, 2953, 2361, 1574, 1434, 1287, 1259, 1126, 1080, 973, 753, 675 $cm^{-1}$. MS (EI, m/z): 170 $[M]^+$.

**methyl 2-chlorobenzoate (3ha)**

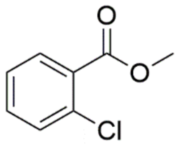

Colorless oil (72 mg, 85 % yield); $^1$H NMR (400 MHz, $CDCl_3$): δ 7.73 (dd, *J* = 8.0, 1.6 Hz, 1H), 7.30-7.37 (m, 2H), 7.20-7.24 (m, 1H), 3.84 (s, 3H). $^{13}$C NMR (101 MHz, $CDCl_3$): δ 166.23, 133.77, 132.62, 131.46, 131.14, 130.18, 126.64, 52.49. IR (KBr): 3070, 2846, 2362, 1592, 1471, 1295, 1253, 1190, 1121, 1049, 960, 793, 750 $cm^{-1}$. MS

(EI, m/z): 170 $[M]^+$.

**methyl 4-bromobenzoate (3ia)[1]**

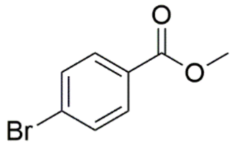

Pale yellow solid (94 mg, 87% yield), m.p: 78-79 °C; $^1$H NMR (400 MHz, $CDCl_3$): δ 7.80 (d, *J* = 8.8 Hz, 2H), 7.48 (d, *J* = 8.4 Hz, 2H), 3.82 (s, 3H). $^{13}$C NMR (101 MHz, $CDCl_3$): δ 166.38, 131.77, 131.18, 129.13, 128.09, 52.34. IR (KBr): 2997, 2362, 2335, 1771, 1717, 1515, 1448, 1394, 1280, 1109, 847, 755, 680 $cm^{-1}$. MS (EI, m/z): 215 $[M]^+$.

**methyl 4-nitrobenzoate (3ja)[1]**

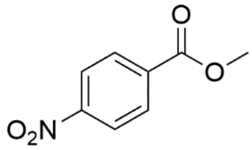

White solid (79 mg, 88% yield), 95-96 °C; $^1$H NMR (400 MHz, $CDCl_3$): δ 8.29 (d, *J* = 8.8 Hz, 2H), 8.21 (d, *J* = 9.2 Hz, 2H), 3.99 (s, 3H). $^{13}$C NMR (101 MHz, $CDCl_3$): δ 165.28, 150.68, 135.61, 130.82, 123.65, 52.94. IR (KBr): 3080, 3011, 2852, 2362, 1800, 1603, 1523, 1435, 1347, 1320, 1284, 1103, 953, 870, 817, 784, 715 $cm^{-1}$. MS (EI, m/z): 181 $[M]^+$.

**methyl 3-nitrobenzoate (3ka)[3]**

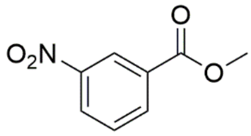

White solid (77 mg, 86% yield), m.p: 77-78 °C; $^1$H NMR (400 MHz, $CDCl_3$): δ 8.85 (s, 1H), 8.42 (d, *J* = 8.8 Hz, 1H), 8.37 (d, *J* = 8.0 Hz, 1H), 7.68 (t, *J* = 8.0 Hz, 1H), 4.00 (s, 3H). $^{13}$C NMR (101 MHz, $CDCl_3$): δ 164.98, 148.34, 135.31, 131.94, 129.72, 127.42, 124.61, 52.84. IR (KBr): 3094, 3013, 2958, 2362, 1842, 1721, 1614, 1523, 1440, 1347, 1293, 1264, 1131, 971, 922, 821, 777 $cm^{-1}$. MS (EI, m/z): 181 $[M]^+$.

**methyl 2-nitrobenzoate (3la)[3]**

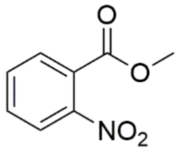

Colorless oil (81 mg, 90 % yield), $^1$H NMR (400 MHz, $CDCl_3$): δ 7.83 (dd, *J* = 7.6, 1.6 Hz, 1H), 7.67 (dd, *J* = 7.6, 1.6 Hz, 1H), 7.54-7.62 (m, 2H), 3.84 (s, 3H). $^{13}$C NMR (101 MHz, $CDCl_3$): δ 165.83, 148.26, 132.91, 131.79, 129.85, 127.53, 123.90, 53.23. IR

(KBr): 3009, 2956, 2919, 2362, 2335, 1837, 1737, 1532, 1437, 1267, 1191, 1123, 1070, 754 $cm^{-1}$. MS (EI, m/z): 181 $[M]^+$.

**methyl 4-(trifluoromethyl)benzoate (3ma)[4]**

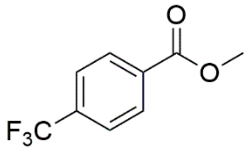

Colorless oil (87 mg, 85% yield); $^1H$ NMR (400 MHz, $CDCl_3$): δ 8.15 (d, $J$ = 8.0 Hz, 2H), 7.71 (d, $J$ = 8.4 Hz, 2H), 3.96 (s, 3H). $^{13}C$ NMR (101 MHz, $CDCl_3$): δ 165.85, 134.44 (d, $J_{C-F}$ = 33.3 Hz), 133.36, 129.97, 125.39 (q, $J_{C-F}$ = 4.0 Hz), 123.63 (d, $J_{C-F}$ = 273.7 Hz). IR (KBr): 2956, 2922, 2851, 2362, 2335, 1918, 1727, 1652, 1515, 1324, 1277, 1169, 1127, 1016, 966, 856, 739 $cm^{-1}$. MS (EI, m/z): 204 $[M]^+$.

**methyl 4-cyanobenzoate (3na)[3]**

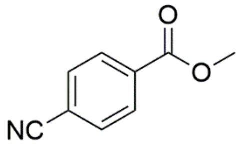

White solid (67 mg, 84% yield), m.p: 65-66 °C; $^1H$ NMR (400 MHz, $CDCl_3$): δ 8.14 (d, $J$ = 8.0 Hz, 2H), 7.75 (d, $J$ = 8.0 Hz, 2H), 3.97 (s, 3H). $^{13}C$ NMR (101 MHz, $CDCl_3$): δ 165.43, 133.98, 132.27, 130.13, 117.97, 116.44, 52.77. IR (KBr): 3104, 3072, 3006, 2956, 2849, 2362, 2334, 2229, 1949, 1747, 1565, 1440, 1404, 1285, 1189, 1110, 961, 865, 764, 690 $cm^{-1}$. MS (EI, m/z): 161 $[M]^+$.

**methyl 4-aminobenzoate (3oa)**

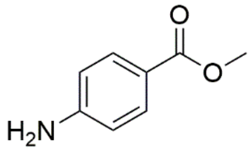

Yellow solid (69 mg, 91% yield); $^1H$ NMR (400 MHz, Methanol-$d_4$): δ 7.75 (d, $J$ = 8.8 Hz, 2H), 6.65 (d, $J$ = 8.8 Hz, 2H), 3.83 (s, 3H). $^{13}C$ NMR (101 MHz, Methanol-$d_4$): δ 169.31, 154.68, 132.46, 118.55, 114.33, 51.96. IR (KBr): 3468, 3372, 2950, 2362, 2335, 1916, 1687, 1597, 1517, 1437, 1289, 1117, 1072, 840, 767, 696 $cm^{-1}$. MS (EI, m/z): 151 $[M]^+$.

**methyl 2-amino-5-methylbenzoate (3pa)**

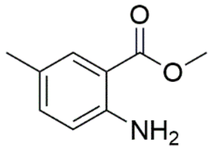

Colorless oil (70 mg, 85% yield); $^1H$ NMR (400 MHz, $CDCl_3$): δ 7.66 (s, 1H), 7.09 (dd, $J$ = 8.0, 2.0 Hz, 1H), 6.59 (d, $J$ = 8.4 Hz, 1H), 3.86 (s, 3H), 2.23 (s, 3H). $^{13}C$ NMR (101

MHz, $CDCl_3$): δ 168.73, 148.36, 135.35, 130.97, 125.58, 116.99, 110.86, 51.59, 20.38. IR (KBr): 2922, 2362, 2335, 1694, 1516, 1462, 1257, 754 $cm^{-1}$. MS (EI, m/z): 165 $[M]^+$.

**methyl 2-hydroxybenzoate (3qa)**

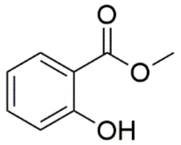

Colorless oil (68 mg, 90% yield); $^1H$ NMR (400 MHz, $CDCl_3$): δ 10.75 (s, 1H), 7.83 (dd, $J$ = 8.0, 1.6 Hz, 1H), 7.45 (t, $J$ = 7.6 Hz, 1H), 6.98 (d, $J$ = 8.8 Hz, 1H), 6.87 (t, $J$ = 7.6 Hz, 1H), 3.95 (s, 3H). $^{13}C$ NMR (101 MHz, $CDCl_3$): δ 170.70, 161.73, 135.82, 130.03, 119.29, 117.71, 112.53, 52.40. IR (KBr): 2925, 2855, 2362, 2335, 1737, 1679, 1535, 1461, 1260, 1023, 800, 754, 674 $cm^{-1}$. MS (EI, m/z): 152 $[M]^+$.

**methyl thiophene-2-carboxylate (3ra)[3]**

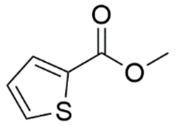

Colorless oil (62 mg, 87% yield); $^1H$ NMR (400 MHz, $CDCl_3$): δ 7.73 (dd, $J$ = 4.0, 1.2 Hz, 1H), 7.48 (dd, $J$ = 5.2, 1.2 Hz, 1H), 7.01-7.04 (m, 1H), 3.82 (s, 3H). $^{13}C$ NMR (101 MHz, $CDCl_3$): δ 162.82, 133.74, 133.58, 132.45, 127.86, 52.26. IR (KBr): 3100, 2962, 2362, 2335, 1740, 1707, 1516, 1464, 1259, 1021, 798, 675 $cm^{-1}$. MS (EI, m/z): 142 $[M]^+$.

**methyl furan-2-carboxylate (3sa)[3]**

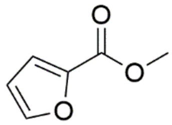

Colorless oil (60mg, 95% yield); $^1H$ NMR (400 MHz, $CDCl_3$): δ 7.58 (s, 1H), 7.18 (d, $J$ = 3.6 Hz, 1H), 6.51-6.52 (m, 1H), 3.90 (s, 3H). $^{13}C$ NMR (101 MHz, $CDCl_3$): δ 159.13, 146.32, 144.63, 117.93, 111.85, 51.87. IR (KBr): 3142, 2955, 1731, 1578, 1477, 1390, 1303, 1196, 1119, 1076, 1015, 968, 911, 884, 761 $cm^{-1}$. MS (EI, m/z): 126 $[M]^+$.

**methyl thiazole-2-carboxylate (3ta)**

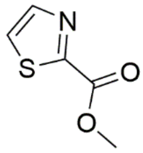

Colorless oil (58 mg, 81 % yield); $^1H$ NMR (400 MHz, $CDCl_3$): δ 8.03 (d, $J$ = 3.2 Hz, 1H), 7.65 (d, $J$ = 2.8 Hz, 1H), 4.03 (s, 3H). $^{13}C$ NMR (101 MHz, $CDCl_3$): δ 160.62, 158.28, 145.10, 125.45, 53.37. IR (KBr): 2993, 2362, 2335, 1767, 1516, 1464, 1372, 1244, 1055, 913, 747 $cm^{-1}$. MS (EI, m/z): 143 $[M]^+$.

**methyl picolinate (3ua)**

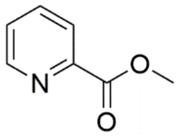

Colorless oil (51 mg, 75% yield); $^1$H NMR (400 MHz, $CDCl_3$): δ 8.76 (d, *J* = 4.4 Hz, 1H), 8.15 (d, *J* = 8.0 Hz, 1H), 7.84-7.88 (m, 1H), 7.47-7.51 (m, 1H), 4.02 (s, 3H). $^{13}$C NMR (101 MHz, $CDCl_3$) δ 165.52, 149.66, 147.80, 136.94, 126.84, 124.99, 52.73. IR (KBr): 2924, 2855, 2362, 2335, 1726, 1516, 1461, 1374, 1311, 1131, 749, 674 cm$^{-1}$. MS (EI, m/z): 137 $[M]^+$.

**methyl nicotinate（3va）**

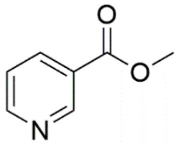

Colorless oil (58 mg, 85% yield); $^1$H NMR (400 MHz, $CDCl_3$): δ 9.23 (d, *J* = 1.6 Hz, 1H), 8.77 (dd, *J* = 4.8, 1.6 Hz, 1H), 8.30 (d, *J* = 8.0 Hz, 1H), 7.40 (dd, *J* = 8.0, 4.8 Hz, 1H), 3.96 (s, 3H). $^{13}$C NMR (101 MHz, $CDCl_3$): δ 165.78, 153.41, 150.91, 137.14, 126.14, 123.37, 52.46. IR (KBr): 2955, 1728, 1590, 1420, 1287, 1192, 1115, 1024, 826, 742, 702 cm$^{-1}$. MS (EI, m/z): 137 $[M]^+$.

**methyl isonicotinate (3wa)[5]**

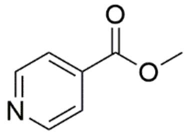

Colorless oil (53 mg, 78% yield); $^1$H NMR (400 MHz, $CDCl_3$): δ 8.78 (d, *J* = 6.0 Hz, 2H), 7.84 (d, *J* = 6.0 Hz, 2H), 3.96 (s, 3H). $^{13}$C NMR (101 MHz, $CDCl_3$): δ 165.78, 153.41, 150.91, 137.14, 126.14, 123.37, 52.46. IR (KBr): 2955, 1728, 1590, 1420, 1287, 1192, 1115, 1024, 826, 742, 702 cm$^{-1}$. MS (EI, m/z): 137 $[M]^+$.

**methyl 2-chloronicotinate (3xa)**

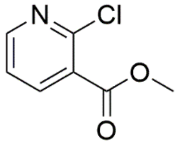

Colorless oil (71 mg, 83% yield);$^1$H NMR (400 MHz, $CDCl_3$): δ 8.52 (dd, *J* = 4.8, 2.0 Hz, 1H), 8.17 (dd, *J* = 7.6, 2.0 Hz, 1H), 7.32-7.35 (m, 1H), 3.97 (s, 3H). $^{13}$C NMR (101 MHz, $CDCl_3$): δ 165.05, 152.03, 150.19, 140.40, 126.93, 122.19, 52.98. IR (KBr): 2955, 2923, 2362, 2335, 1739, 1550, 1455, 1243, 1137, 1058, 955, 913, 746 cm$^{-1}$. MS (EI, m/z): 171 $[M]^+$.

**methyl cinnamate (3ya)**[1]

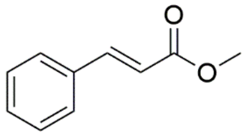

Colorless oil (67 mg, 83% yield); $^1$H NMR (400 MHz, $CDCl_3$): δ 7.69 (d, *J* = 16.0 Hz, 1H), 7.50-7.53 (m, 2H), 7.36-7.39 (m, 3H), 6.44 (d, *J* = 16.0 Hz, 1H), 3.80 (s, 3H). $^{13}$C NMR (101 MHz, $CDCl_3$): δ 167.39, 144.85, 134.41, 130.28, 128.88, 128.06, 117.83, 51.67. IR (KBr): 3061, 3028, 2950, 2361, 1724, 1637, 1443, 1319, 1275, 1199, 1170, 933, 766, 711, 683 cm$^{-1}$. MS (EI, m/z): 162 $[M]^+$.

**ethyl benzoate (3ab)**[3]

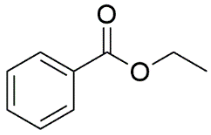

Colorless oil (68 mg, 90% yield); $^1$H NMR (400 MHz, $CDCl_3$): δ 8.05 (d, *J* = 6.8 Hz, 2H), 7.55 (t, *J* = 7.6 Hz, 1H), 7.43 (t, *J* = 7.6 Hz, 2H), 4.38 (q, *J* = 14.4 Hz, 2H), 1.40 (t, *J* = 7.2 Hz, 3H). $^{13}$C NMR (101 MHz, $CDCl_3$): δ 166.73, 132.88, 130.64, 129.63, 128.40, 61.04, 14.43. IR (KBr): 2956, 2335, 1721, 1644, 1515, 1371, 1173, 1106, 1026, 754, 711 cm$^{-1}$. MS (EI, m/z): 150 $[M]^+$.

**ethyl 4-aminobenzoate (3ob)**

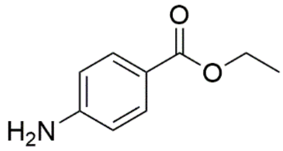


White solid (75 mg, 91% yield), m.p: 91-92 °C; $^1$H NMR (400 MHz, $CDCl_3$): δ 7.85 (d, *J* = 8.4 Hz, 2H), 6.62 (t, *J* = 8.8 Hz, 2H), 4.31 (q, *J* = 7.2 Hz, 2H), 4.09 (s, 2H), 1.35 (t, *J* = 7.2 Hz, 3H). $^{13}$C NMR (101 MHz, $CDCl_3$): δ 166.78, 150.95, 131.75, 119.90, 113.75, 60.30, 14.42. IR (KBr): 3424, 3343, 3224, 3069, 2985, 2956, 2900, 2676, 2362, 1683, 1635, 1600, 1473, 1369, 1309, 1281, 1170, 1123, 1024, 846, 772, 699 cm$^{-1}$. MS (EI, m/z): 165 $[M]^+$.

**butyl benzoate (3ac)**

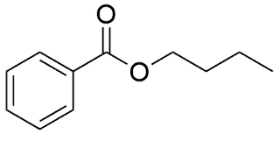

Colorless oil (77 mg, 87% yield); $^1$H NMR (400 MHz, $CDCl_3$): δ 7.96 (d, *J* = 7.2 Hz, 2H), 7.47 (t, *J* = 7.6 Hz, 1H), 7.35 (t, *J* = 7.6 Hz, 2H), 4.25 (t, *J* = 6.8 Hz, 2H), 1.64-1.71 (m, 2H), 1.36-1.45 (m, 2H), 0.91 (t, *J* = 7.2 Hz, 3H). $^{13}$C NMR (101 MHz, $CDCl_3$):

δ 165.66, 131.74, 129.53, 128.50, 127.28, 63.80, 29.77, 18.26, 12.73. IR (KBr): 2960, 2362, 2335, 1723, 1516, 1462, 1268, 1102, 1021, 801, 749 $cm^{-1}$. MS (EI, m/z): 178 $[M]^+$.

**isobutyl benzoate (3ad)**

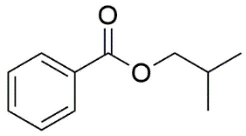

Colorless oil (71 mg, 80% yield); $^1$H NMR (400 MHz, $CDCl_3$): δ 7.98 (d, *J* = 6.8 Hz, 2H), 7.48 (t, *J* = 7.6 Hz, 1H), 7.38 (t, *J* = 7.6 Hz, 2H), 4.04 (d, *J* = 6.8 Hz, 2H), 1.98-2.05 (m, 1H), 0.96 (s, 3H), 0.95 (s, 3H). $^{13}$C NMR (101 MHz, $CDCl_3$): δ 166.78, 132.94, 130.69, 129.67, 128.47, 71.16, 28.07, 19.35. IR (KBr): 2957, 2922, 2362, 2335, 1724, 1515, 1459, 1374, 1267, 1102, 1022, 800, 753, 709 $cm^{-1}$. MS (EI, m/z): 178 $[M]^+$.

**methyl 3-phenylpropanoate (3za)[3]**

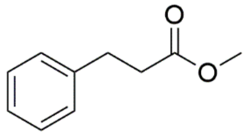

Colorless oil (41 mg, 50% yield); $^1$H NMR (400 MHz, $CDCl_3$): δ 7.26-7.30 (m, 2H), 7.17-7.21 (m, 3H), 3.66 (s, 3H), 2.95 (d, *J* = 8.0 Hz, 2H), 2.62 (t, *J* = 8.0 Hz, 2H). $^{13}$C NMR (101 MHz, $CDCl_3$): δ 173.38, 140.61, 128.59, 128.35, 126.35, 51.67, 35.79, 31.05. IR (KBr): 3062, 3028, 2951, 2362, 2335, 1747, 1496, 1443, 1363, 1199, 1163, 912, 835, 745, 699 $cm^{-1}$. MS (EI, m/z): 164 $[M]^+$.

## 13.References


(1) L. L. Chng, J. Yang, J. Y. Ying, Chemsuschem, 2015, 8, 1916-1925.

(2) J. H. Xia, A. L. Shao, S. Tang, X. L. Gao, M. Gao, A. W. Lei, Org. Biomol. Chem., 2015, 13, 6154-6157.

(3) X. F. Wu, C. Darcel, Eur. J. Org. Chem., 2009, 1144-1147.

(4) C. Liu, J. Wang, L. K. Meng, Y. Deng, Y. Li, A. W. Lei, Angew. Chem., Int. Ed., 2011, 50, 5144-5148.

(5) D. Craig, G. D. Henry, Tetrahedron Lett., 2005, 46, 2559-2562.

## 14. $^{1}$H-NMR and $^{13}$C-NMR spectra of the obtained compounds

### $^{1}$H-NMR spectrum of 3aa

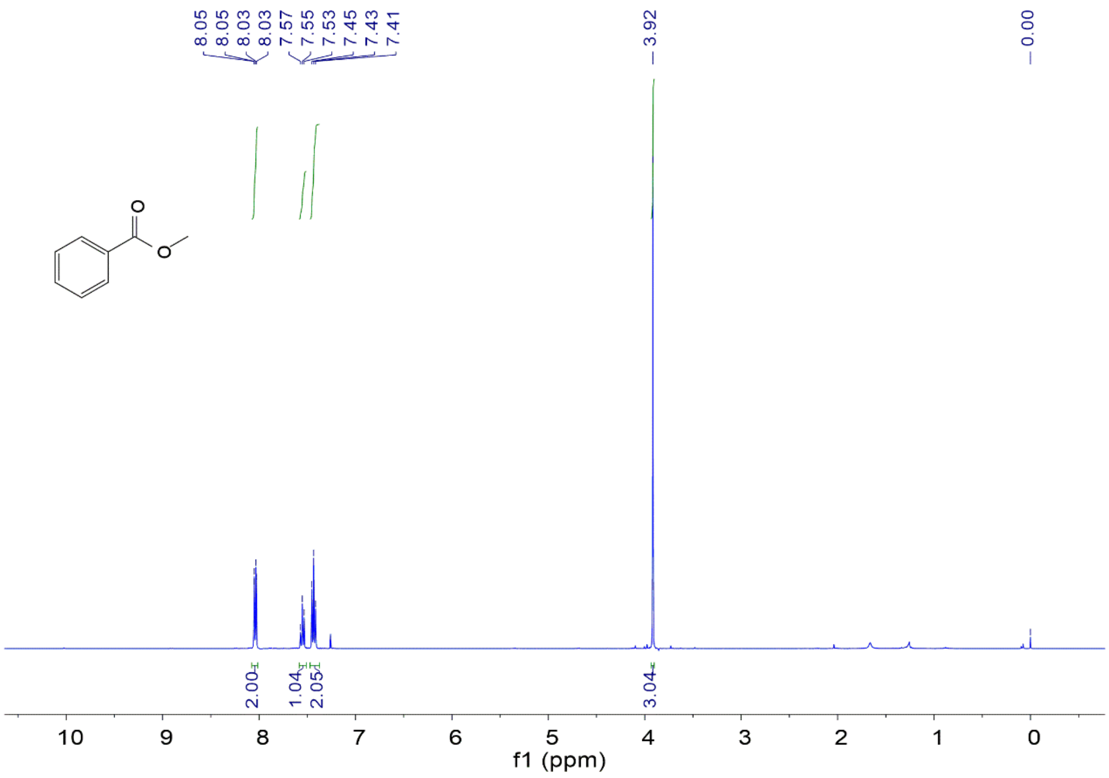


### $^{13}$C-NMR spectrum of 3aa

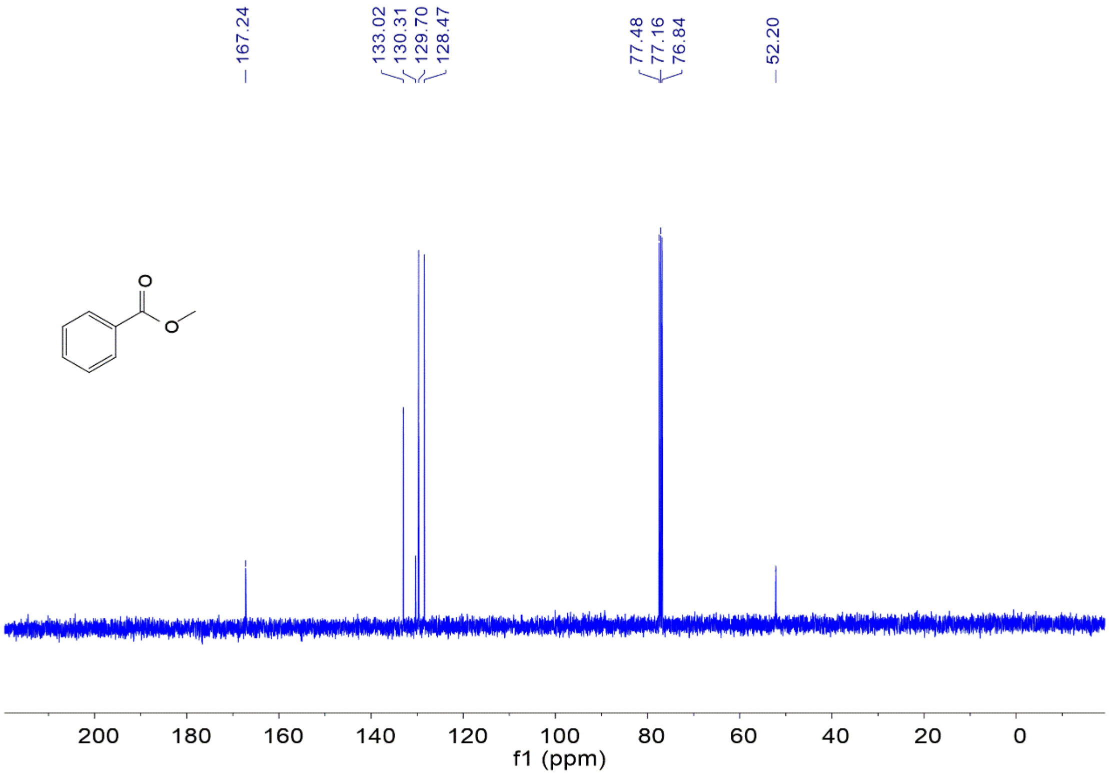

## $^{1}$H-NMR spectrum of 3ba

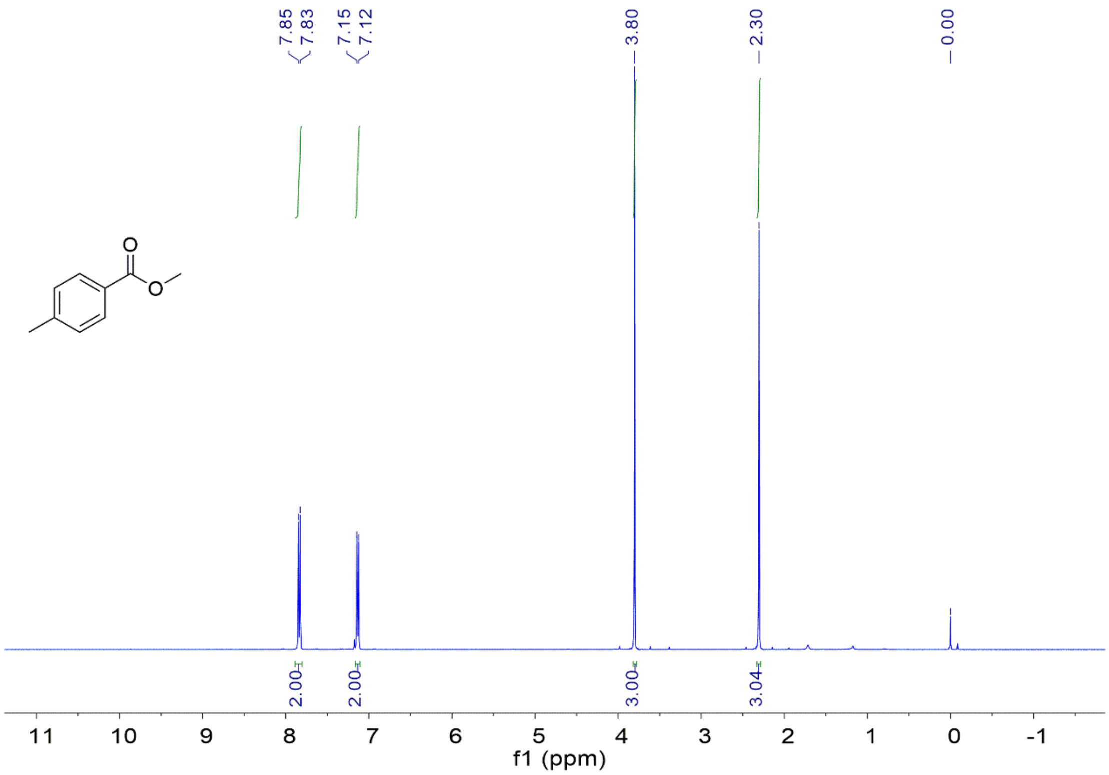


## $^{13}$C-NMR spectrum of 3ba

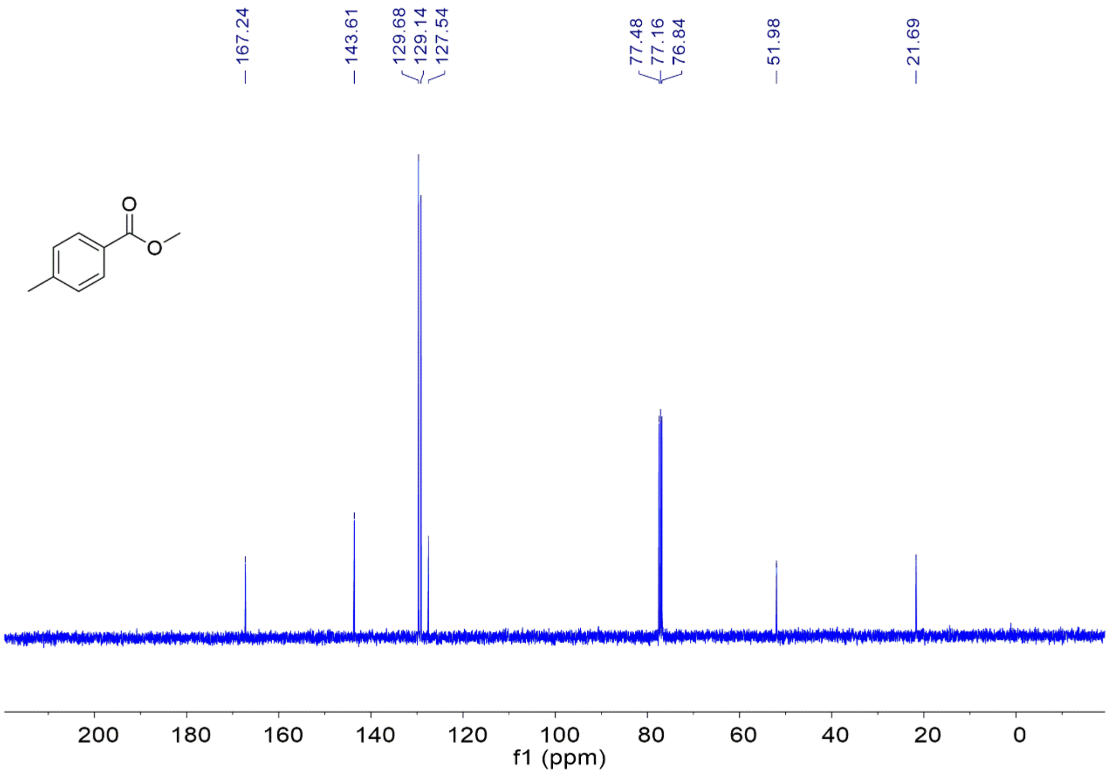

## $^{1}$H-NMR spectrum of 3ca

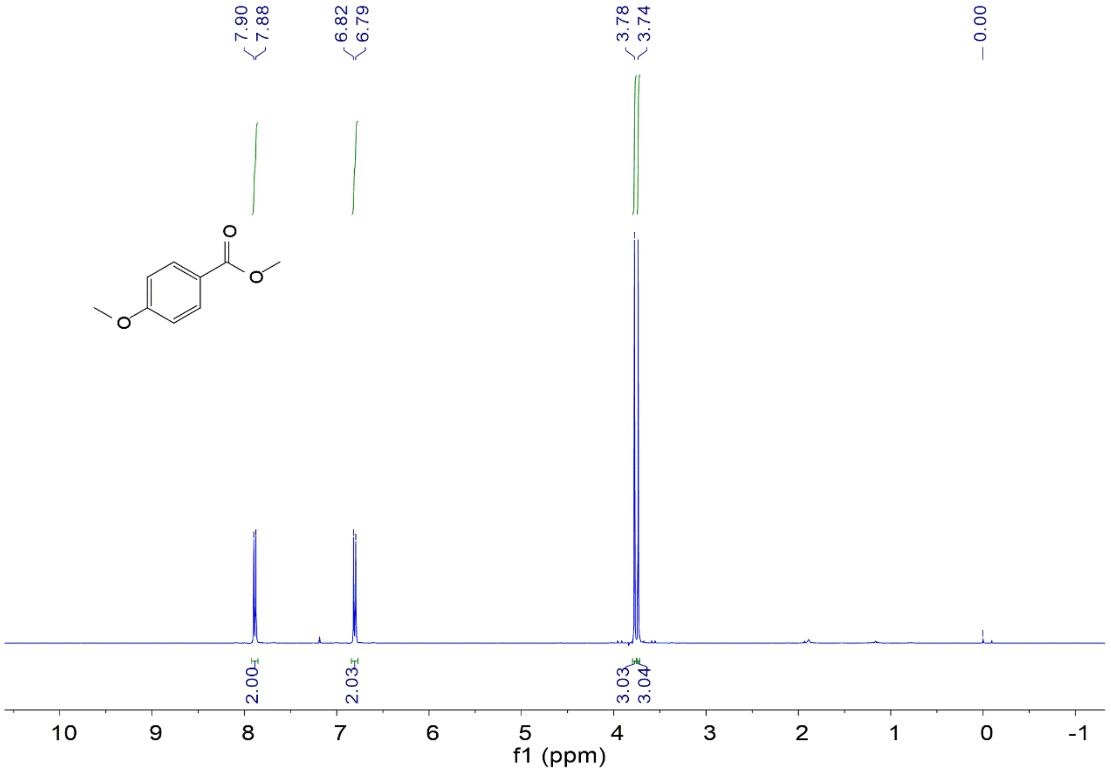


## $^{13}$C-NMR spectrum of 3ca

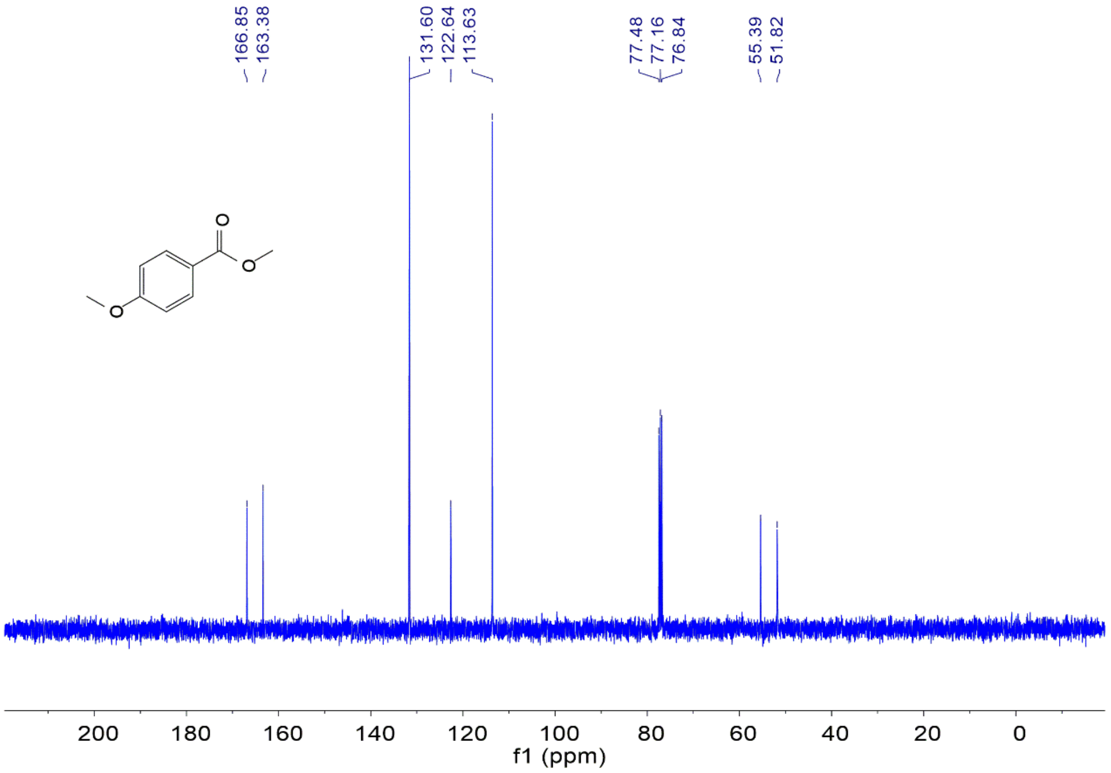

## $^{1}$H-NMR spectrum of 3da

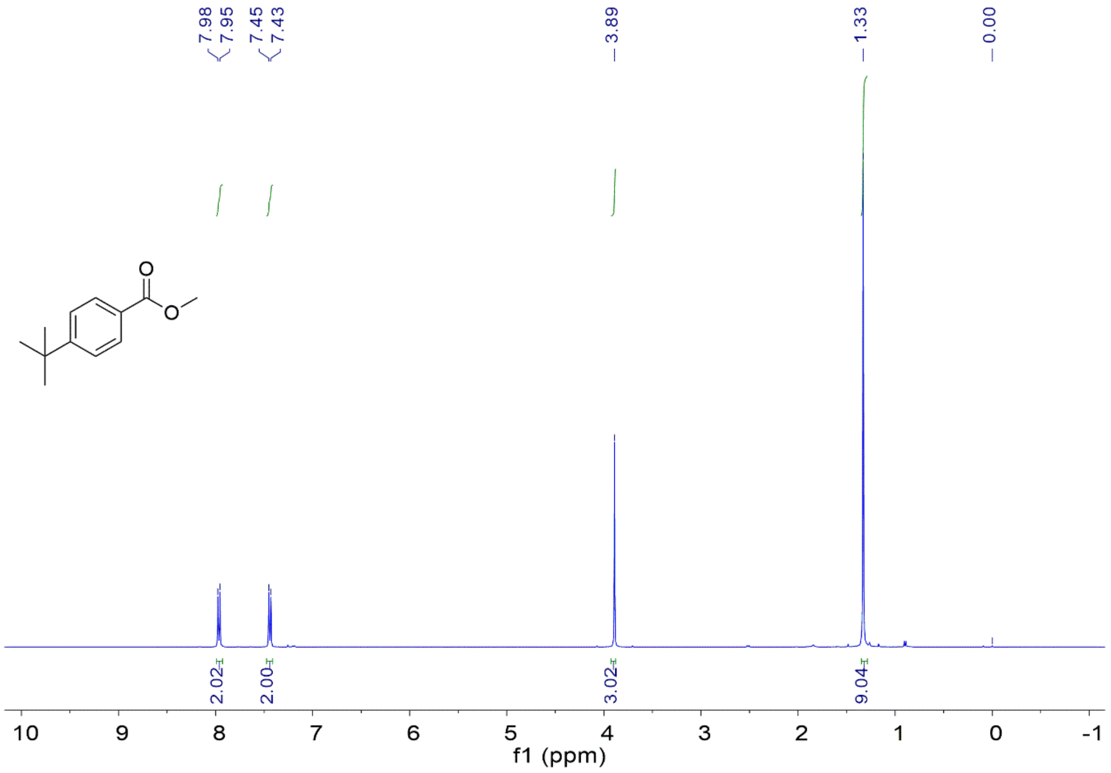


## $^{13}$C-NMR spectrum of 3da

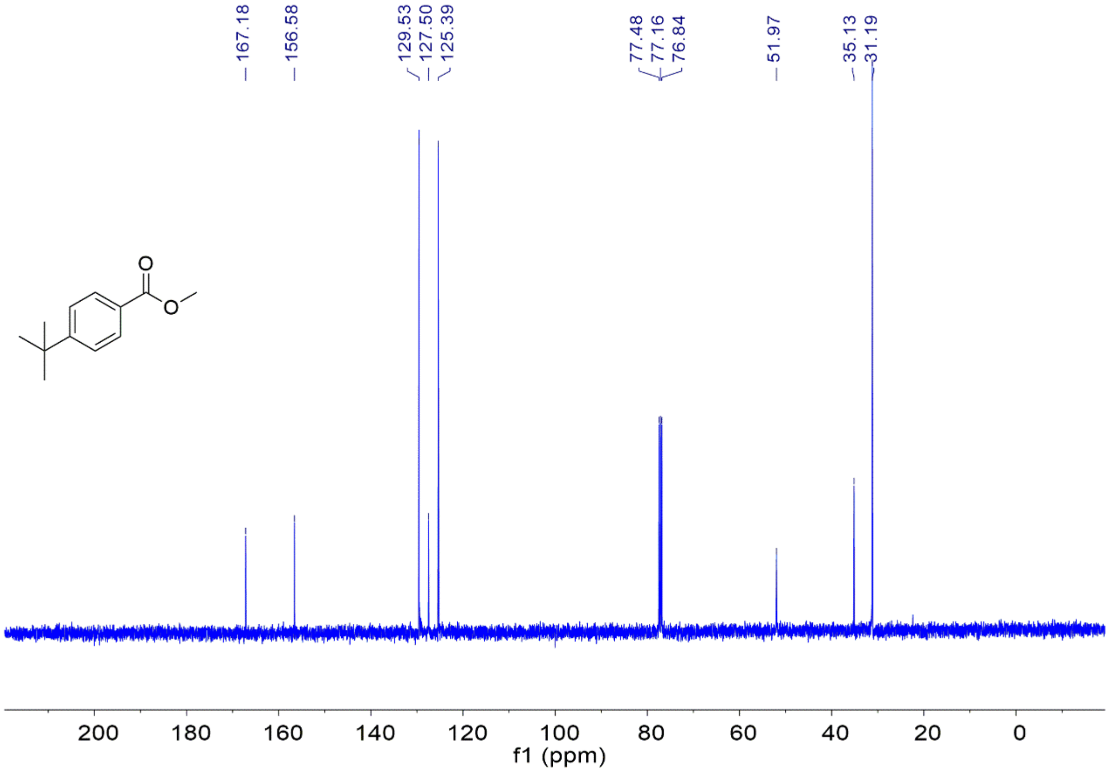

## $^{1}$H-NMR spectrum of 3ea

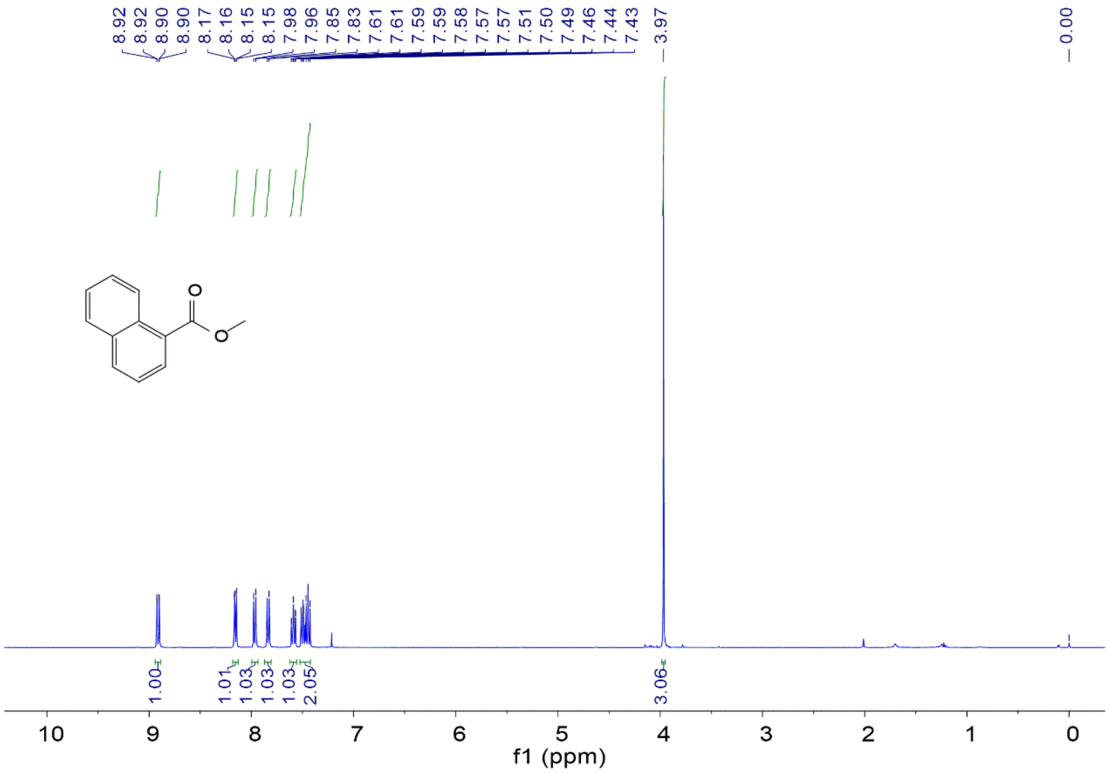


## $^{13}$C-NMR spectrum of 3ea

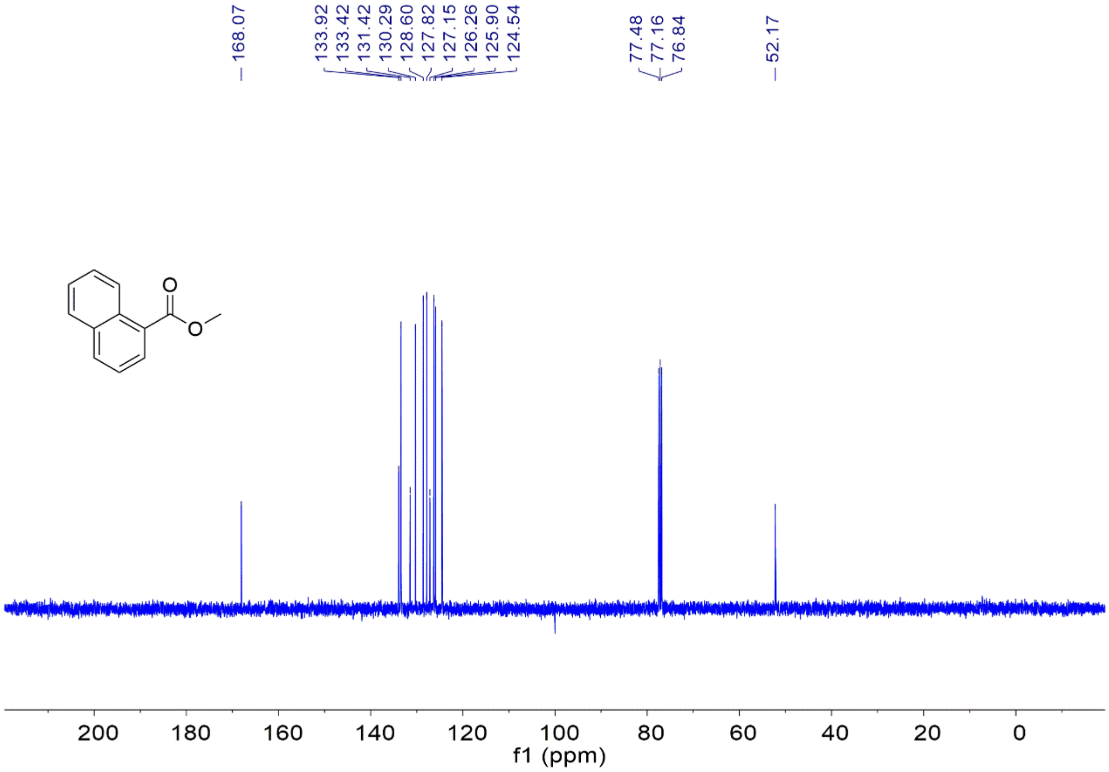

## $^{1}$H-NMR spectrum of 3fa

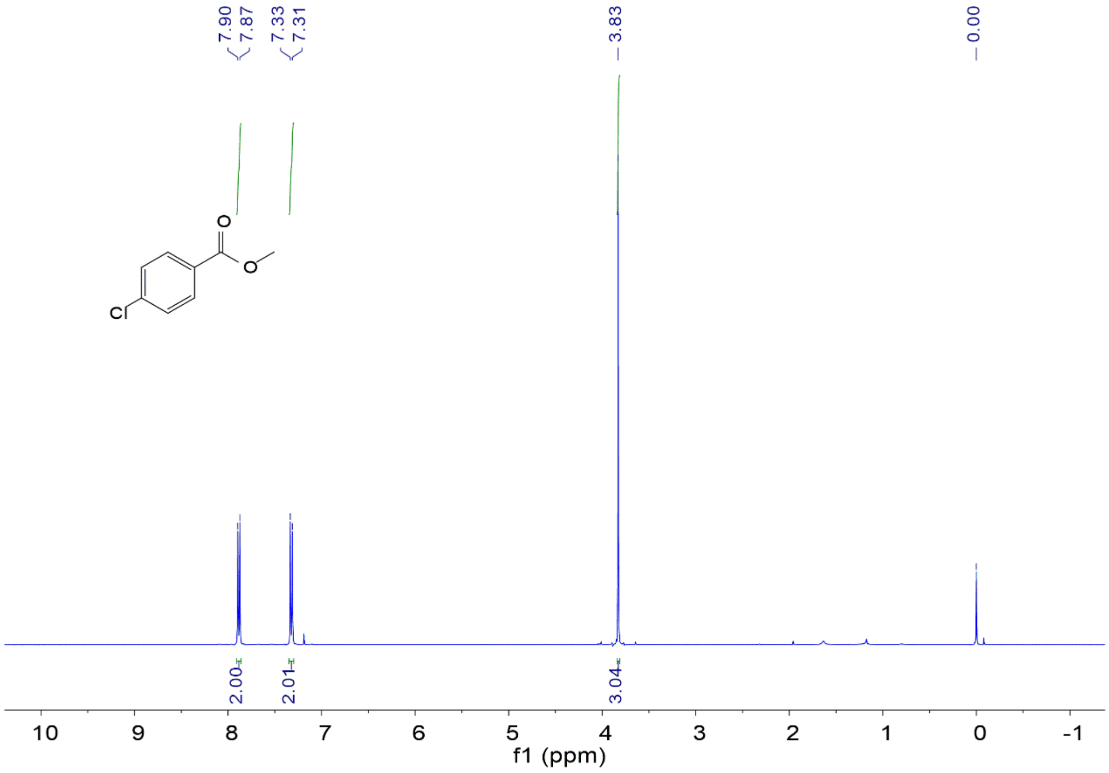


## $^{13}$C-NMR spectrum of 3fa

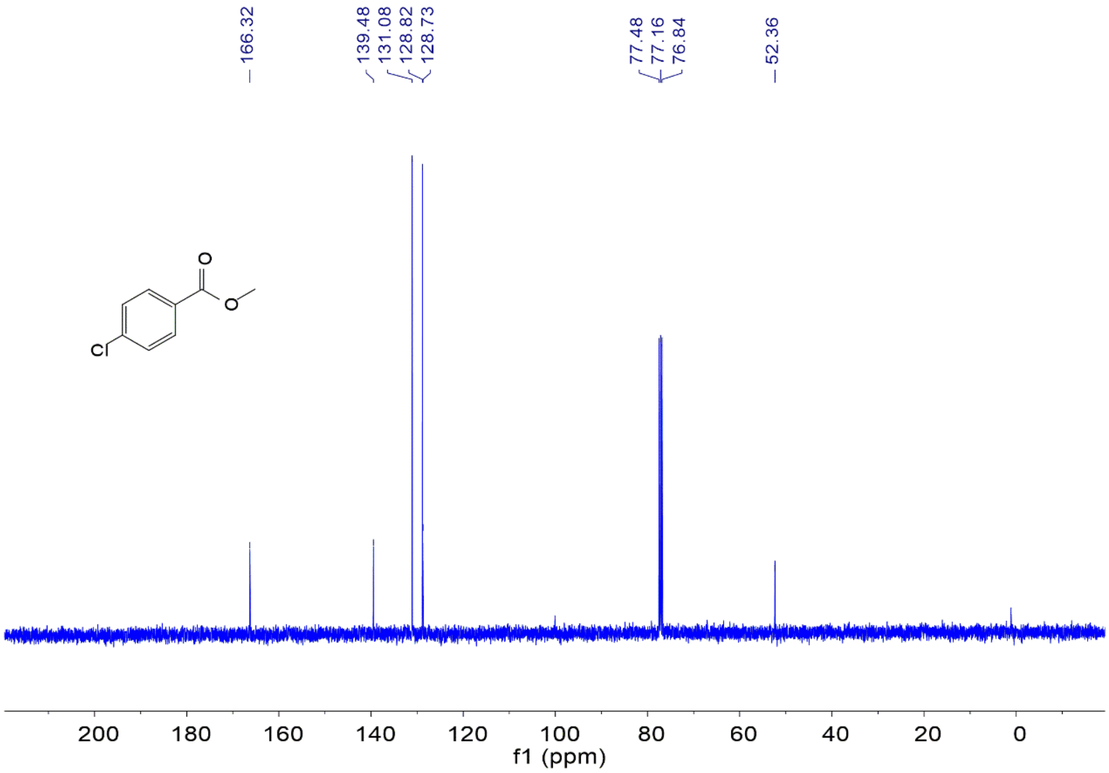

## $^{1}H$-NMR spectrum of 3ga

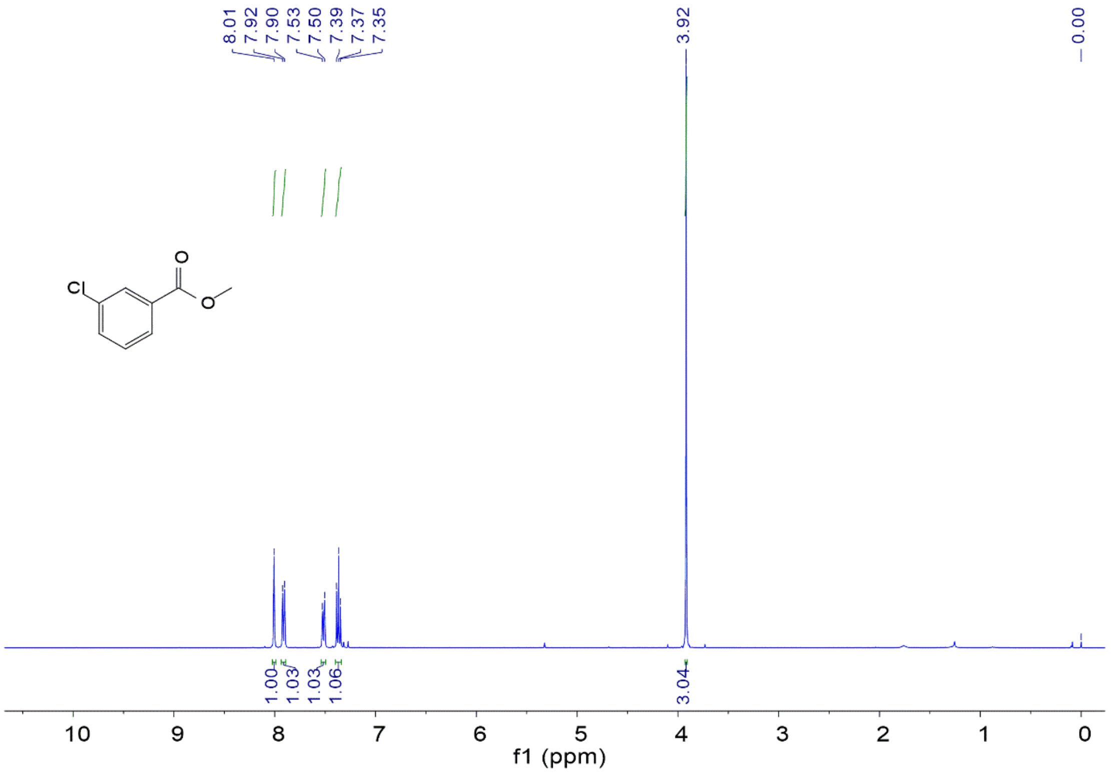


## $^{13}C$-NMR spectrum of 3ga

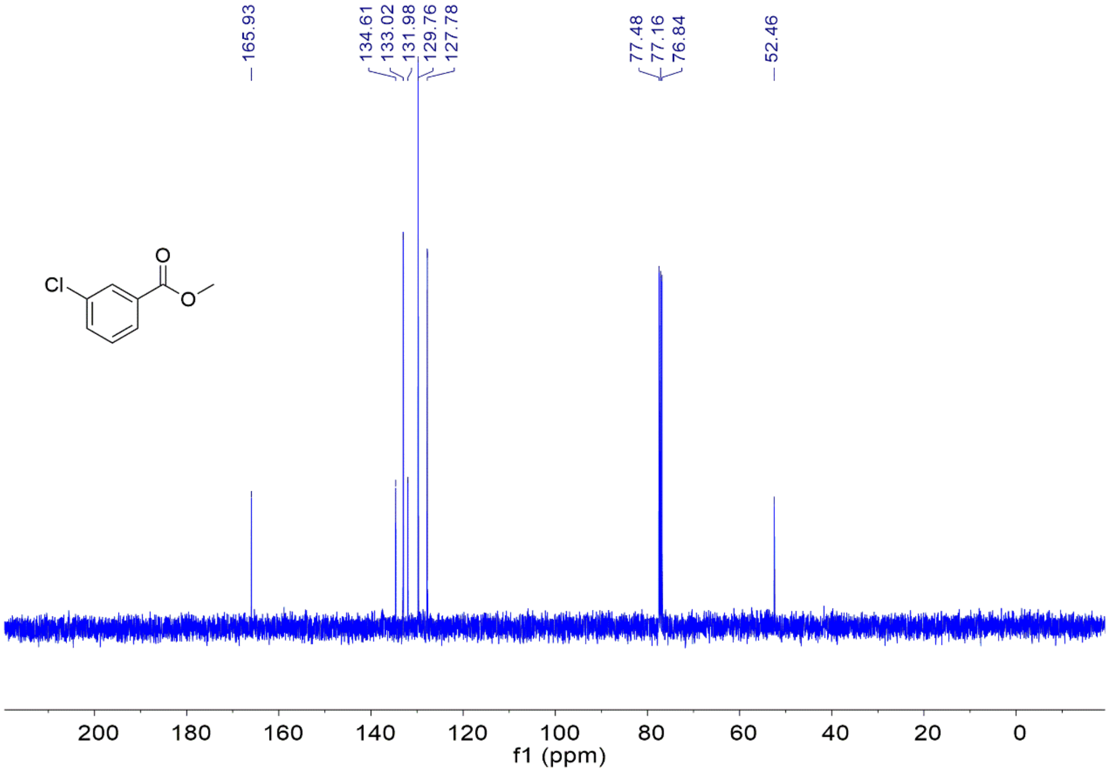

## $^{1}$H-NMR spectrum of 3ha

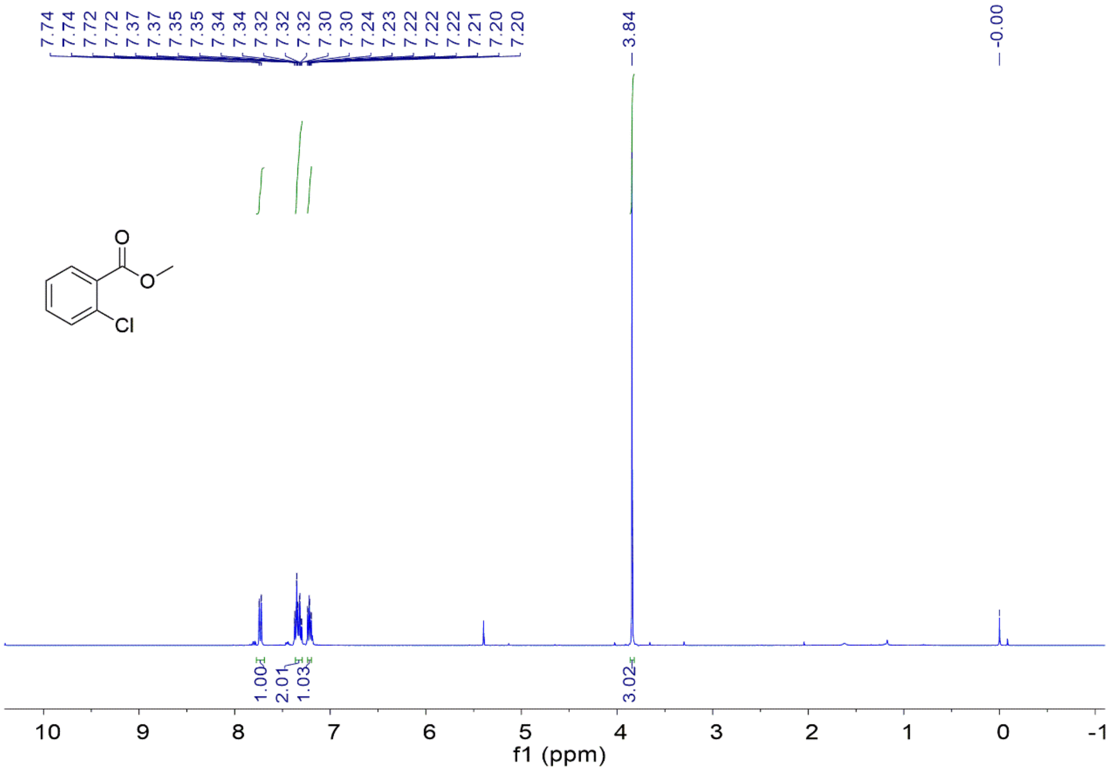


## $^{13}$C-NMR spectrum of 3ha

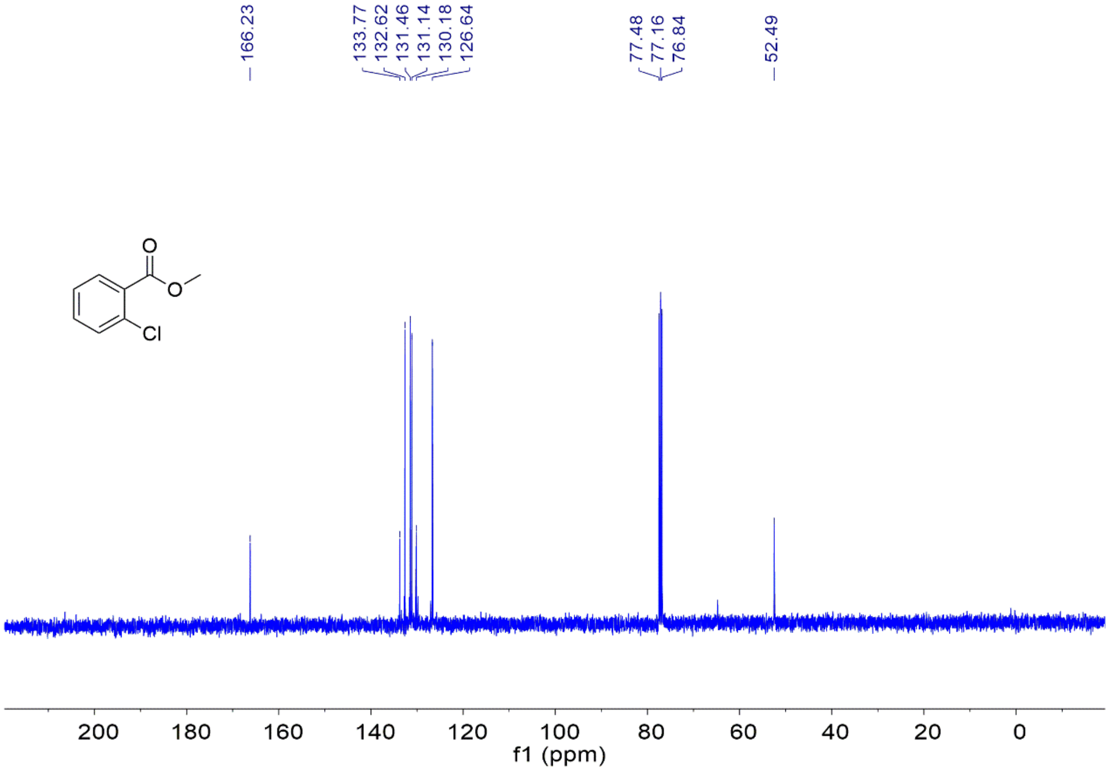

**$^{1}$H-NMR spectrum of 3ia**

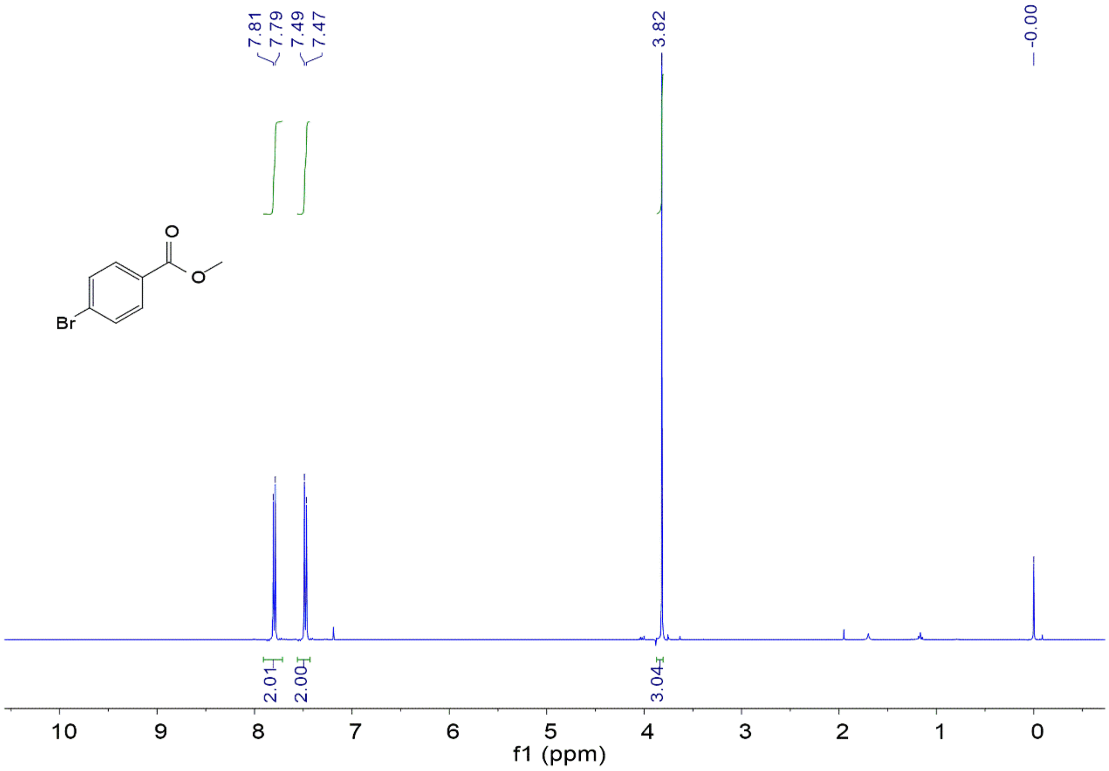


**$^{13}$C-NMR spectrum of 3ia**

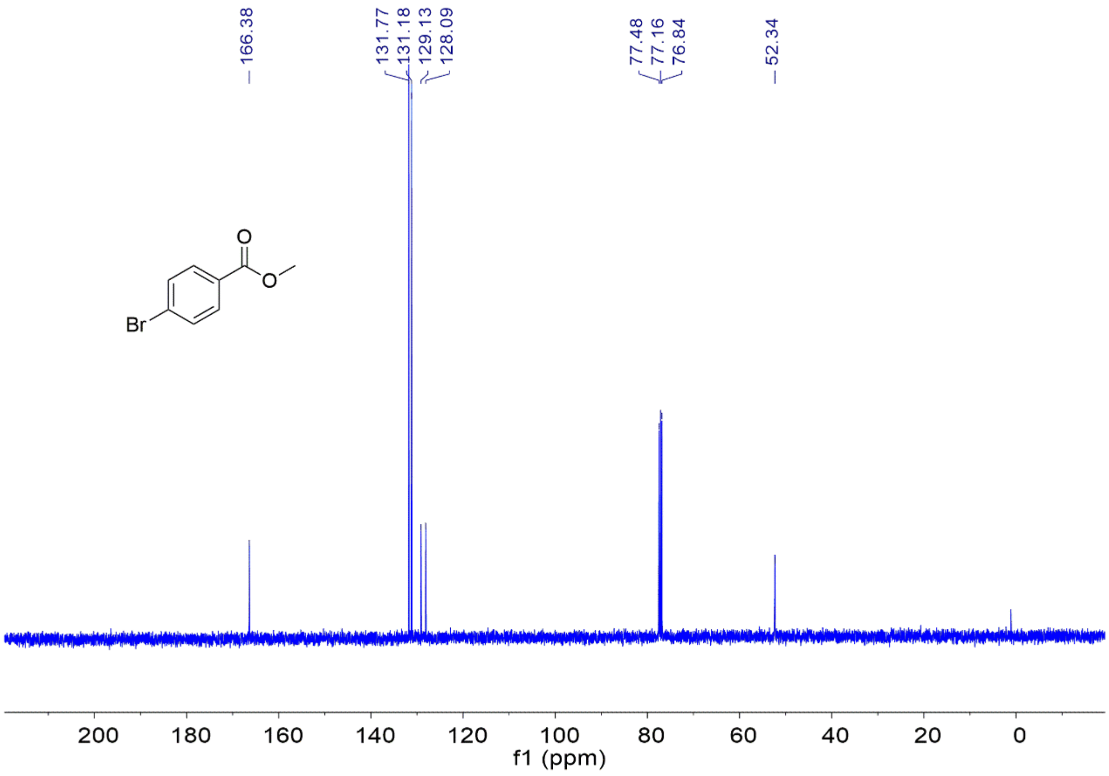

$^{1}$H-NMR spectrum of 3ja

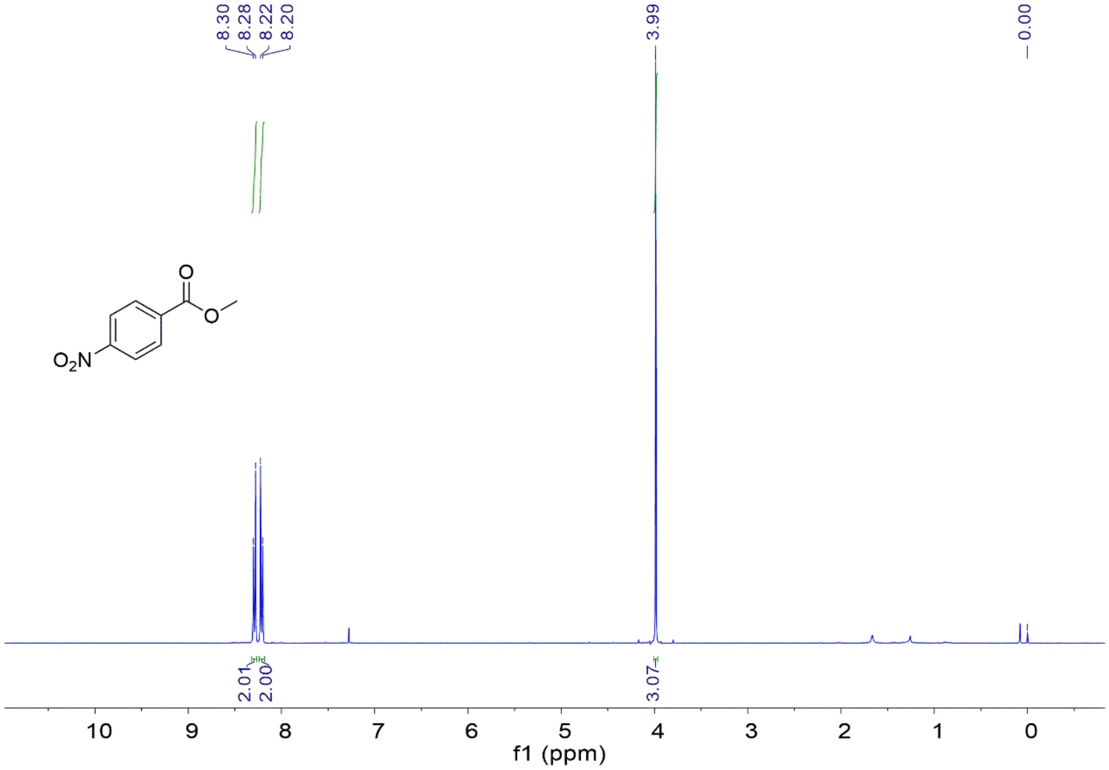


$^{13}$C-NMR spectrum of 3ja

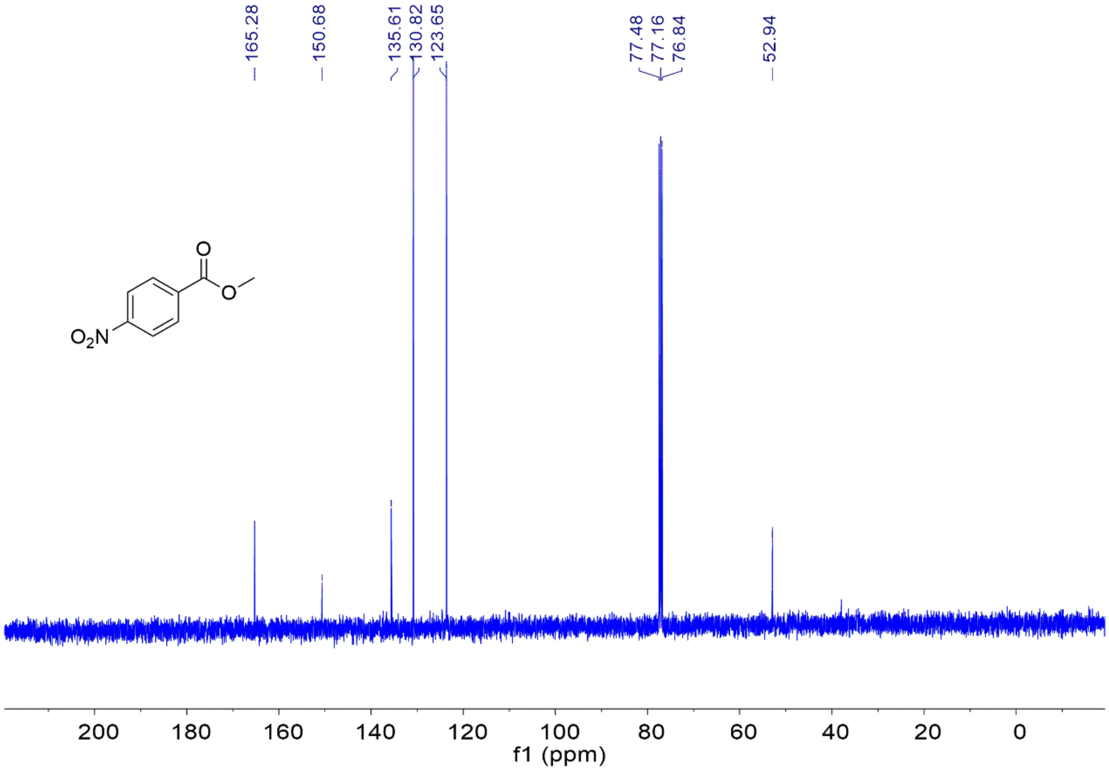

## $^{1}$H-NMR spectrum of 3ka

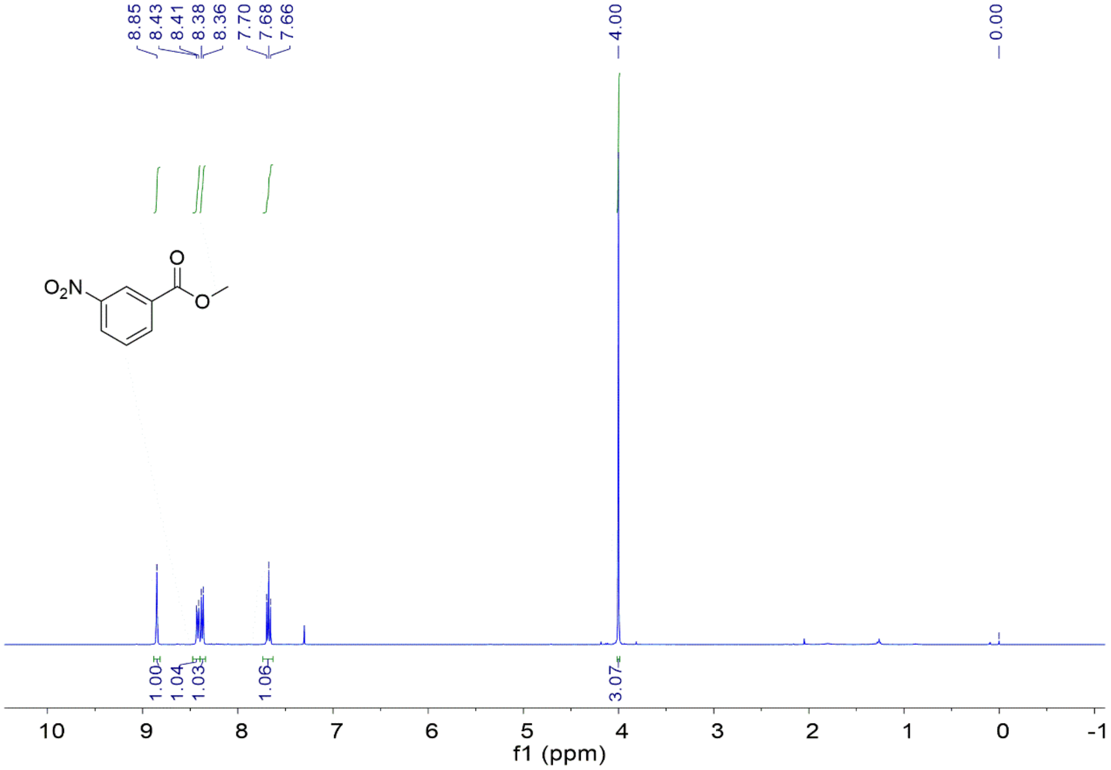


## $^{13}$C-NMR spectrum of 3ka

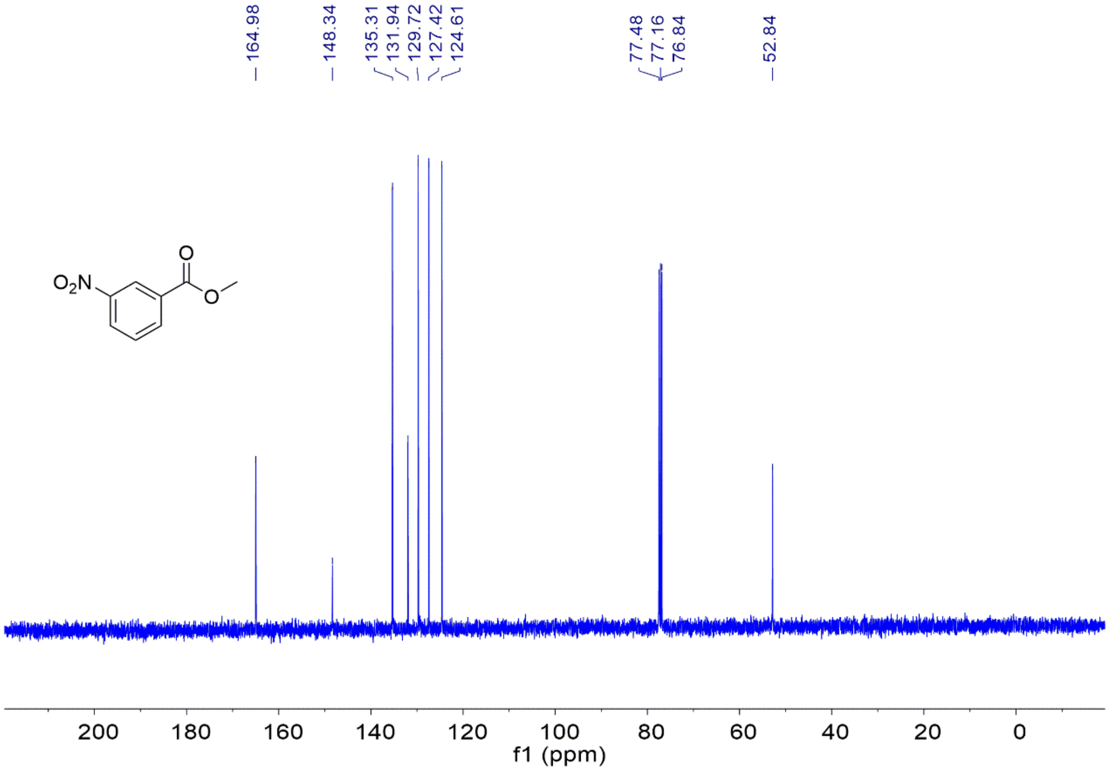

$^{1}$H-NMR spectrum of 3la

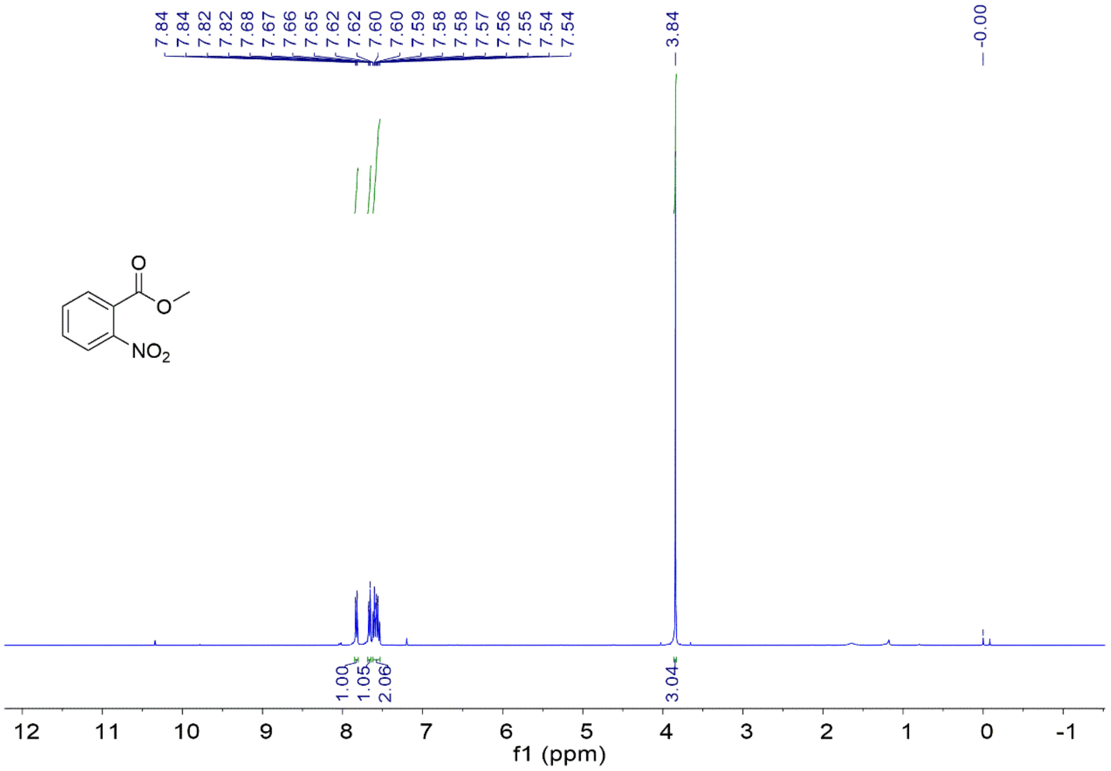


$^{13}$C-NMR spectrum of 3la

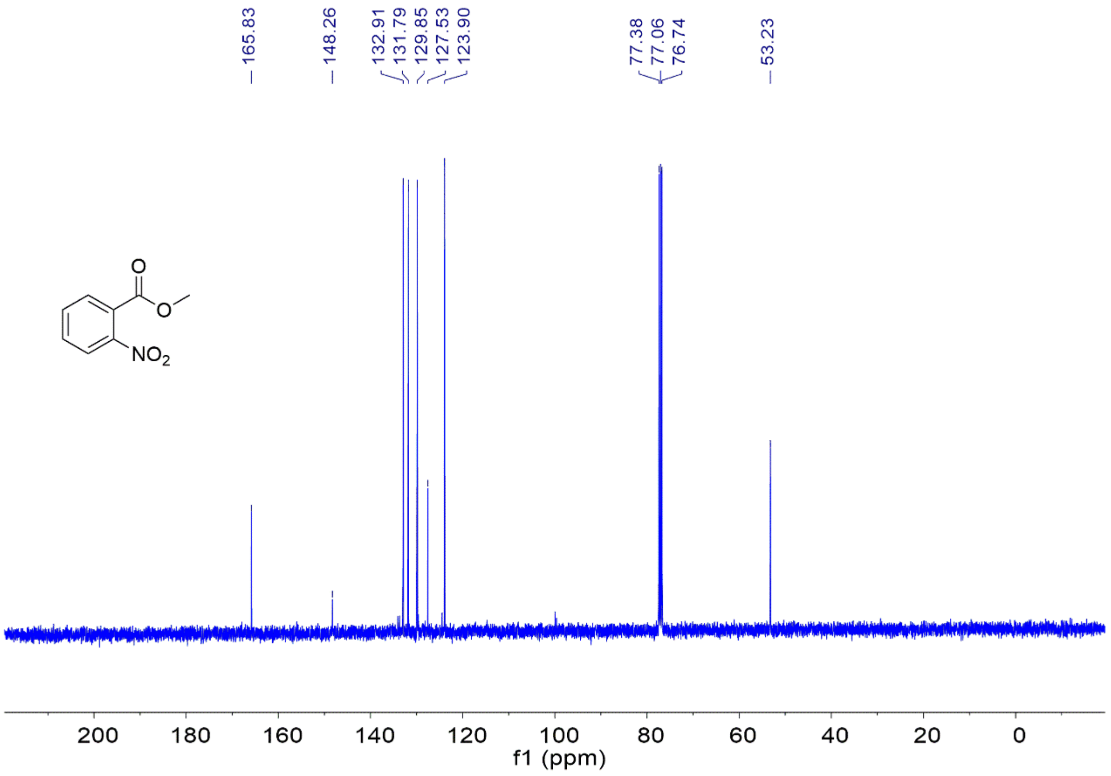

$^{1}$H-NMR spectrum of 3ma

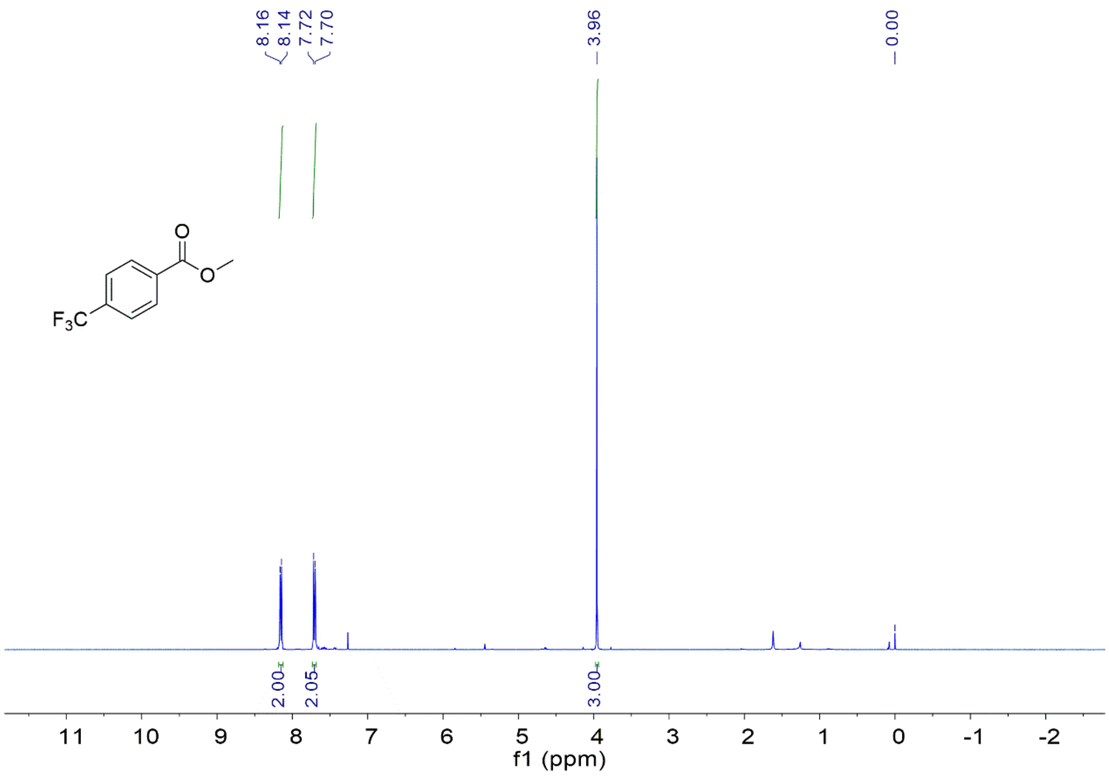


$^{13}$C-NMR spectrum of 3ma

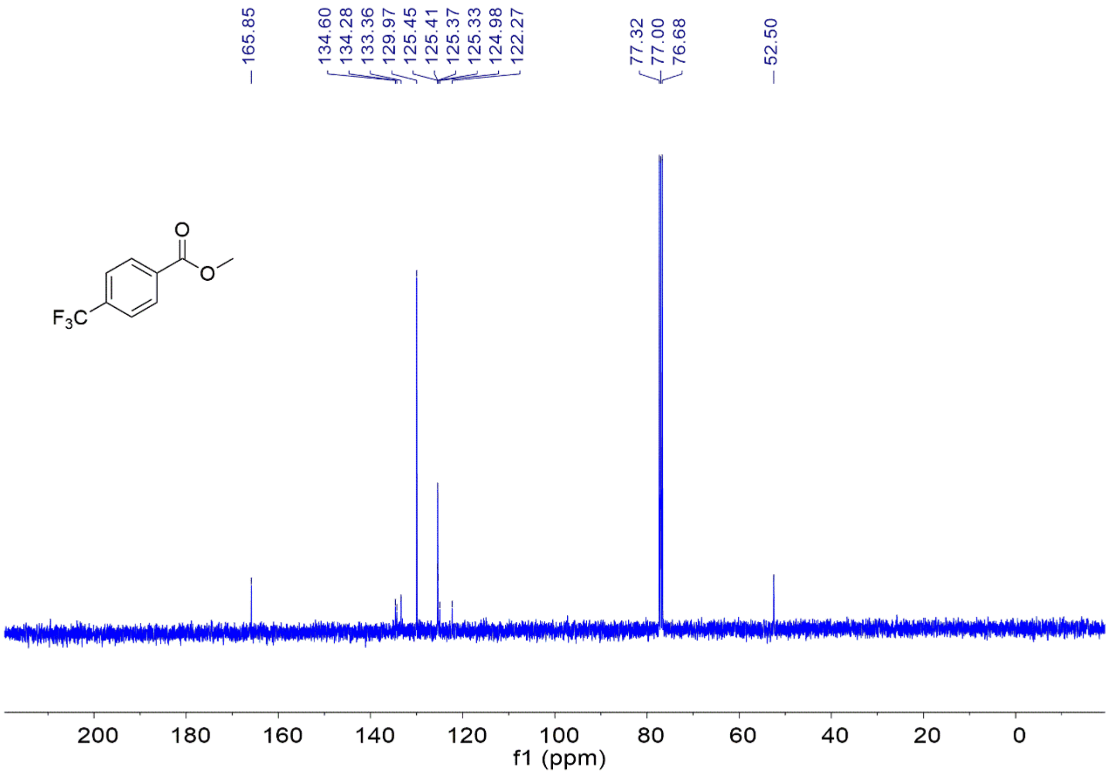

**$^{1}$H-NMR spectrum of 3na**

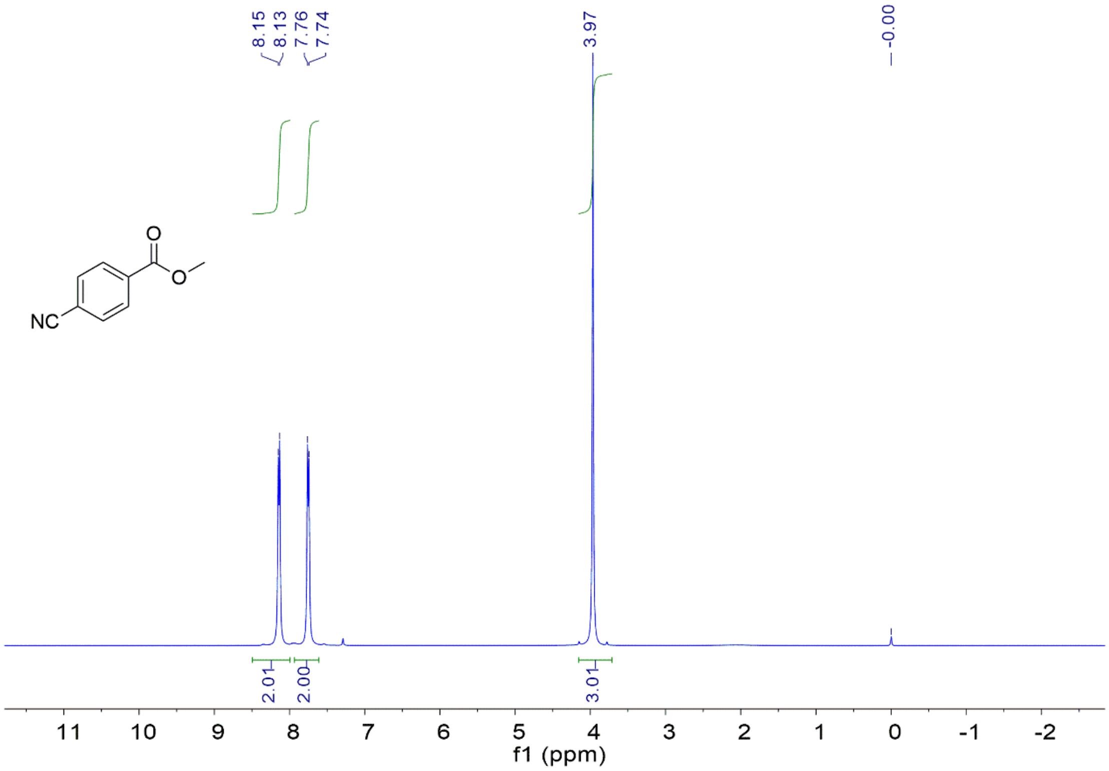


**$^{13}$C-NMR spectrum of 3na**

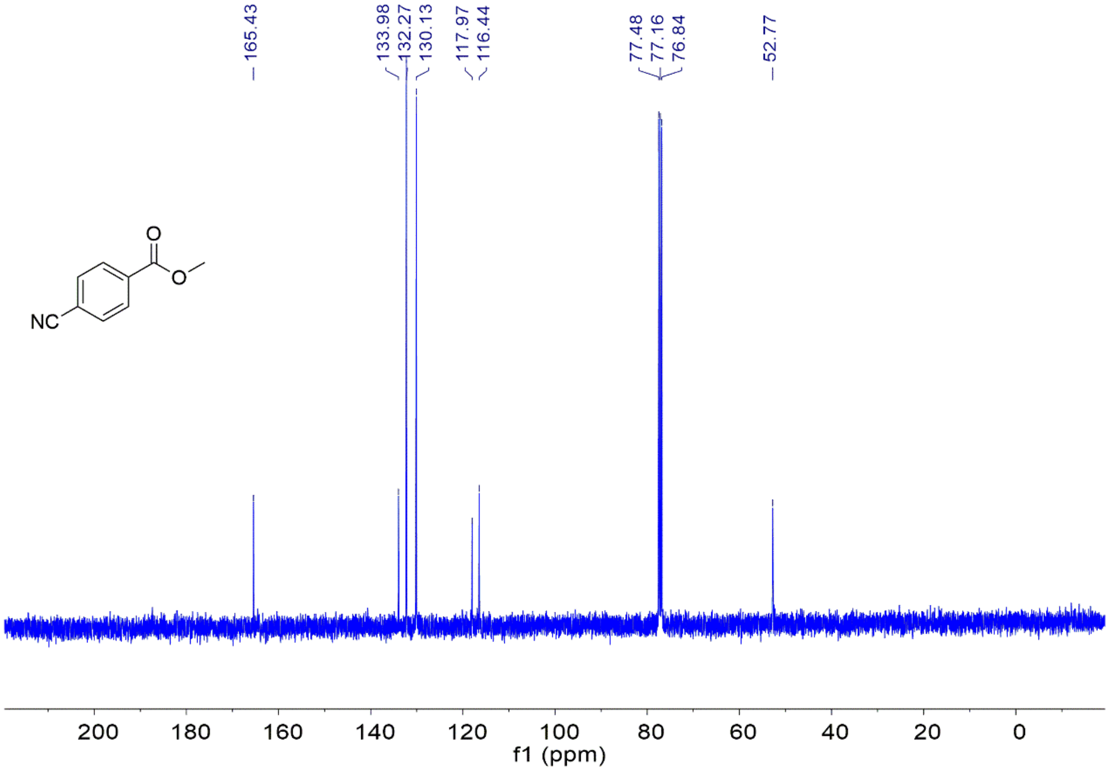

**$^{1}$H-NMR spectrum of 3oa**

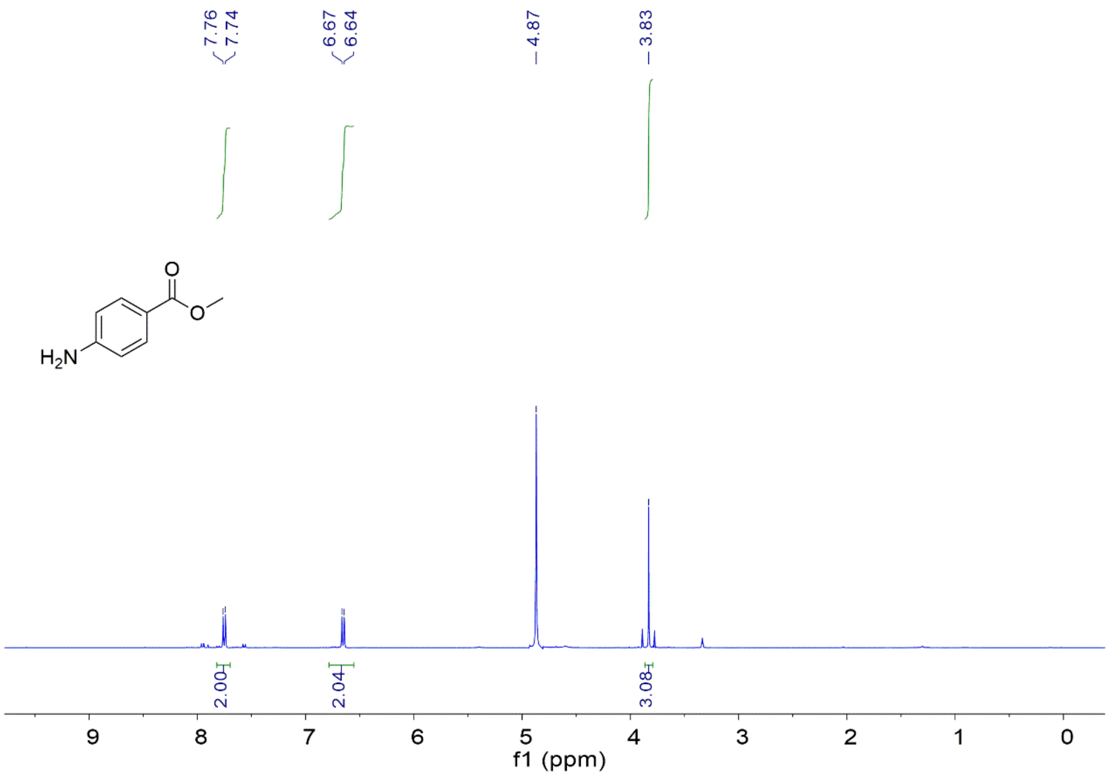


**$^{13}$C-NMR spectrum of 3oa**

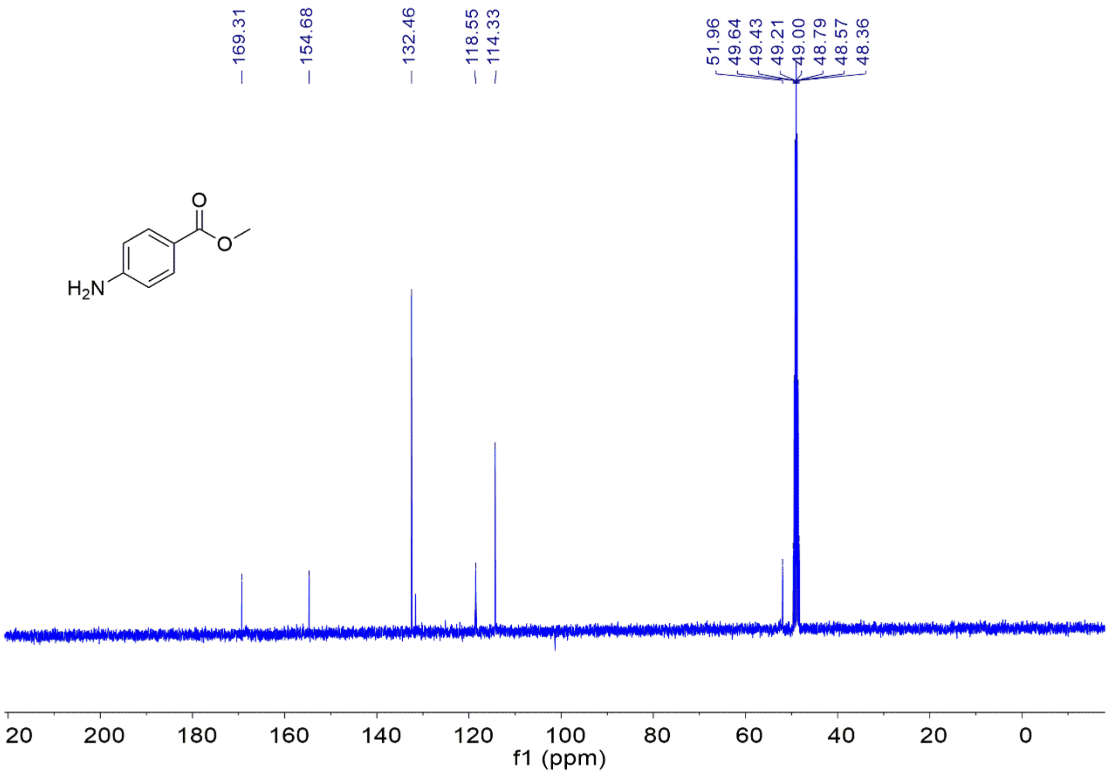

$^{1}$H-NMR spectrum of 3pa

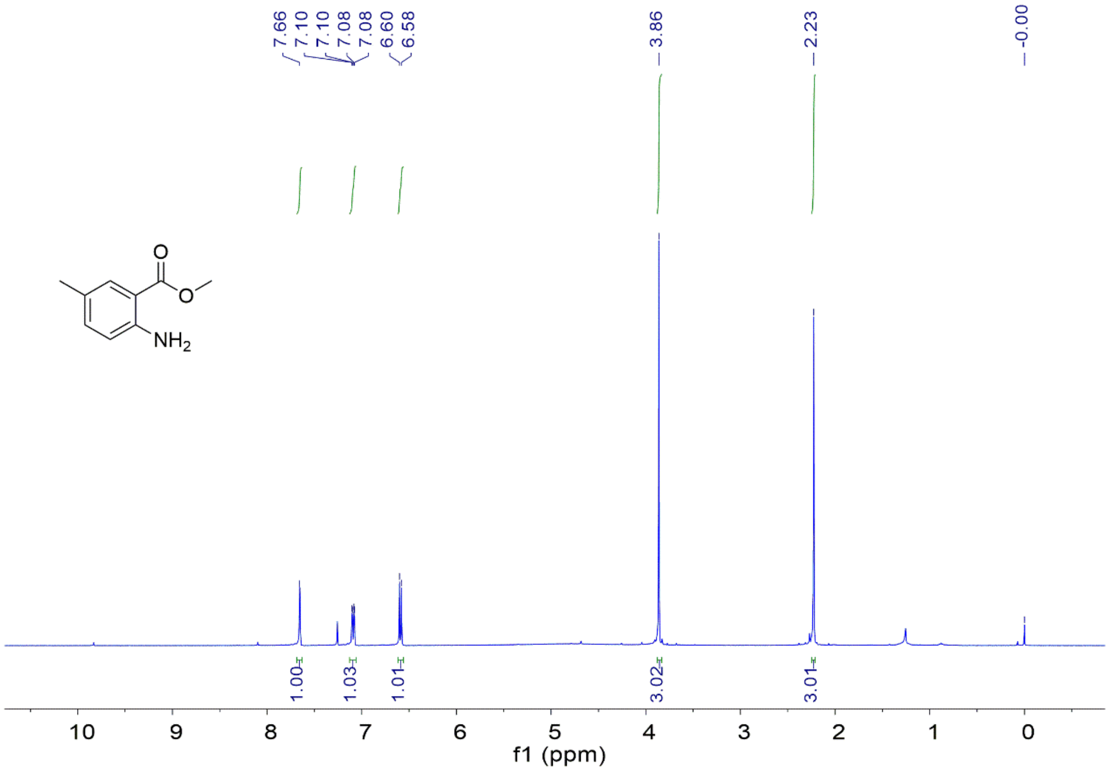


$^{13}$C-NMR spectrum of 3pa

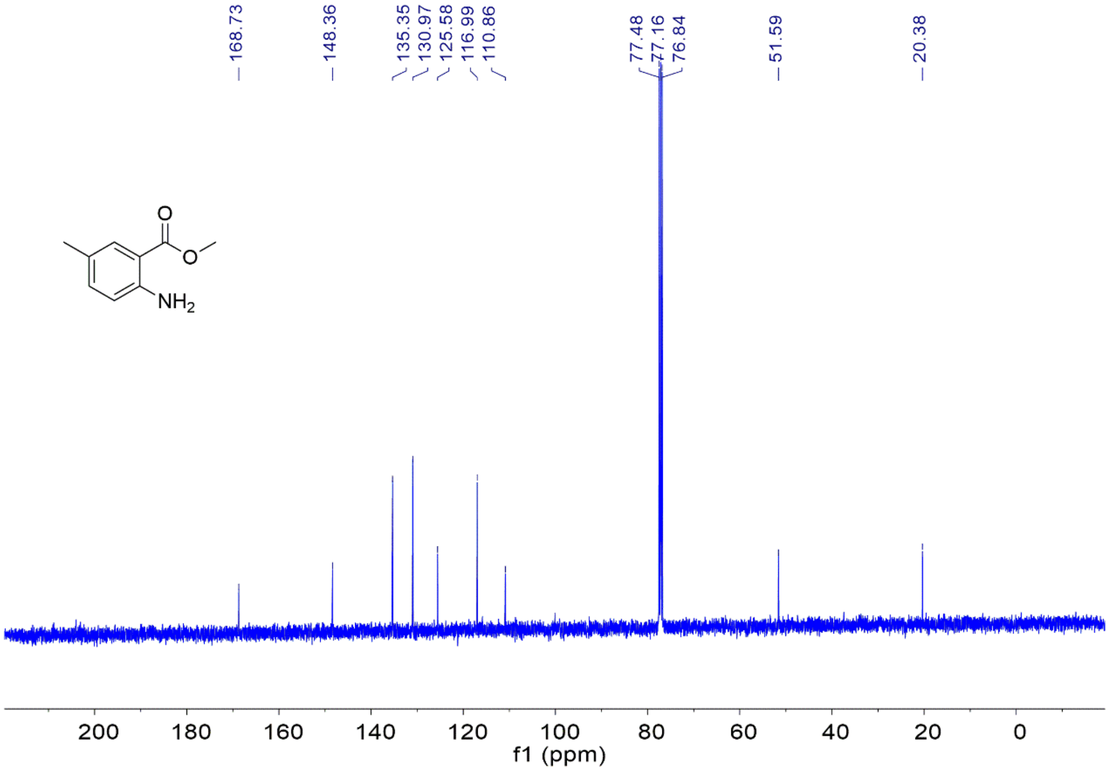

$^1$H-NMR spectrum of 3qa

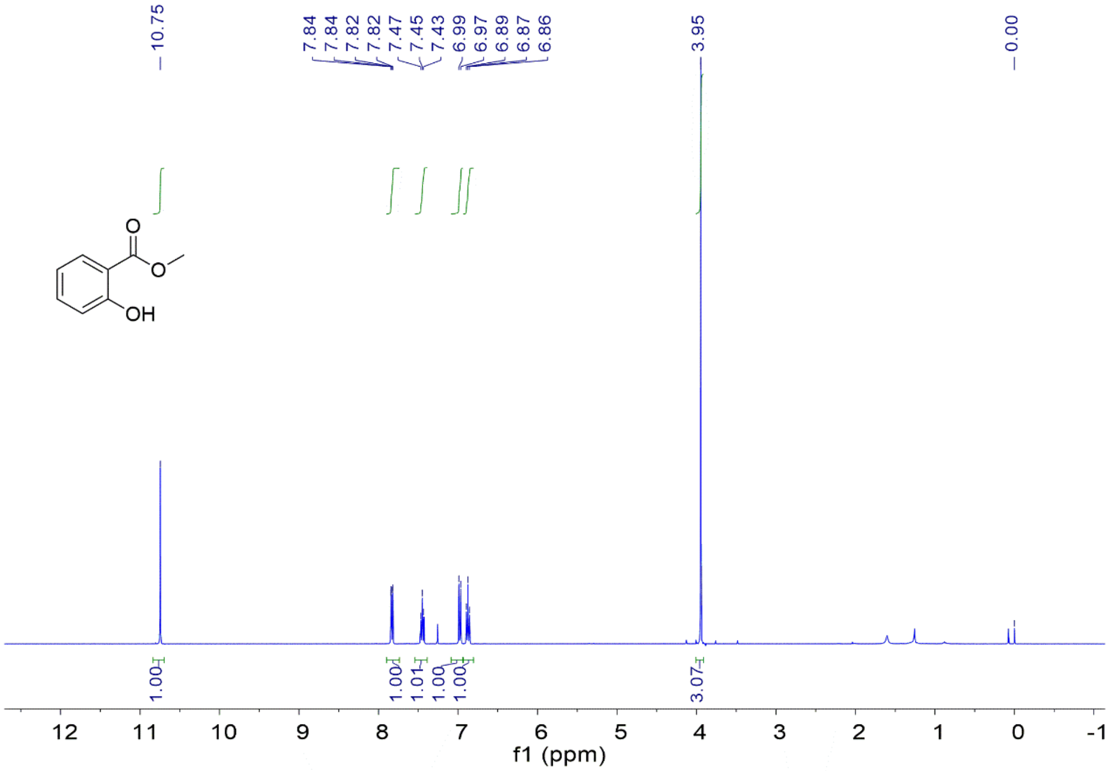


$^{13}$C- NMR spectrum of 3qa

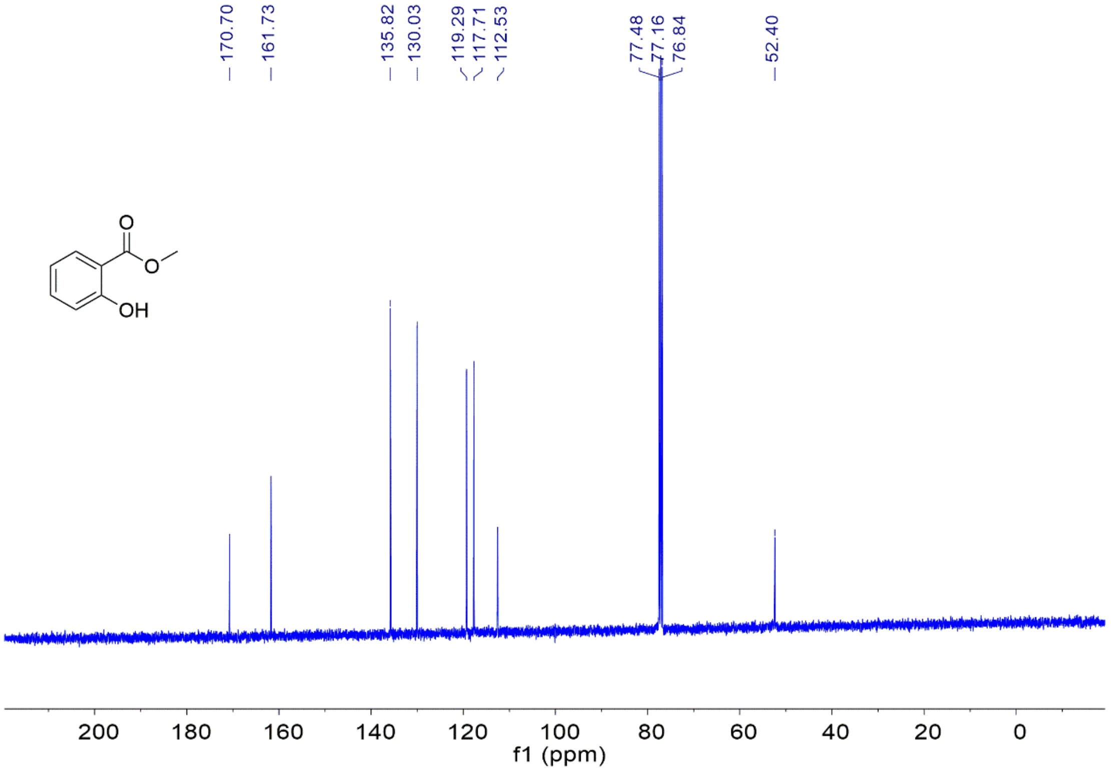

## $^{1}$H-NMR spectrum of 3ra

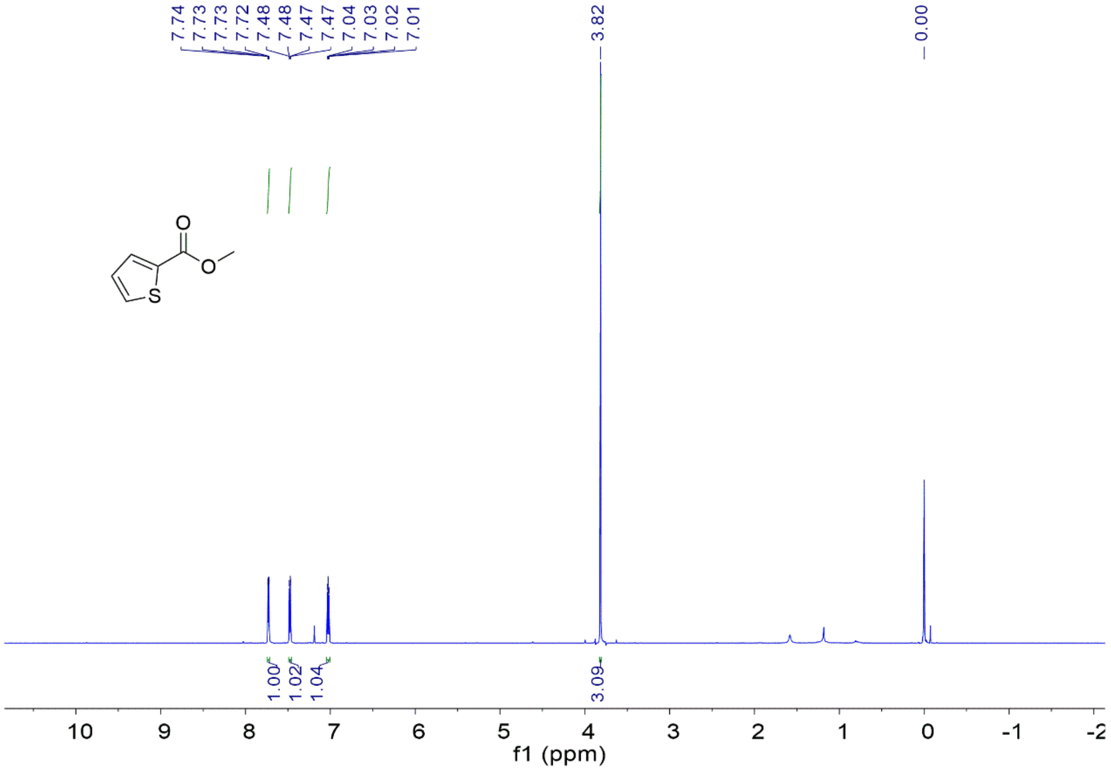


## $^{13}$C-NMR spectrum of 3ra

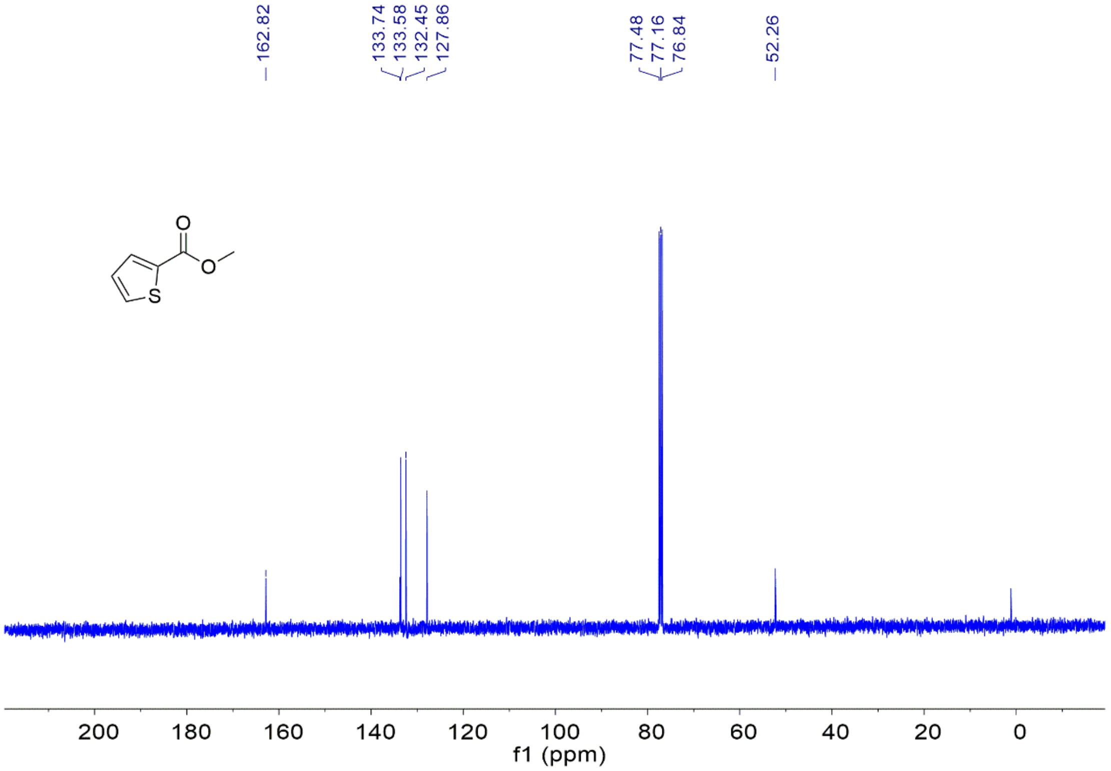

**$^{1}$H-NMR spectrum of 3sa**

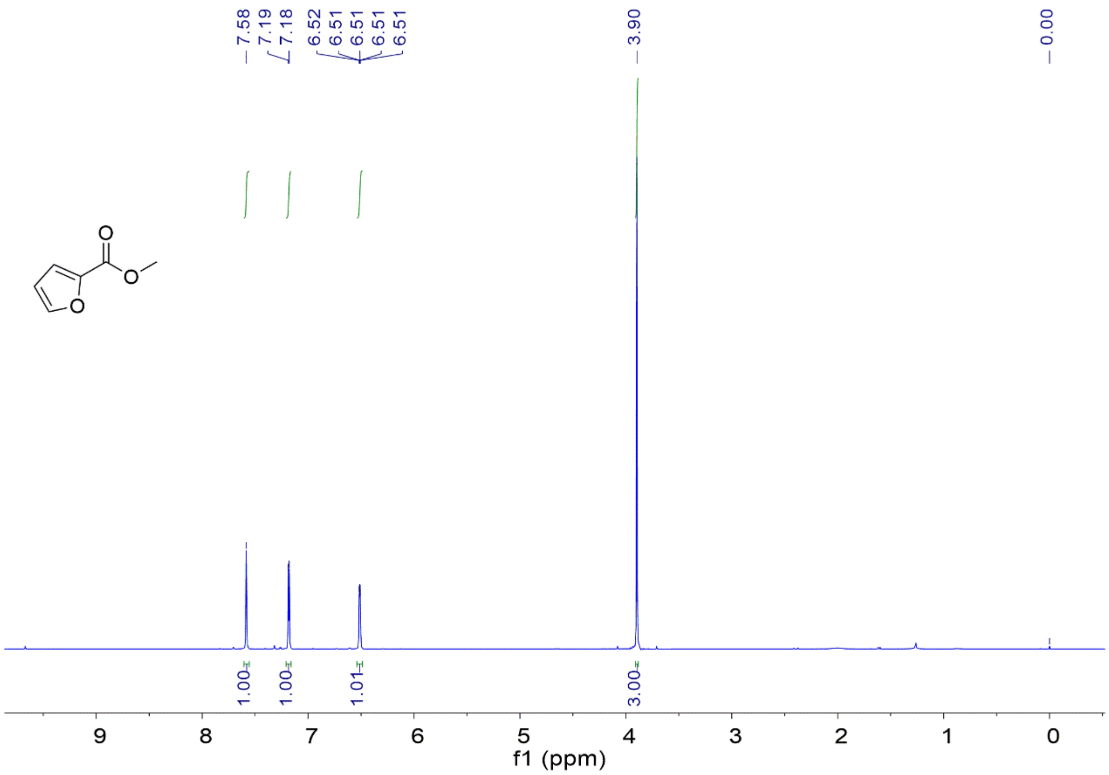


**$^{13}$C-NMR spectrum of 3sa**

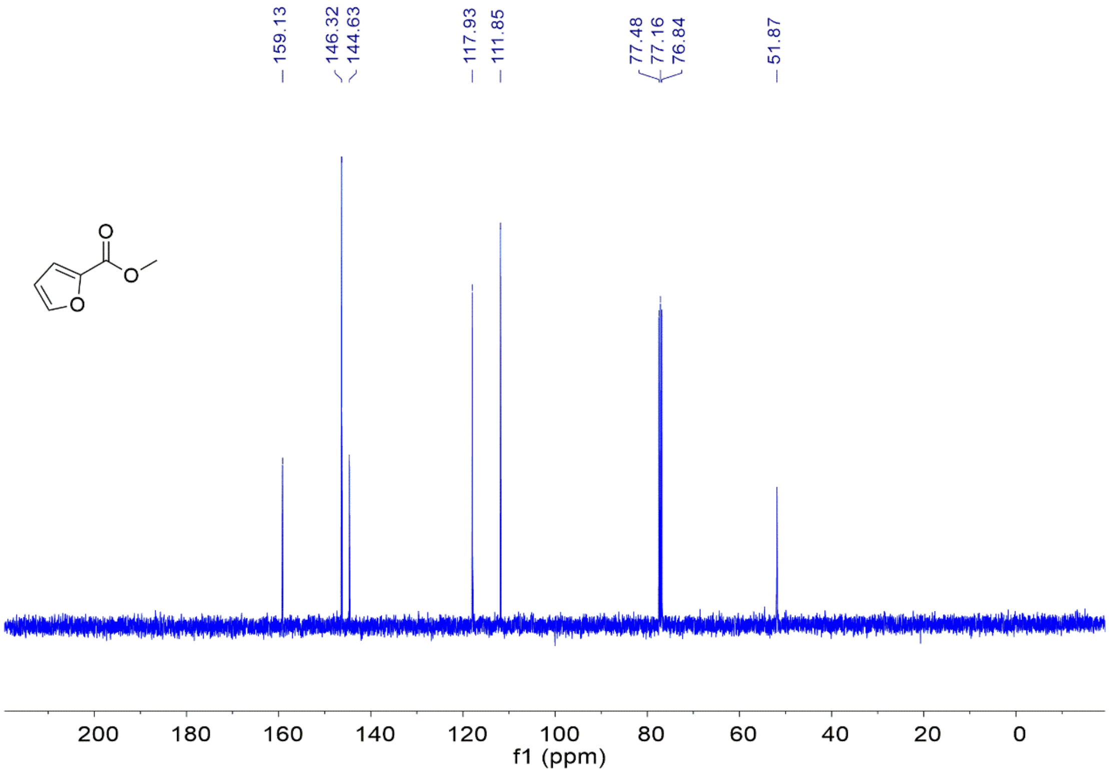

## $^{1}$H-NMR spectrum of 3ta

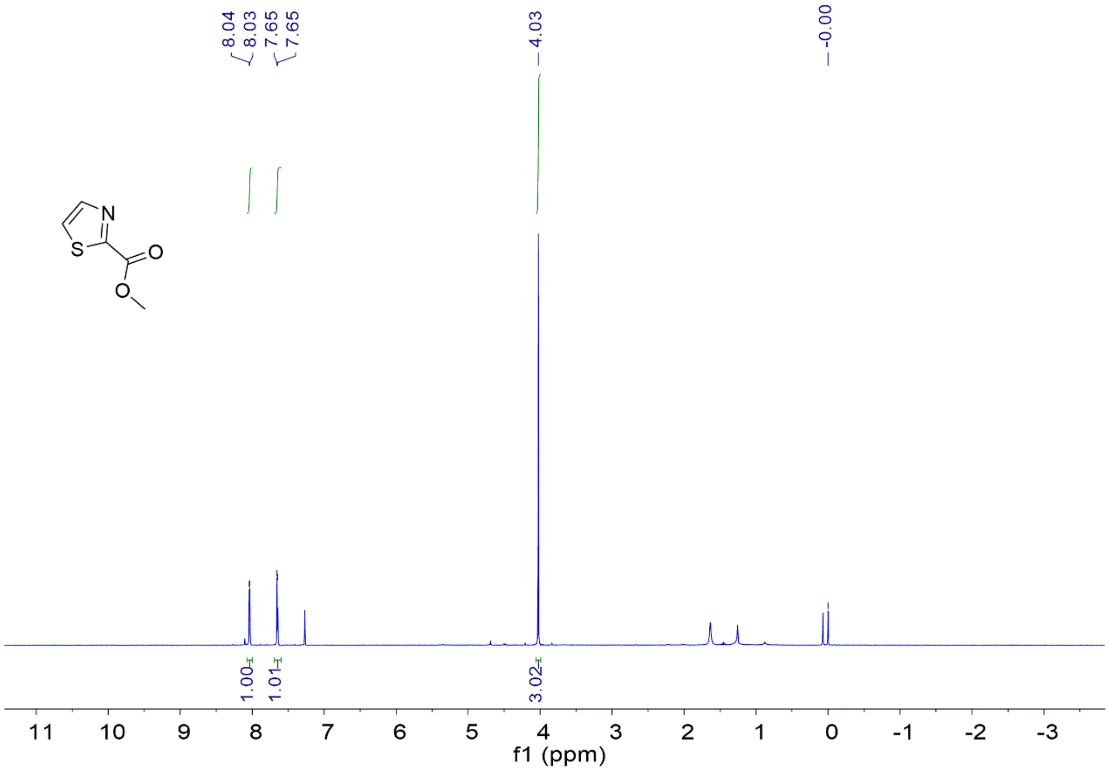


## $^{13}$C-NMR spectrum of 3ta

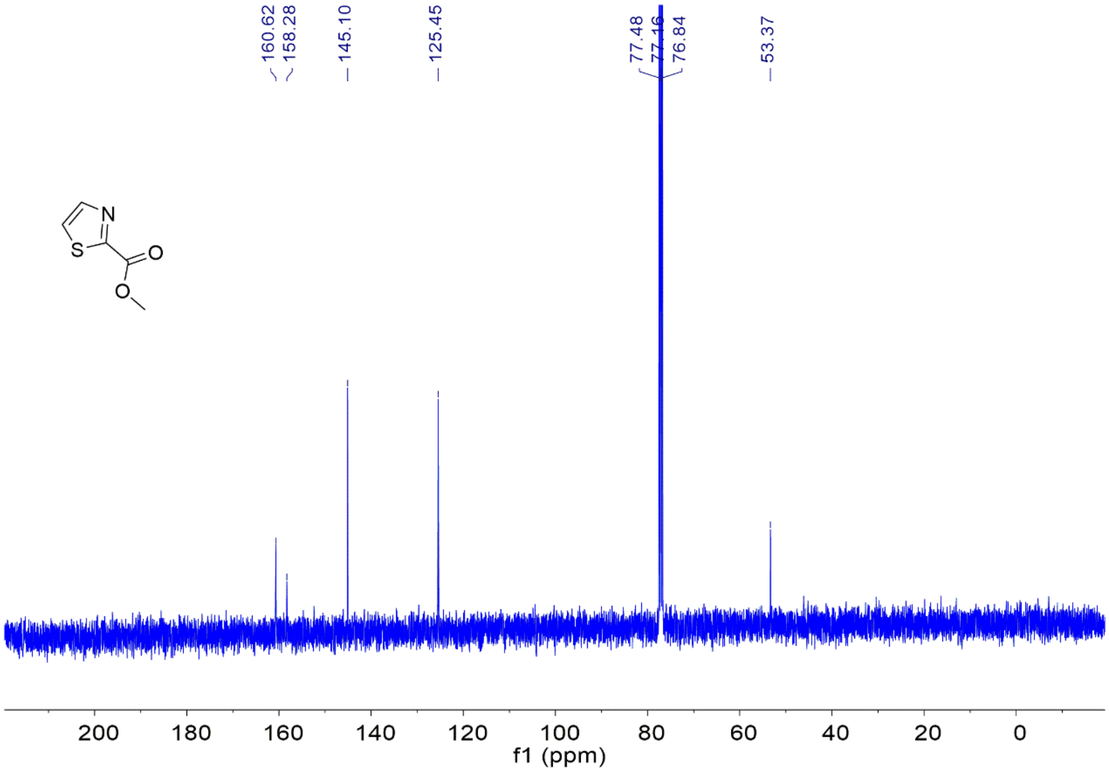

$^{1}$H-NMR spectrum of 3ua

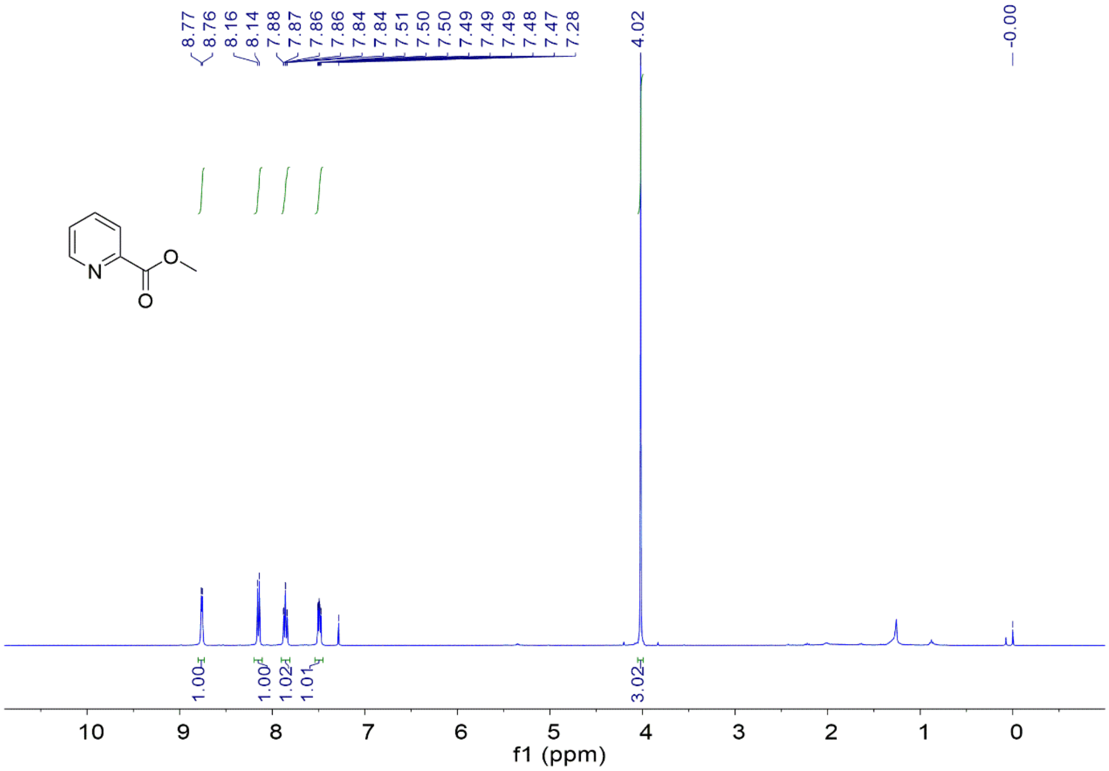


$^{13}$C-NMR spectrum of 3ua

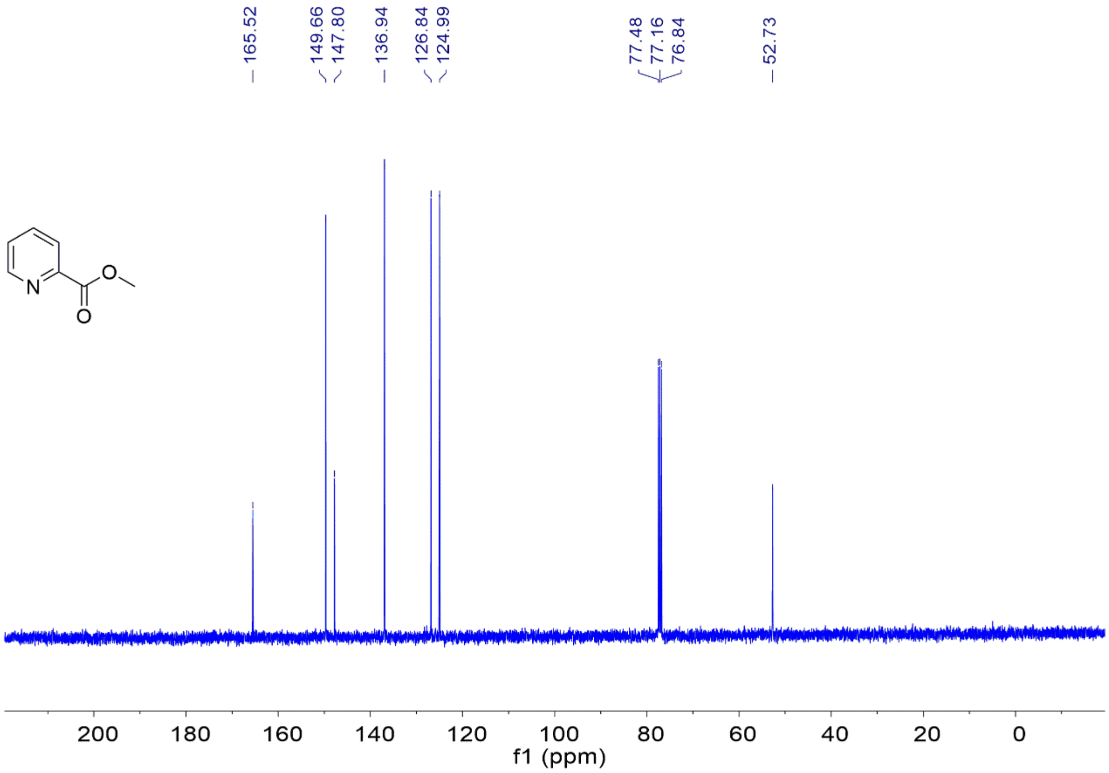

**$^{1}$H-NMR spectrum of 3va**

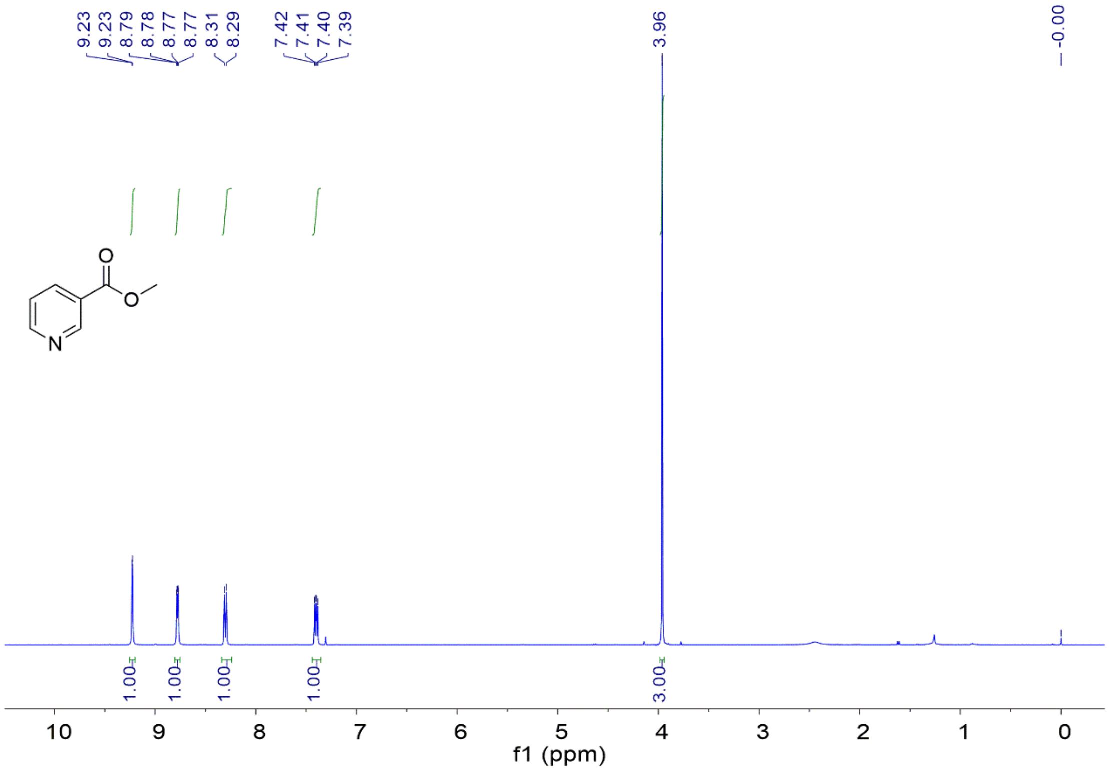


**$^{13}$C-NMR spectrum of 3va**

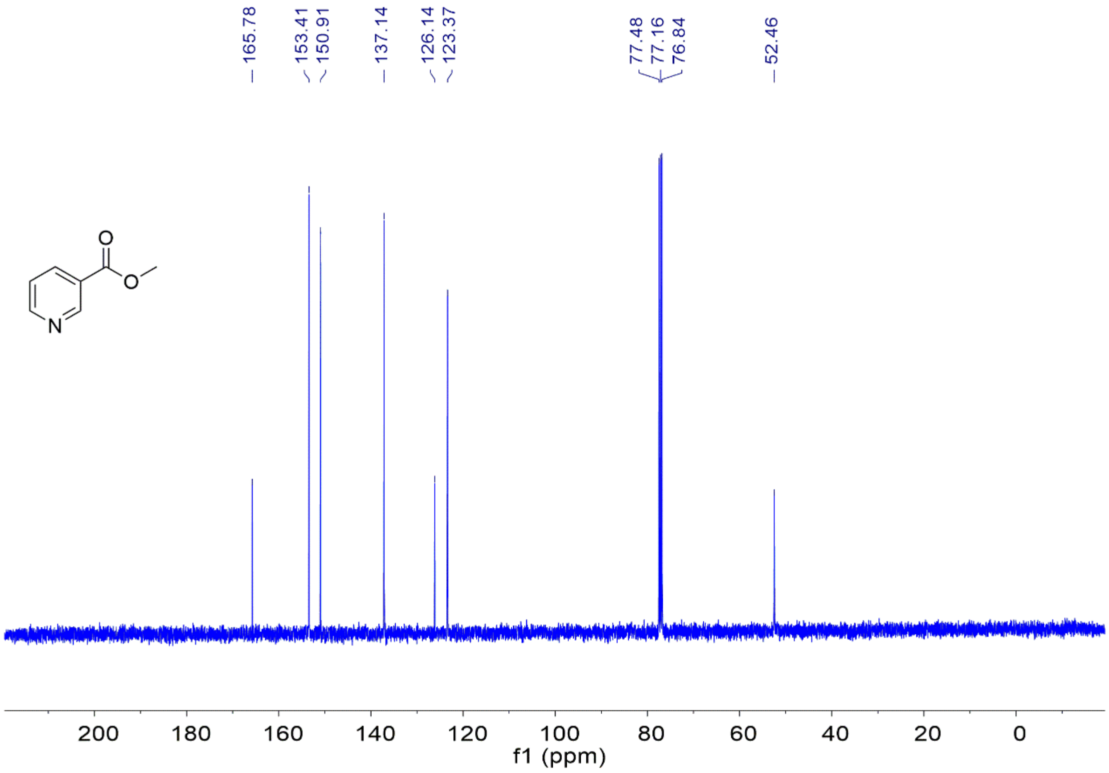

## $^{1}$H-NMR spectrum of 3wa

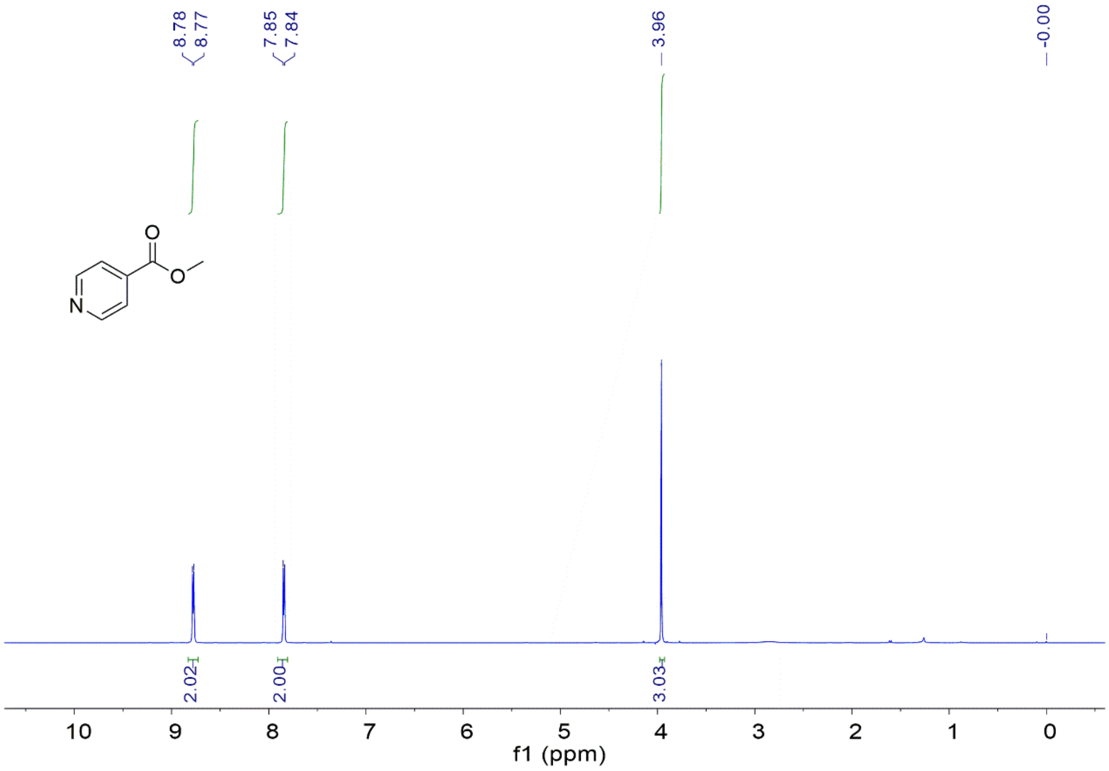


## $^{13}$C-NMR spectrum of 3wa

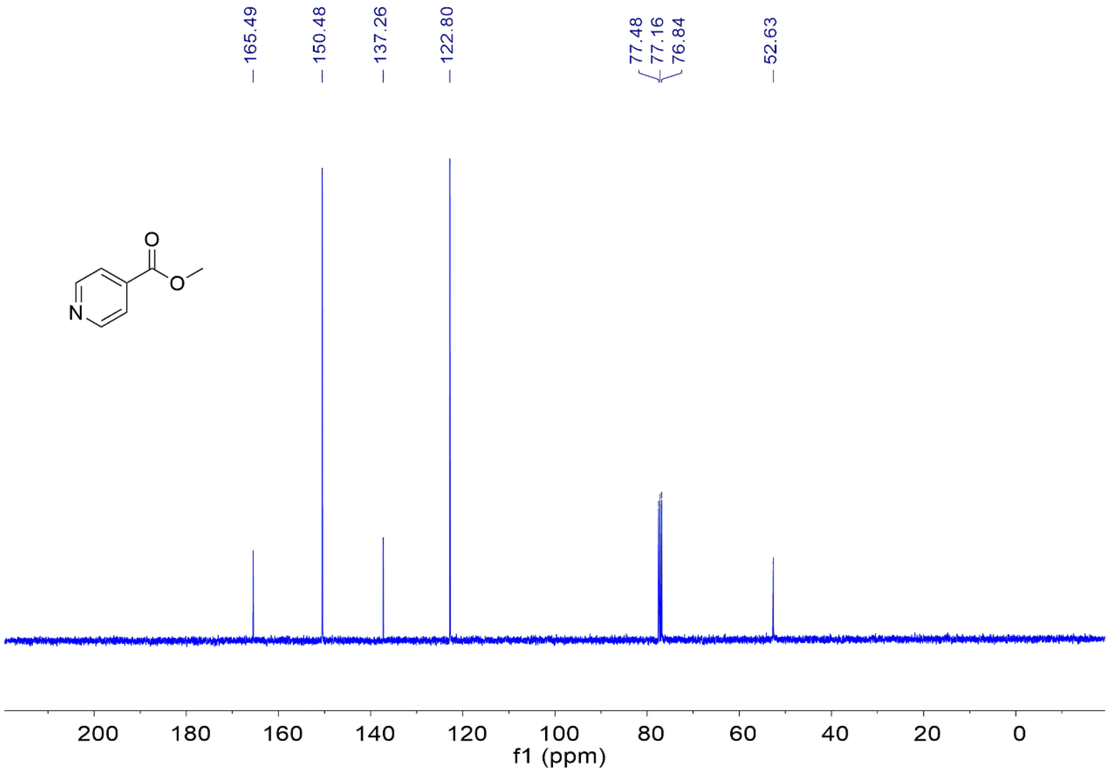

## $^{1}$H-NMR spectrum of 3xa

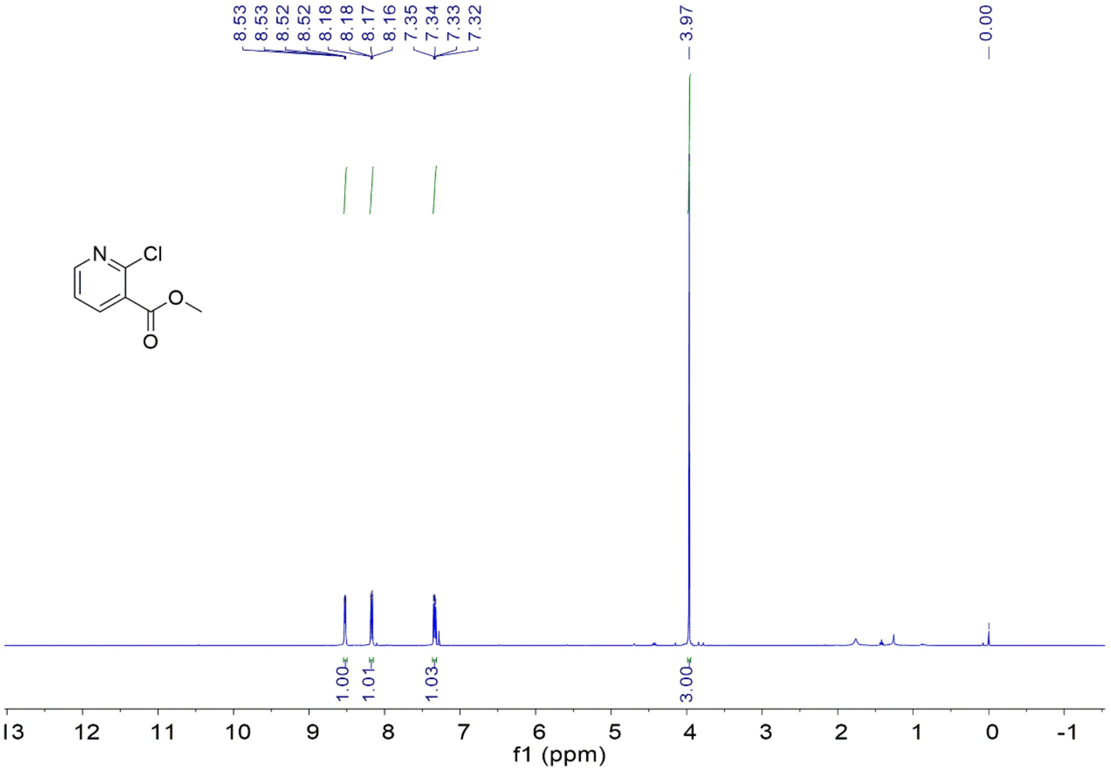


## $^{13}$C-NMR spectrum of 3xa

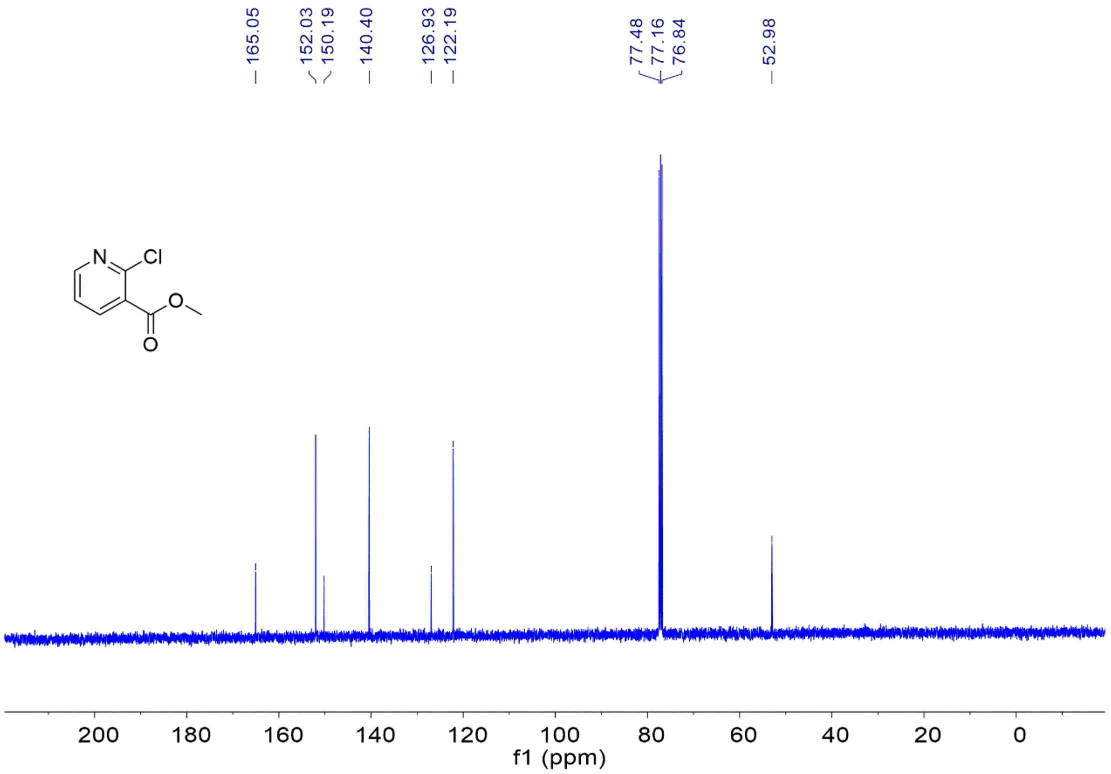

$^{1}$H-NMR spectrum of 3ya

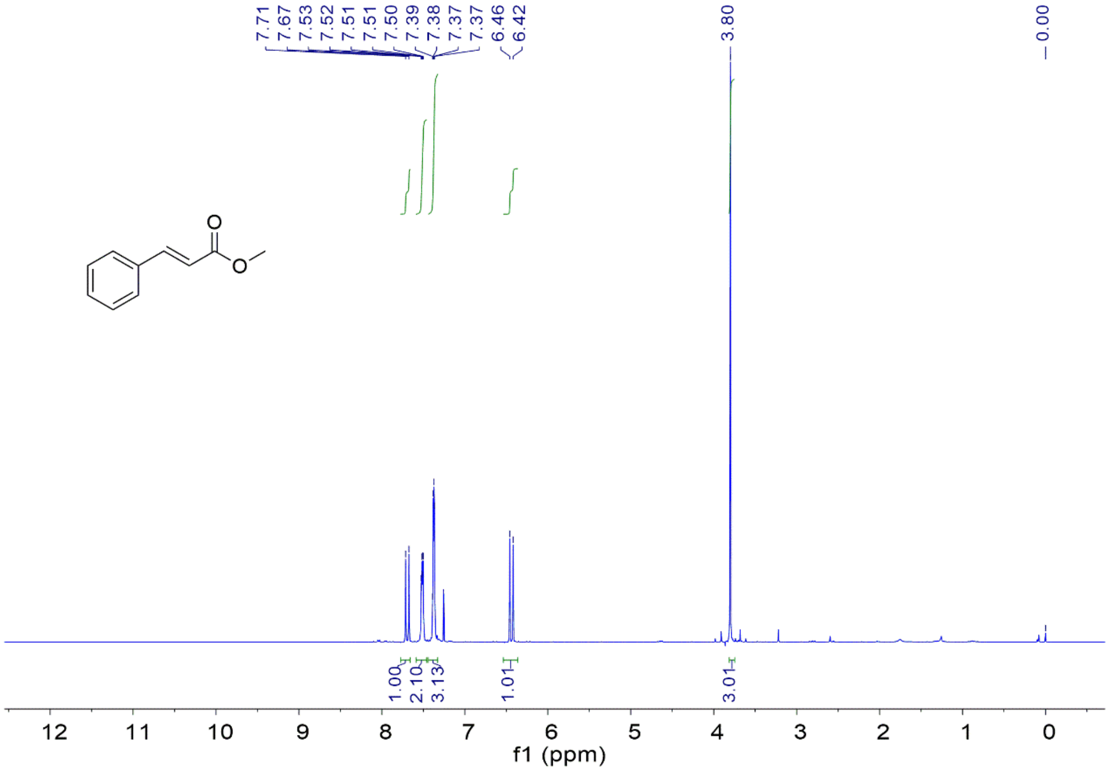


$^{13}$C-NMR spectrum of 3ya

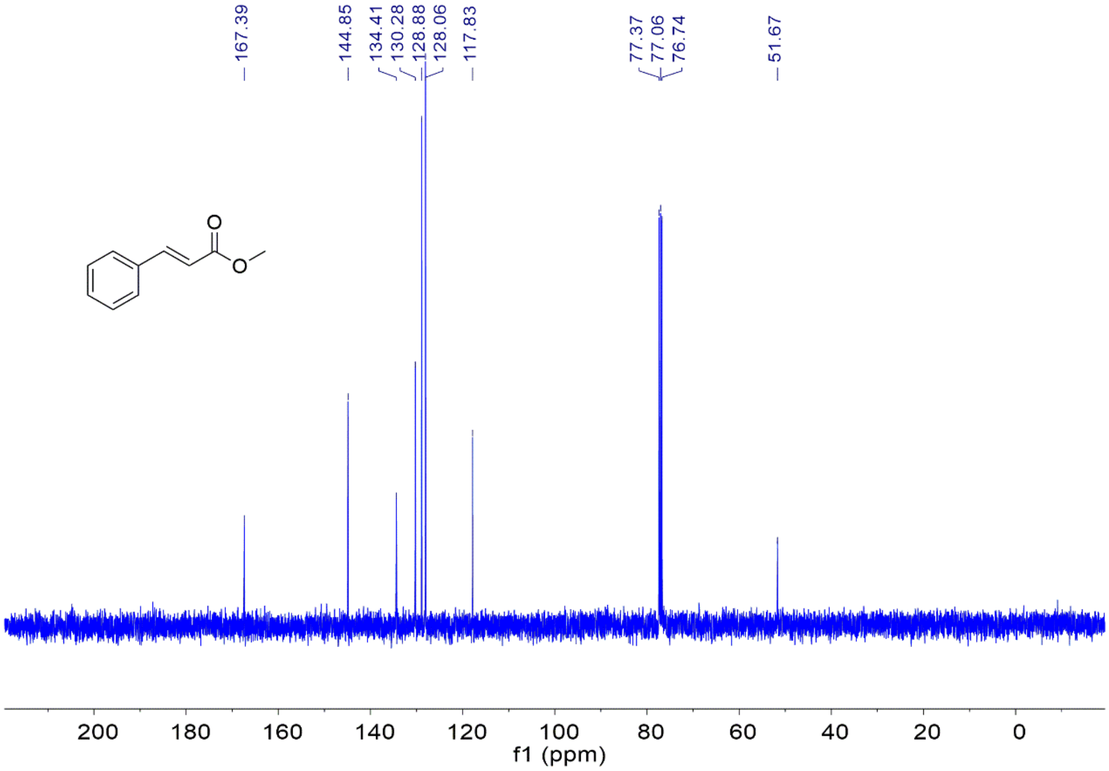

**$^{1}$H-NMR spectrum of 3ab**

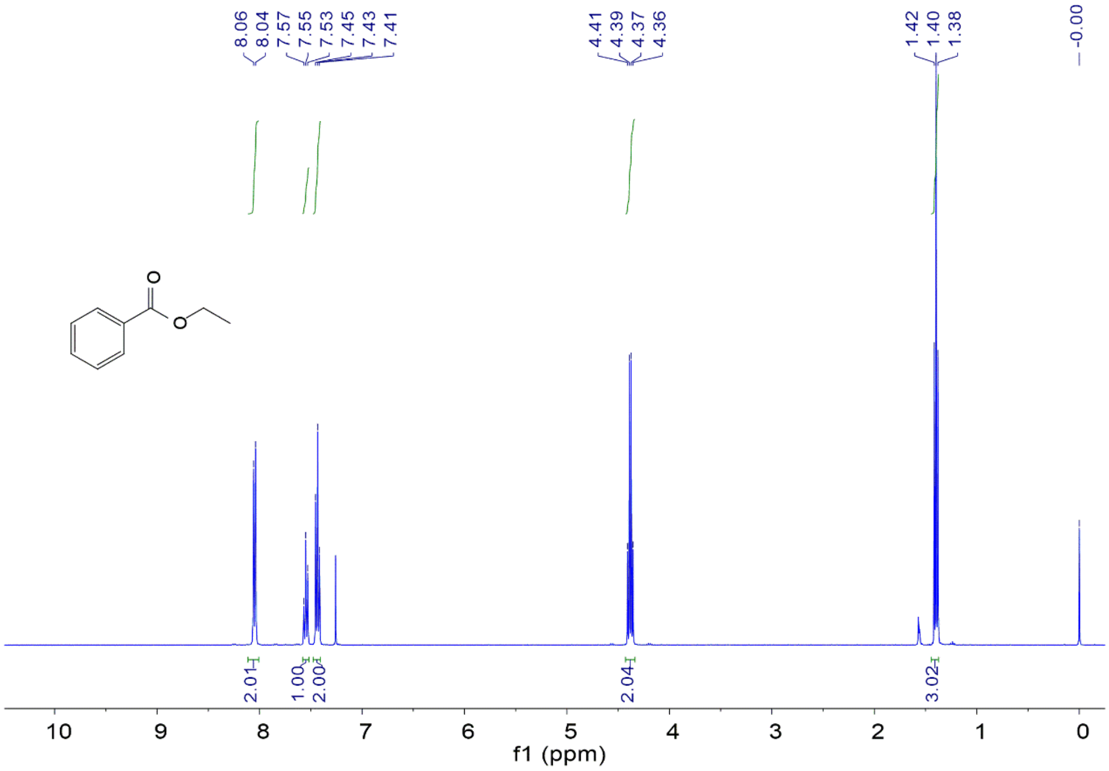


**$^{13}$C-NMR spectrum of 3ab**

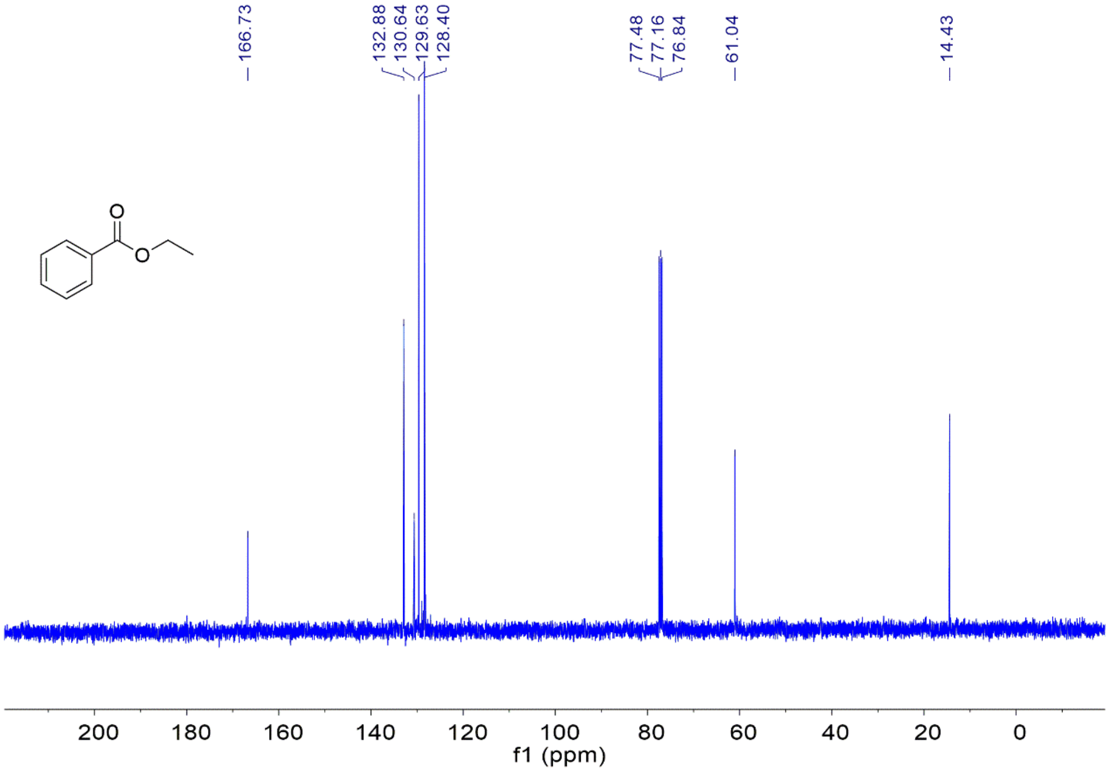

## $^{1}$H-NMR spectrum of 3ob

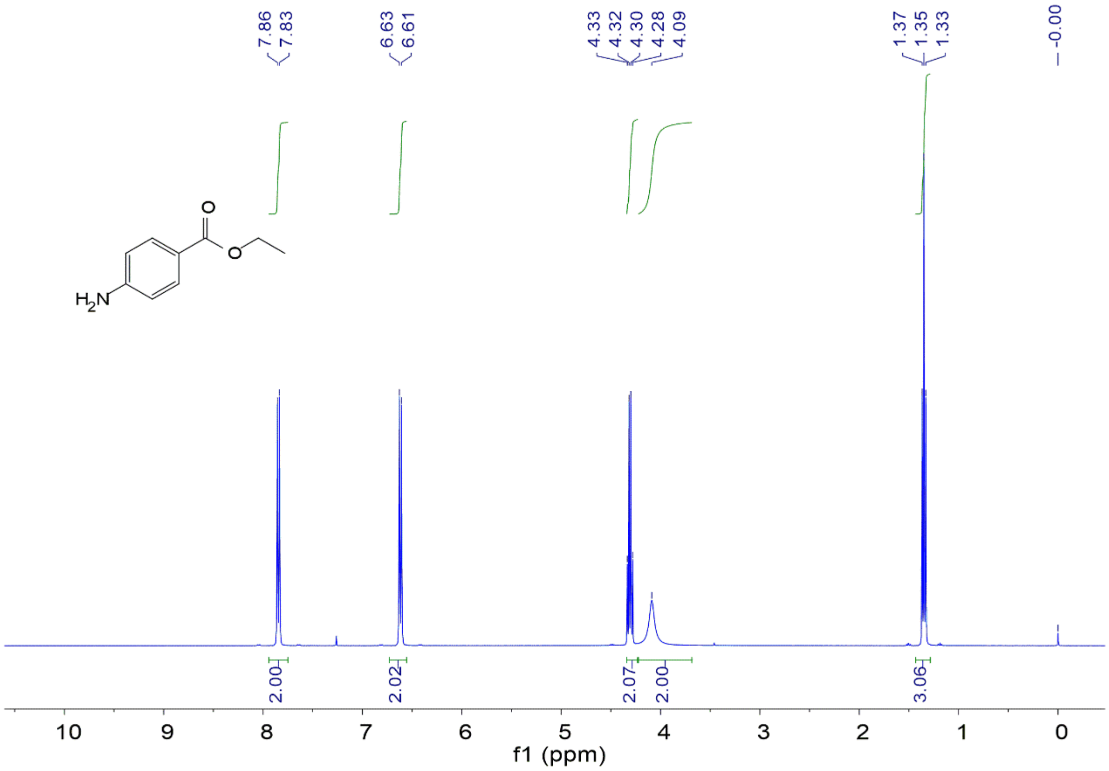


## $^{13}$C-NMR spectrum of 3ob

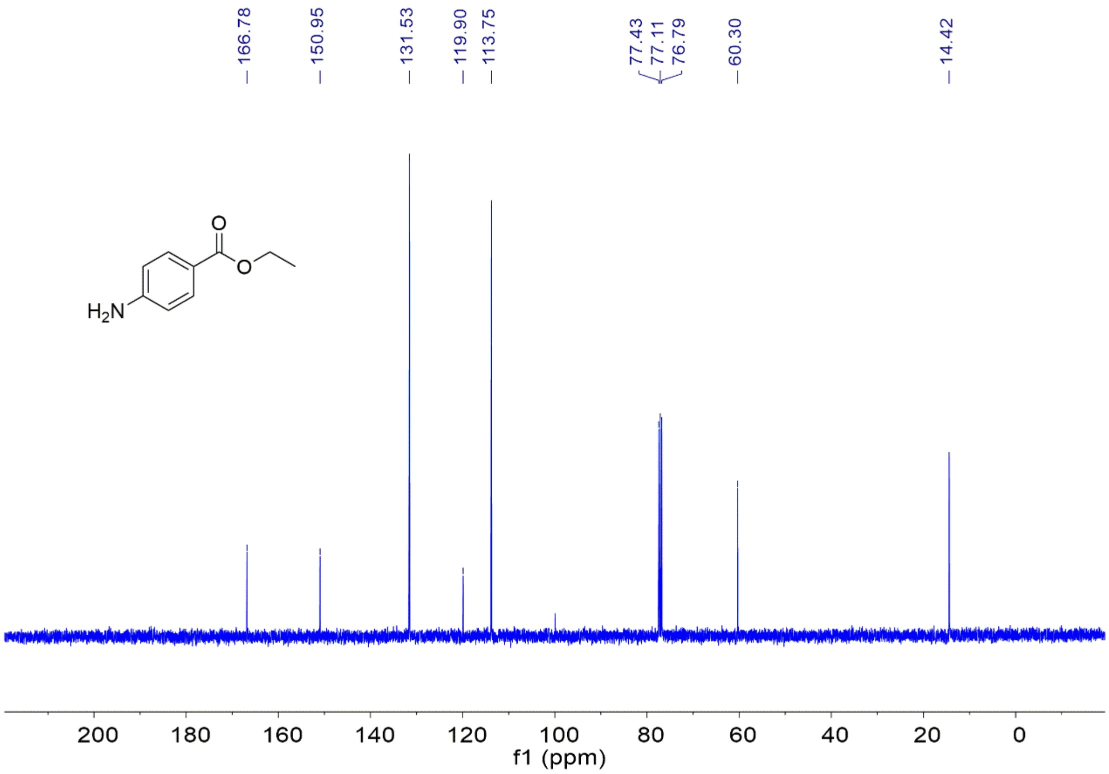

## $^{1}$H-NMR spectrum of 3ac

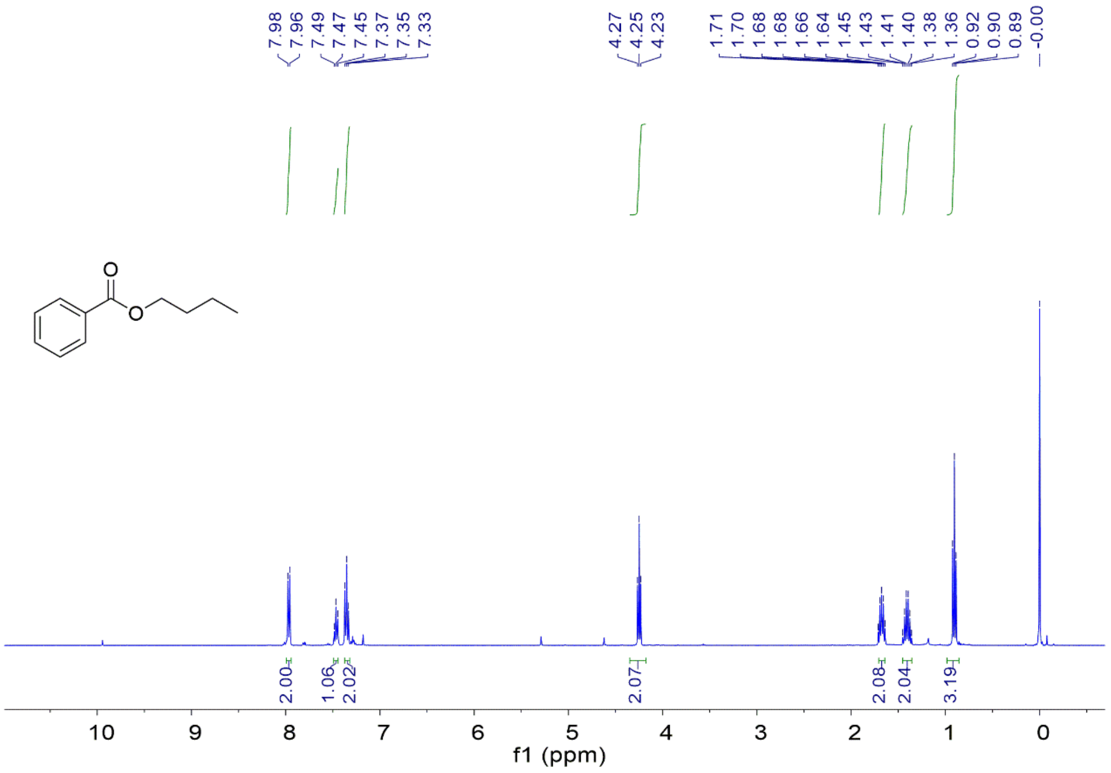


## $^{13}$C-NMR spectrum of 3ac

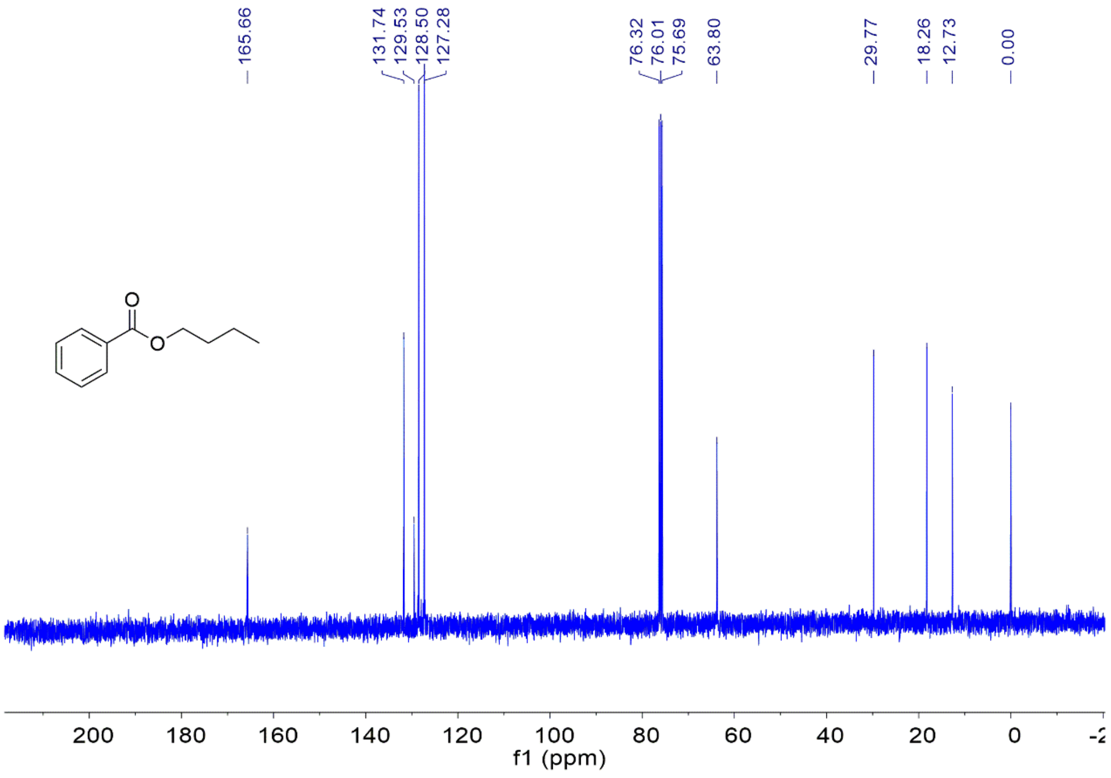

## $^{1}$H-NMR spectrum of 3ad

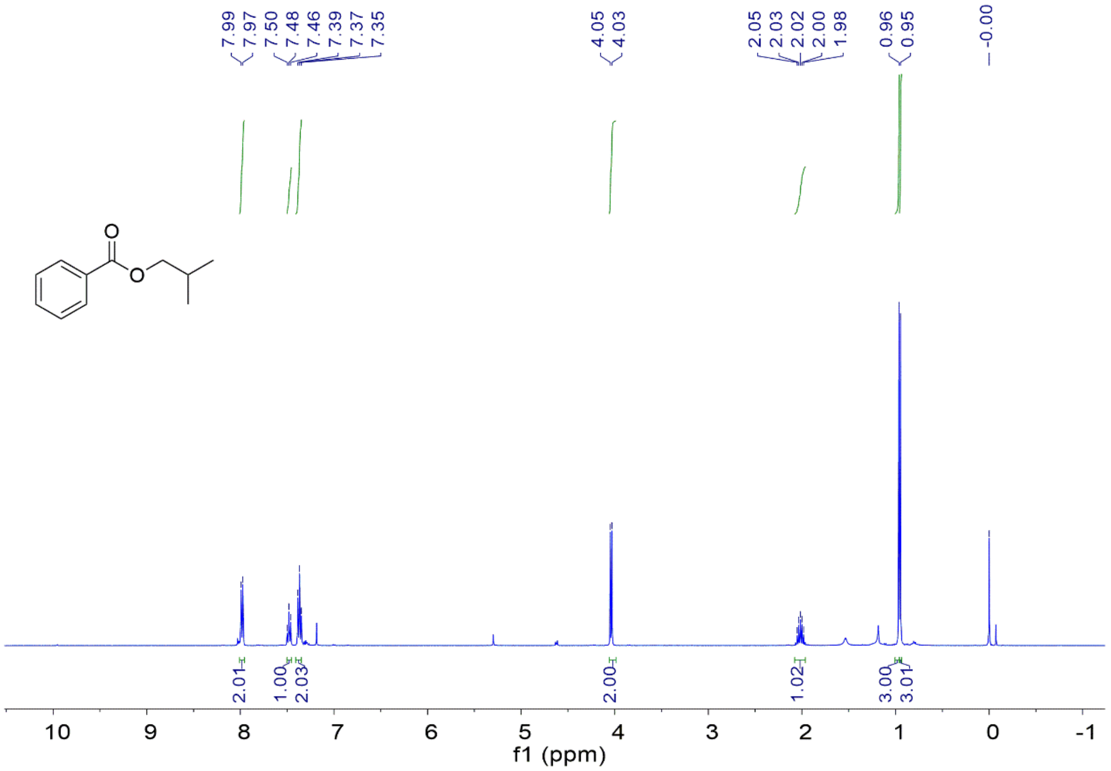


## $^{13}$C-NMR spectrum of 3ad

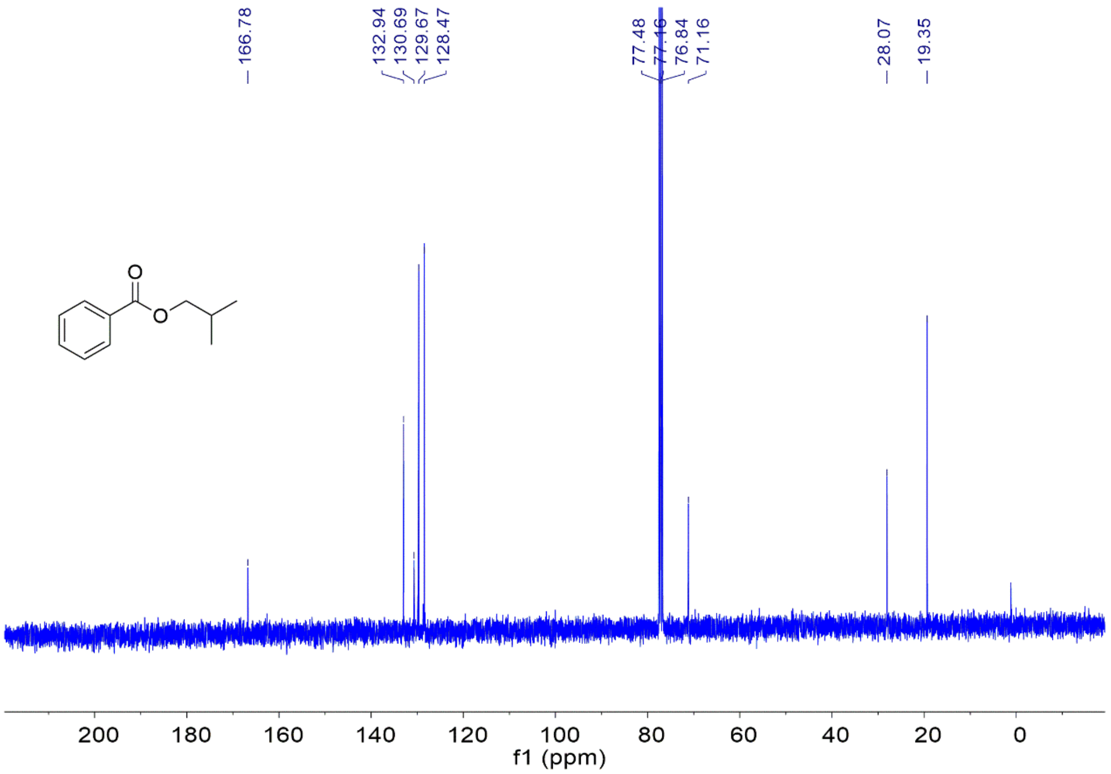

## $^{1}$H-NMR spectrum of 3za

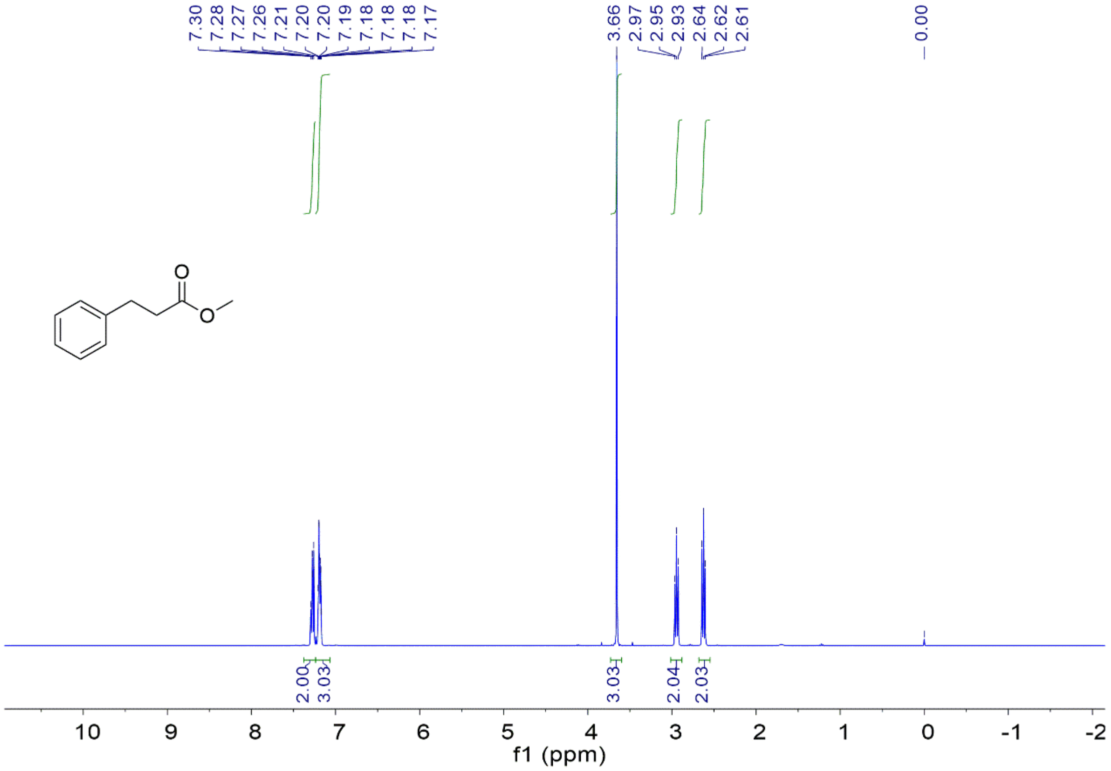


## $^{13}$C-NMR spectrum of 3za

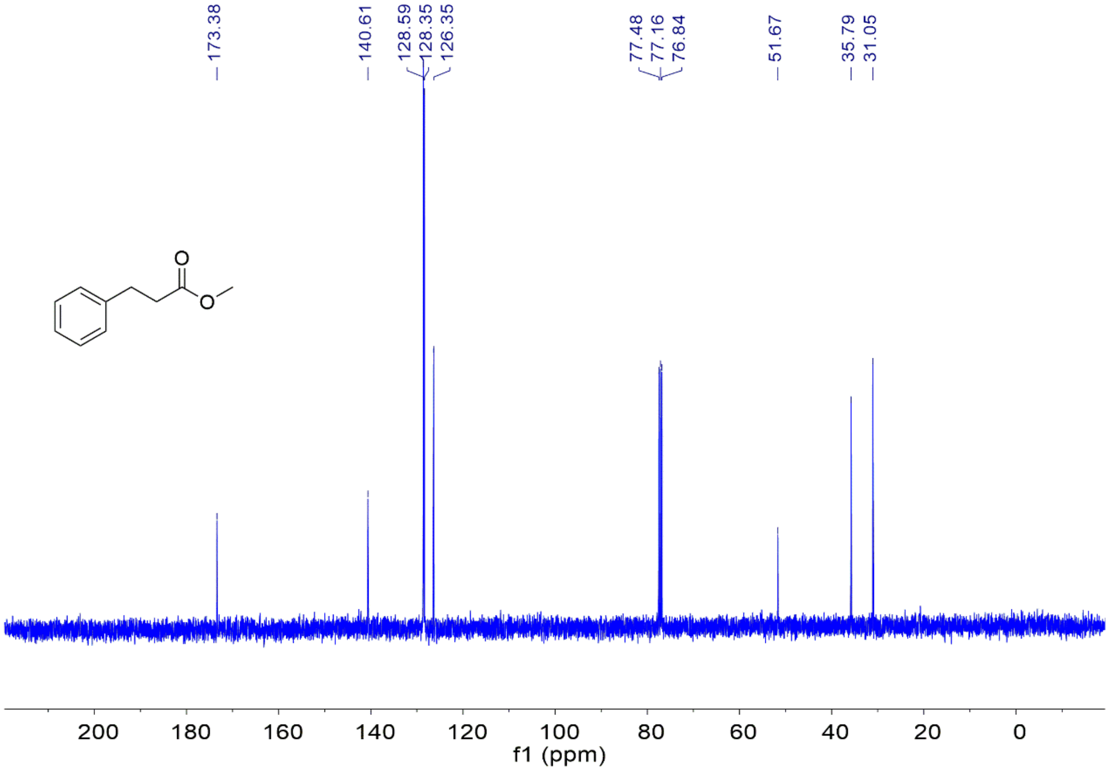

Supplement: Supplementary file 1 [file molecules-26-06792-s001.zip › molecules-1438306-supplementary.pdf]
